# Supplementary material for: A fully conjugated meso-boron-substituted porphyrinoid combining Lewis acidity with redox-switchable aromaticity
Source: Nat Commun. 2025 Feb 19;16:1775. doi: 10.1038/s41467-025-56892-w (PMC11840099; doi:10.1038/s41467-025-56892-w)
Supplement: Supplementary file 1 — Supplementary Information [file 41467_2025_56892_MOESM1_ESM.pdf]

Supplementary Information

for

A Fully Conjugated *Meso*-Boron-Substituted Porphyrinoid Combining Lewis Acidity with Redox-Switchable Aromaticity

Manuel Buckel[+1], Jonas Klopf[+1], Johannes S. Schneider[1], Artur Lik[1], Nicolas A. Riensch[1], Ivo Krummenacher[1], Holger Braunschweig[1], Bernd Engels*[2] & Holger Helten*[1]

[1] Julius-Maximilians-Universität-Würzburg, Institute of Inorganic Chemistry and Institute for Sustainable Chemistry & Catalysis with Boron (ICB), Am Hubland, 97074 Würzburg, Germany.

[2] Julius-Maximilians-Universität Würzburg, Institute for Physical and Theoretical Chemistry, Emil-Fischer-Strasse 42, 97074 Würzburg, Germany.

[+]These authors contributed equally to this work.

E-Mail: bernd.engels@uni-wuerzburg.de, holger.helten@uni-wuerzburg.de

Table of Contents

Supplementary Methods 3

General Procedures 3

Syntheses 6

NMR Spectra 18

High Resolution Mass Spectra 25

UV-vis and Fluorescence Spectra 30

Cyclic Voltammetry 39

Electron Paramagnetic Resonance Spectra 44

Crystallographic Data 46

Computational methods 59

Computational Results 61

Supplementary References 89

Supplementary Methods

General Procedures

Unless otherwise stated, all reagents were purchased from commercial suppliers and used without further purification. All reactions were performed under argon atmosphere using standard Schlenk techniques or an MBraun glovebox. *n*-Pentane and methanol were dried and degassed according to general purification methods. Dichloromethane, *n*-hexane, toluene, Et_2O and THF were degassed and dried by means of a Pure Solve PS-MD-5 solvent purification system by *Innovative Technology*. Deuterated solvents were purchased from Sigma Aldrich, dried over Na (C_6D_6, THF-d_8) or CaH_2 ($CDCl_3$), distilled and stored under argon. Trimethylsilylchloride, purchased from Sigma Aldrich, was dried over CaH_2, and distilled prior to use. Trimethylsilylmethoxide, TMEDA, 2-bromothiophene, di-*iso*-propylamine, tribromoborane trimethylborate and thiophene, purchased from Sigma Aldrich, were distilled prior to use. KC_8 was synthesized by stirring one equivalent of clean and dry potassium pieces in eight equivalents of dry graphite while heating to 145 °C. 2,4,6-Tri-*iso*-propylphenyllithium[1], **1**[2], **5**[2], 2,5-bis(trimethylsilyl)thiophene[2], **8**[3], **9**[4], and dimethyl-(2,4,6-tri-*iso*-propylphenyl)-borate[5] were synthesized according to literature procedures.

NMR spectra were recorded at 298 K on a Bruker Avance III (operating at ^{1}H: 300 MHz, ^{11}B: 96 MHz) or a Bruker Avance 500 FT NMR spectrometer (operating at ^{1}H: 500 MHz, ^{11}B: 160 MHz, ^{13}C: 126 MHz) at 296 K. Chemical shifts (δ) were referenced to residual protic impurities in the solvent (^{1}H) or the deuterated solvent itself (^{13}C) and reported relative to external $SiMe_4$ (^{1}H, ^{13}C) or $BF_3{\cdot}OEt_2$ (^{11}B) standards.

Mass spectra were obtained with the use of a Thermo Scientific Exactive Plus Orbitrap MS system with electron spray ionization (ESI) or by liquid injection field desorption ionization (LIFDI). All MS spectra obtained showed excellent congruence with the calculated isotopic distribution patterns.

Elemental analyses were performed on an Elementar vario MICRO cube elemental analyzer.

The optical measurements were performed in standard quartz cuvettes (1 cm x 1 cm cross-section). UV-vis absorption spectra were recorded using a Perkin Elmer

LAMBDA 465 UV-vis spectrophotometer or a Mettler Toledo UV7 spectrophotometer in a nitrogen-atmosphere in a MBraun glovebox. The measurements reaching into the NIR-region of the spectrum were recorded using a Perkin Elmer LAMBDA 1050+ UV/Vis/NIR spectrophotometer. The emission spectra were recorded using an Edinburgh Instruments FLS920 spectrometer equipped with a double monochromator for both excitation and emission, operating in right-angle geometry mode, and all spectra were fully corrected for the spectral response of the instrument. Fluorescence quantum yields were measured using a calibrated integrating sphere from Edinburgh Instruments combined with the FLS920 spectrometer described above.

All cyclic voltammetry (CV) and differential pulse voltammetry (DPV, square wave) experiments were conducted in a nitrogen-filled glovebox using a Gamry Instruments 1010B potentiostat. A standard three-electrode cell configuration was employed using a platinum disk working electrode (d = 3 mm), a platinum wire counter electrode, and a silver wire reference electrode separated by a Vycor® frit, serving as a pseudo-reference electrode. The redox potentials are referenced to the ferrocene/ferrocenium ([Fc/Fc^+]) redox couple) as an internal standard. Tetra-*n*-butylammonium hexafluorophosphate ([$n$$Bu_4N$][$PF_6$]) was employed as the supporting electrolyte with a concentration of 0.1 mol/L.

Crystals suitable for single-crystal X-ray diffraction were selected and coated in perfluoropolyether oil. Diffraction data were collected on Bruker X8 Apex II 4-axis-κ-goniometer diffractometer with APEX II CCD area detector using Mo-K_α radiation or Bruker AXS D8-Quest 4-axis-κ-goniometer diffractometer with Photon II CMOS area detector using Mo-K_α radiation. The crystals were cooled using Oxford Cryostreams low-temperature devices. Data were collected at 100 K. The images were processed and corrected for Lorentz-polarization effects and absorption as implemented in the Bruker software packages. The structures were solved using the intrinsic phasing method (SHELXT)[6] and Fourier expansion technique. All non-hydrogen atoms were refined in anisotropic approximation, with hydrogen atoms ‘riding’ in idealized positions, by full-matrix least squares against F^2 of all data, using SHELXL[7] software and the SHELXLE graphical user interface.[8] Crystallographic data have been deposited with the Cambridge Crystallographic Data Center as supplementary publication no. CCDC-2333888 (**4**), CCDC-2333889 (**9**), CCDC-2333890 (**2**), CCDC-2333891 (**K_2[4]**), CCDC-2333892 (**K[4]**) and CCDC-2333893 (**10**). These data can be

obtained free of charge from the Cambridge Crystallographic Data Center *via* www.ccdc.cam.ac.uk/data_request/cif.

EPR measurements at X-band (9.37 GHz for **Li[1]**, **K[4]**/ 9.85 GHz for **K[2]**, **K[3]**; microwave power: 2 mW for **Li[1]**, **K[4]**/ 0.2 mW for **K[2]**, **K[3]**) were carried out using a Bruker ELEXSYS E580 CW EPR spectrometer. Modulation amplitude: 0.5 G; Conversion time: 60 ms; modulation frequency: 100 kHz. The spectral simulations were performed using MATLAB 9.8.0.1323502 (R2020a) or 9.13.0.2105380 (R2022b) and the EasySpin 5.2.35 toolbox.[9]

Li[1], **K[2]**, **K[3]**, **K[4]** were prepared by addition of small amounts of KC_8 or lithium naphthalenide respectively to the sample-solution in THF.

Syntheses

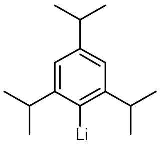

Synthesis of (2,4,6-tri-*iso*-propylphenyl)lithium (literature known[1]):

To a solution of 1-bromo-2,4,6-tri-*iso*-propylbenzene (25.5 g, 90.0 mmol) in *n*-hexane (300 mL) *n*-BuLi (135 mmol, 12.3 mL) in *n*-hexane was added dropwise. The reaction mixture was stirred at 60 °C for 16 h. The suspension was filtered off and the filtrate was reduced to half of its volume. The product was precipitated at –30 °C, filtered off and washed with cold *n*-pentane and dried *in vacuo*. It was obtained as a colorless solid.

Yield: 9.31 g (44.3 mmol, 49 %).
^{1}H-NMR (300 MHz, C_6D_6): δ = 7.08 (s, 2H, Tip-*H*), 2.86-2.61 (-, 3H, ^{i}Pr-C*H*), 1.35 (d, 12H, $^3J_{HH}$ = 6.9 Hz, *o*-^{i}Pr-CH_3), 1.23 (d, 6H, $^3J_{HH}$ = 6.9 Hz, *p*-^{i}Pr-CH_3) ppm.

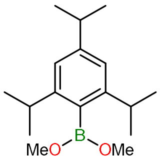

Synthesis of dimethyl-(2,4,6-tri-*iso*-propylphenyl)borate (literature known[5]):

1-Bromo-2,4,6-tri-*iso*-propylbenzene (14.4 g, 50.9 mmol) in THF (40 mL) was added dropwise to neat magnesium within 15 min. The mixture was stirred under reflux for 2 h. The resulting suspension was filtered off. The filtrate was added at –78°C to a solution of $B(OMe)_3$ (17.5 g, 168 mmol) in Et_2O. The mixture was stirred for 3 d and filtered off and the product was extracted from the residue with *n*-pentane. The solvent was removed under reduced pressure and the crude product was purified by distillation to yield a colorless liquid.

Yield: 8.78 g (31.8 mmol, 63 %).
^{1}H-NMR (300 MHz, $CDCl_3$): δ = 6.99 (s, 2H, Tip-*H*), 3.60 (s, 6H, -O-CH_3), 2.90 (sept, 1H, $^3J_{HH}$ = 6.8 Hz, *p*-^{i}Pr-C*H*), 2.67 (sept, 2H, $^3J_{HH}$ = 6.8 Hz, *o*-^{i}Pr-C*H*), 1.32-1.23 (-,

18H, ^{i}Pr-C*H*3) ppm.

^{11}B{1H}-NMR (96 MHz, CDCl$_3$): δ = 32.1 (s) ppm.

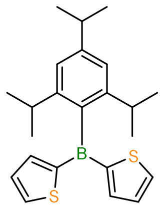

Synthesis of di(thien-2-yl)(2,4,6-tri-*iso*-propylphenyl)borane 1 (literature known[2]):

n-BuLi (66.8 mmol, 26.7 mL) in *n*-hexane was added dropwise to a solution of 2-bromothiophene(10.4 g, 63.6 mmol) in *n*-hexane (200 mL) at room temperature. The reaction mixture was stirred for 3 h. Then it was added at –78 °C to a solution of dimethyl-(2,4,6-tri-*iso*-propylphenyl)borate (8.78 g, 31.8 mmol) in *n*-hexane (50 mL). The mixture was stirred for 2 d. The mixture was worked up aqueously. The raw product was extracted with *n*-pentane and washed with brine and water and then dried over $MgSO_4$. It was filtered off and the solvent was removed under reduced pressure. The crude product was purified by column chromatography (silica, PE) to yield a colorless solid.

Yield: 10.2 g (26.7 mmol, 84 %).

^{1}H-NMR (300 MHz, CDCl$_3$): δ = 7.91 (dd, 2H, $^3J_{HH}$ = 4.7 Hz, $^4J_{HH}$ = 1.0 Hz, Th-*H*), 7.84 (dd, 2H, $^3J_{HH}$ = 3.6 Hz, $^4J_{HH}$ = 1.0 Hz, Th-*H*), 7.29 (dd, 2H, $^3J_{HH}$ = 3.6 Hz, $^3J_{HH}$ = 4.7 Hz, Th-*H*), 6.99 (s, 2H, Tip-*H*), 2.94 (sept, 1H, $^3J_{HH}$ = 7.0 Hz, *p*-^{i}Pr-C*H*), 2.50 (sept, 2H, $^3J_{HH}$ = 6.8 Hz, *o*-^{i}Pr-C*H*), 1.32 (d, 6H, $^3J_{HH}$ = 6.9 Hz, *p*-^{i}Pr-CH_3), 1.04 (d, 12H, $^3J_{HH}$ = 6.8 Hz, *o*-^{i}Pr-CH_3) ppm.

^{11}B{^{1}H}-NMR (96 MHz, CDCl$_3$): δ = 56.9 (br, Ar_3B) ppm.

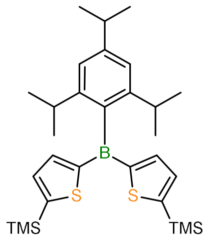

Synthesis of di(5-trimethylsilylthien-2-yl)(2,4,6-tri-*iso*-propylphenyl)borane 5 (literature known[2]):

A solution of **1** (380 mg, 1.00 mmol) in Et_2O (7.5 mL) was prepared and *t*-BuLi (2.1 mmol, 1.24 mL) in *n*-pentane was added dropwise at -78 °C. The mixture was stirred for 4 h. Then, chlorotrimethylsilane (240 mg, 2.2 mmol) was added dropwise and it was stirred overnight. The mixture was worked up aqueously. The raw product was extracted with Et_2O and washed with brine and water and then dried over $MgSO_4$. It was filtered off and the solvent was removed under reduced pressure. The crude product was purified by recrystallization from *n*-hexane/DCM. The product was obtained as a colorless solid.

Yield: 443 mg (0.84 mmol, 84 %).
^{1}H-NMR (300 MHz, $CDCl_3$): δ = 7.81 (d, 2H, $^3J_{HH}$ = 3.4 Hz, Th-*H*), 7.38 (d, 2H, $^3J_{HH}$ = 3.4 Hz, Th-*H*), 6.99 (s, 2H, Tip-*H*), 2.95 (sept, 1H, $^3J_{HH}$ = 6.9 Hz, *p*-^{i}Pr-C*H*), 2.49 (sept, 2H, $^3J_{HH}$ = 6.9 Hz, *o*-^{i}Pr-C*H*), 1.33 (d, 6H, $^3J_{HH}$ = 6.9 Hz, *p*-^{i}Pr-CH_3), 1.03 (d, 12H, $^3J_{HH}$ = 6.7 Hz, *o*-^{i}Pr-CH_3), .036 (s, 18H, -Si-CH_3) ppm.
^{11}B{^{1}H}-NMR (96 MHz, $CDCl_3$): δ = 55.6 (br, Ar_3B) ppm.

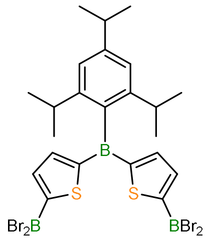

Synthesis of (((2,4,6-tri-*iso*-propylphenyl)boranediyl)bis(thiophene-5,2-diyl))bis-(dibromoborane) 6:

A solution of **5** (5.44 g, 10.4 mmol) in DCM (40 mL) was cooled to 0 °C and neat BBr_3 (6.49 g, 25.9 mmol) was added dropwise. The mixture was stirred for 2 h maintaining the low temperature. Then all volatiles were removed *in vacuo*. To that solid residue

n-pentane (50 mL) was added and the mixture was crystallized at −30 °C for 3 d. The solid was filtered off, washed with cold *n*-pentane and subsequently dried *in vacuo* to yield a yellow solid.

Yield: 6.03 g (8.38 mmol, 81 %).

^{1}H-NMR (500 MHz, CDCl$_3$): δ = 8.14 (d, 2H, $^3J_{HH}$ = 3.8 Hz, outer Th-*H*), 7.96 (d, 2H, $^3J_{HH}$ = 3.8 Hz, inner Th-*H*), 7.02 (s, 2H, Tip-*H*), 2.96 (sept, 1H, $^3J_{HH}$ = 6.9 Hz, *p*-^{i}Pr-*H*), 2.35 (sept, 2H, $^3J_{HH}$ = 6.7 Hz, *o*-^{i}Pr-*H*), 1.33 (d, 6H, $^3J_{HH}$ = 7.0 Hz, *p*-^{i}Pr-C*H*$_3$), 1.05 (d, 12H, $^3J_{HH}$ = 6.7 Hz, *o*-^{i}Pr-C*H*$_3$) ppm.

^{13}C-NMR (126 MHz, CDCl$_3$): δ = 159.4 (br, *α*-Th-*C*-BBr$_2$) 154.6 (br, *α*-Th-*C*–B), 150.0 (q *C* next to *p*-^{i}Pr), 149.7 (q *C* next to *o*-^{i}Pr), 143.8 (outer *β*-Th-*C*H), 143.2 (inner *β*-Th-*C*H), 137.4 (br, q Tip *C*-B), 120.7 (arom. Tip *C*-H), 36.2 (*o*-^{i}Pr-*C*-H), 34.4 (*p*-^{i}Pr-*C*-H), 24.4 (*o*-^{i}Pr-*C*H$_3$), 24.2 (*p*-^{i}Pr-*C*H$_3$) ppm.

^{11}B{^{1}H}-NMR (160 MHz, CDCl$_3$): δ = 58.0 (br, Ar$_3$B), 49.4 (ArBBr$_2$) ppm.

HRMS (LIFDI) found: m/z = 719.8500 ([M]$^+$); calcd. for [$C_{23}H_{27}B_3Br_4S_2$]$^+$: 719.8520.

Elemental Analysis: Calcd (found) [%] for $C_{23}H_{27}B_3Br_4S_2$: C 38.39 (38.36), H 3.78 (3.93).

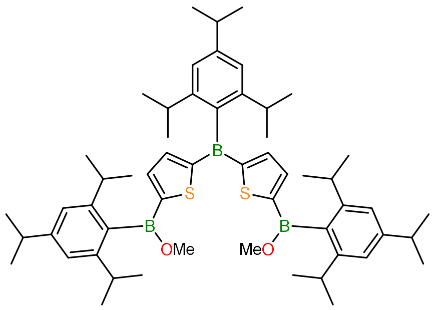

Synthesis of (((2,4,6-tri-*iso*-propylphenyl)boranediyl)bis(thiophene-5,2-diyl))bis-(methoxy(2,4,6-tri-*iso*-propylphenyl)borane) 7:

6 (5.04 g, 7.00 mmol) was dissolved in toluene (40 mL) and a solution of Tip-Li (3.02 g, 14.4 mmol) in toluene (10 mL) was added slowly. The mixture was stirred for 3 d. Then, the generated solid was filtered off and washed with toluene (50 mL). The organic phase was dried *in vacuo*. *n*-Hexane (50 mL) was added to it and the product was precipitated out of solution over the course of 1 d at −30 °C. The product was filtered off in the cold and washed with cold *n*-pentane. After drying *in vacuo*, a colorless solid was obtained. It was dissolved in DCM and TMS-OMe (459 mg, 4.40 mmol, 610 µL)

was added to the solution (1.70 g, 1.76 mmol) in DCM (7 mL). It was stirred for 2 h at room temperature. Afterwards all volatiles were removed *in vacuo* and the obtained solid was dissolved and subsequently crystallized out of a mixture of DCM/methanol at −30 °C. The product was filtered off, washed with cold methanol, and dried *in vacuo* to yield a colorless solid.

Yield: 1.38 g (1.59 mmol, 23 %).
^{1}H-NMR (500 MHz, $CDCl_3$): δ = 7.78 (d, ^3J = 3.6 Hz, 2H, outer Th-*H*), 7.44 (d, ^3J = 3.6 Hz, 2H, inner Th-*H*), 7.00 (s, 4H, outer Tip-*H*), 6.95 (s, 2H, inner Tip-*H*), 3.78 (s, 6H, O–CH_3), 2.97-2.87 (-, 3H, *p*-^{i}Pr-*H*), 2.60 (sept, ^3J = 6.8 Hz, 4H, outer *o*-^{i}Pr-*H*), 2.47 (sept, ^3J = 6.7 Hz, 2H, inner *o*-^{i}Pr-*H*), 1.31-1.27 (-, 18H, *p*-^{i}Pr-CH_3), 1.24 (d (br), ^3J = 6.1 Hz, 12H, outer *o*-^{i}Pr-CH_3), 1.05 (d (br), ^3J = 6.0 Hz, 12H, outer *o*-^{i}Pr-CH_3), 0.99 (d, ^3J = 6.7 Hz, 12H, inner *o*-^{i}Pr-CH_3) ppm
^{13}C-NMR (126 MHz, $CDCl_3$): δ= 153.9 (br, *α*-Th-*C*-BOMe) 152.8 (br, *α*-Th-*C*–B), 150.3 (q *C* next to *o*-^{i}Pr of outer Tip), 149.6 (q *C* next to *p*-^{i}Pr of inner Tip), 149.6 (q *C* next to *o*-^{i}Pr of inner Tip), 148.5 (q *C* next to *p*-^{i}Pr of outer Tip), 142.5 (outer *β*-Th-*C*H), 139.7 (br, inner q Tip-*C*–B), 139.5 (inner *β*-Th-*C*H), 132.8 (br, outer q Tip-*C*–B), 120.4 (outer arom. Tip-*C*H), 120.1 (inner arom. Tip-*C*H), 56.2 (O-*C*H_3), 35.6 (inner *o*-^{i}Pr-*C*H), 35.3 (outer *o*-^{i}Pr-*C*H), 34.4 (outer *p*-^{i}Pr-*C*H), 34.3 (inner *p*-^{i}Pr-*C*H), 24.7 (outer *o*-^{i}Pr-*C*H_3), 24.2 (inner *p*-^{i}Pr-*C*H_3), 24.2 (inner *o*-^{i}Pr-*C*H_3) ,24.2 (outer *p*-^{i}Pr-*C*H_3) ppm.
^{11}B{^{1}H}-NMR (160 MHz, $CDCl_3$): δ= 46.2 (Ar_2BOMe) ppm, Ar_3B: signal not detectable.
HRMS (LIFDI) found: m/z = 868.5785 ($[M]^+$); calcd. for $[C_{55}H_{79}B_3O_2S_2]^+$: 868.5795.
Elemental Analysis: Calcd (found) [%] for $C_{55}H_{79}B_3O_2S_2$: C 76.04 (75.42), H 9.17 (9.21).

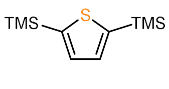

Synthesis of 2,5-bis(trimethylsilyl)thiophene (literature known[2]):

TMEDA (17.1 g, 147 mmol) was cooled to 0 °C and *n*-BuLi (174 mmol, 69.9 mL) in *n*-hexane was added dropwise. It was stirred for 15 min and thiophene (6.36 g, 75.6 mmol) in *n*-hexane (150 mL) was added dropwise. Then it was stirred for 30 min at room temperature and then under reflux for 45 min. Chlorotrimethylsilane (18.1 g, 166 mmol) was added dropwise at 0 °C. It was stirred overnight. After aqueous work

up the product was extracted with *n*-hexane and washed with brine. It was dried over $MgSO_4$ which was filtered off afterwards. The solvent was removed under reduced pressure and the raw product was purified by distillation (50 °C at $1x10^{-3}$ mbar), granting a colorless liquid.

Yield: 15.8 g (68.9 mmol, 91 %).
^{1}H-NMR (300 MHz, $CDCl_3$): δ = 7.32 (s, 2H, Th-*H*), 0.32 (s, 18H, -Si-CH_3) ppm.

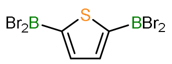

Synthesis of 2,5-bis(dibromoboryl)thiophene 8 (modified procedure of the literature[3]):

BBr_3 (11.6 g, 46.3 mmol) was added at 0 °C to a solution of 2,5-bis(trimethylsilyl)-thiophene (4.22 g, 18.5 mmol) in DCM (20 mL). The mixture was stirred overnight. The solution was reduced to a third of its volume under reduced pressure and the product was crystallized at –30 °C overnight. The precipitate was filtered off and washed with cold DCM. The solid was dried *in vacuo* to acquire the product as an off-white solid.

Yield: 7.37 g (17.4 mmol, 94 %).
^{1}H-NMR (300 MHz, $CDCl_3$): δ = 7.51 (s, 2H, Th-*H*) ppm.
^{11}B{^{1}H}-NMR (96 MHz, $CDCl_3$): δ = 49.6 ppm.

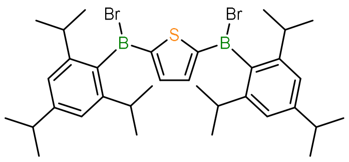

Synthesis of 2,5-bis[bromo(2,4,6-tri-*iso*-propylphenyl)boryl]thiophene 9 (literature known[4]):

A solution of Tip-Li (8.82 g, 42.0 mmol) in toluene (40 mL) was added to a mixture of **8** (8.47 g, 20.0 mmol) in toluene (40 mL) at 0 °C. It was stirred overnight. The precipitates were filtered off and all volatiles were removed *in vacuo*. The crude product was recrystallized in *n*-pentane at –30 °C to obtain the product as a colorless solid.

Yield: 6.87 g (10.2 mmol, 51 %).

^{1}H-NMR (300 MHz, CDCl$_3$): δ = 8.06 (s, 2H, *β*-Thi-*H*), 6.98 (s, 4H, arom. Tip-*H*), 2.91 (sept, 2H, $^{3}J_{HH}$ = 6.9 Hz, *p*-^{i}Pr-C*H*), 2.55 (sept, 4H, $^{3}J_{HH}$ = 6.7 Hz, *o*-^{i}Pr-C*H*), 1.28 (d, 12H, $^{3}J_{HH}$ = 6.9 Hz, *p*-^{i}Pr-C*H*$_3$), 1.23 (d, 12H, $^{3}J_{HH}$ = 6.7 Hz, *o*-^{i}Pr-C*H*$_3$), 1.09 (d, 12H, $^{3}J_{HH}$ = 6.7 Hz, *o*-^{i}Pr-C*H*$_3$) ppm.

^{11}B{^{1}H}-NMR (96 MHz, CDCl$_3$): δ = 61.4 ppm.

HRMS (LIFDI) found: m/z = 670.1983 ([M]$^+$); calcd. for [$C_{34}H_{48}B_2Br_2S$]$^+$: 670.2004.

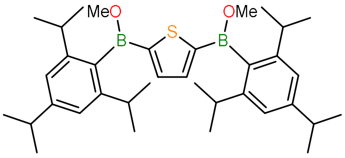

Synthesis of 2,5-bis[methoxy(2,4,6-tri-*iso*-propylphenyl)boryl]thiophene 10: **8** (5.93 g, 14.0 mmol) was dissolved in toluene (30 mL) and a solution of Tip-Li (6.04 g, 28.7 mmol) in toluene (20 mL) was added slowly at room temperature. The mixture was stirred overnight, and the generated solid was filtered off. The residue was washed with toluene several times. Subsequently, the solvent was evaporated *in vacuo*. Then, the crude product **9** was recrystallized out of *n*-hexane. The product was filtered off once again, washed with cold *n*-hexane and dried *in vacuo* to obtain a colorless solid. Then, it was dissolved in DCM (20 mL) and TMS-OMe (2.07 g, 19.9 mmol) was added. The reaction mixture was stirred at room temperature for 1 h. Then, the solvent was removed *in vacuo* and the raw product was recrystallized from a mixture of DCM and methanol. The solid was filtered off, washed with cold methanol (10 mL), and dried *in vacuo*. The product **10** was obtained as colorless needles.

Yield: 4.32 g (7.54 mmol, 54%).

^{1}H-NMR (500 MHz, CDCl$_3$): δ = 7.38 (s, 2H, *β*-Thi-*H*), 6.98 (s, 2H, arom. Tip-*H*), 3.75 (s, 6H, -O-C*H*$_3$), 2.91 (sept, 2H, $^{3}J_{HH}$ = 6.9 Hz, *p*-^{i}Pr-C*H*), 2.59 (sept, 4H, $^{3}J_{HH}$ = 6.9 Hz, *o*-^{i}Pr-C*H*), 1.28 (d, 12H, $^{3}J_{HH}$ = 6.9 Hz, *p*-^{i}Pr-C*H*$_3$), 1.23 (br d, 12H, $^{3}J_{HH}$ = 6.5 Hz, *o*-^{i}Pr-C*H*$_3$), 1.05 (br d, 12H, $^{3}J_{HH}$ = 6.5 Hz, *o*-^{i}Pr-C*H*$_3$) ppm.

^{13}C-NMR (126 MHz, CDCl$_3$): δ = 150.24 (q *C* next to *o*-^{i}Pr), 149.72 (α-Th *C*), 149.41 (q next to *p*-^{i}Pr), 138.84 (β-Th *C*), 133.12 (q Tip *C*-B), 120.33 (arom. Tip *C*-H), 56.03 (-O-*C*H$_3$), 35.24 (*o*-^{i}Pr-*C*-H), 34.42 (*p*-^{i}Pr-*C*-H), 24.71 (*o*-^{i}Pr-*C*H$_3$), 24.19 (*p*-^{i}Pr-*C*H$_3$) ppm.

$^{11}B\{^{1}H\}$-NMR (160 MHz, CDCl$_3$): δ = 44.0 ppm.
HRMS (LIFDI) found: m/z = 573.4080 ([M]$^+$); calcd. for $[C_{36}H_{54}B_2O_2S]^+$: 573.4103.

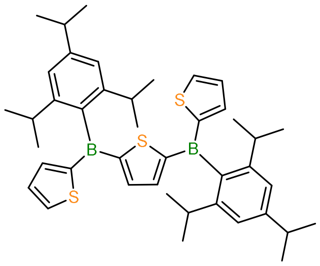

Synthesis of 2,5-bis[thiophen-2-yl(2,4,6-tri-*iso*-propylphenyl)boryl]-thiophene 2 (modified procedure of the literature[9]):

A solution of *in situ* generated 2-lithiumthiophene was synthesized by addition of *n*-BuLi in hexane (4.20 mmol, 2.63 mL) to 2-bromothiophene (652 mg, 4.00 mmol) in hexane (12 mL) and stirring for 3 h. A solution of **10** (1.15 g, 2.00 mmol) in hexane (6 mL) was added dropwise to that mixture. It was stirred overnight. The mixture was worked up aqueously. The raw product was extracted with DCM and washed with brine and water and then dried over $MgSO_4$. It was filtered off and the solvent was removed under reduced pressure. The crude product was recrystallized out of *n*-hexane and a colorless solid was obtained.

Yield: 1.06 g (1.56 mmol, 78 %).
^{1}H-NMR (300 MHz, CDCl$_3$): δ = 7.95 (dd, 2H, $^3J_{HH}$ = 4.7 Hz, $^4J_{HH}$ = 1.0 Hz, Th-*H*), 7.91 (dd, 2H, $^3J_{HH}$ = 3.6 Hz, $^4J_{HH}$ = 0.9 Hz, Th-*H*), 7.82 (s, 2H, inner Th-*H*), 7.31 (dd, 2H, $^3J_{HH}$ = 3.6 Hz, $^3J_{HH}$ = 4.6 Hz, Th-*H*), 6.99 (s, 4H, Tip-*H*), 2.93 (sept, 2H, $^3J_{HH}$ = 6.9 Hz, *p*-^{i}Pr-C*H*), 2.51 (sept, 4H, $^3J_{HH}$ = 6.8 Hz, *o*-^{i}Pr-C*H*), 1.31 (d, 12H, $^3J_{HH}$ = 6.9 Hz, *p*-^{i}Pr-CH_3), 1.04 (d, 12H, $^3J_{HH}$ = 6.8 Hz, *o*-^{i}Pr-CH_3), 1.01 (d, 12H, $^3J_{HH}$ = 6.8 Hz, *o*-^{i}Pr-CH_3) ppm.
$^{11}B\{^{1}H\}$-NMR (96 MHz, CDCl$_3$): δ = 58.0 ppm.

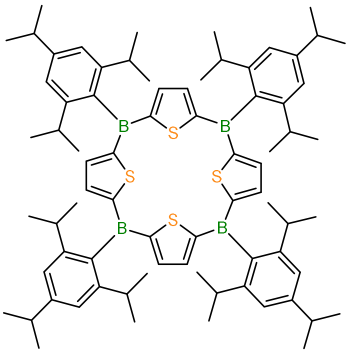

Synthesis of 5,10,15,20-tetrakis(2,4,6-tri-*iso*-propylphenyl)-5,10,15,20-tetrabora-21,22,23,24-tetrathiaporphyrinogen 4 Route I *via* 10 and 2:

A solution of LDA was prepared by addition of *n*-BuLi (1.20 mmol, 750 µL) in hexane to a solution of di-*iso*-propylamine (121 mg, 1.20 mmol) in THF (1 mL) at −30 °C. The mixture was stirred for 30 min. This solution was added to a mixture of **2** (328 mg, 480 µmol) in THF (5 mL) at −78 °C. The solution was stirred for 4 h maintaining the temperature. The green suspension was diluted with THF (20 mL). A cold (−78 °C) solution of **10** (275 mg, 480 µmol) in THF (25 mL) was added dropwise over the course of 2 h at −78 °C. It was stirred for 3 d, while the cooling bath was allowed to warm up to room temperature. Afterwards, the solvent was removed *in vacuo* and the residue was redissolved in DCM (10 mL) and chlorotrimethylsilane (120 mg, 1.10 mmol) was added. It was stirred for 1 h. Then, the mixture was worked up aqueously and the product was extracted with DCM, washed with brine and water, dried over Na_2SO_4, and filtered off. The solvent was removed under reduced pressure. The crude product was purified by column chromatography (silica, 3% DCM in PE). Subsequently, the solid product was recrystallized out of ethylacetate to yield yellow needles.

Synthesis of 4 Route II *via* 1 and 7:

A solution of LDA was prepared by addition of *n*-BuLi (1.20 mmol, 750 µL) in hexane to a solution of di-*iso*-propylamine (121 mg, 1.20 mmol) in THF (1 mL) at −30 °C. The mixture was stirred for 30 min. This solution was added to a mixture of **1** (190 mg, 500 µmol) in THF (5 mL) at −78 °C. The solution was stirred for 4 h maintaining the temperature. The purple solution was diluted with THF (20 mL). A cold (−78 °C) solution of **7** (434 mg, 500 µmol) in THF (25 mL) was added dropwise over the course of 1 h at −78 °C. It was stirred for 3 d, while the cooling bath was allowed to warm up to room temperature. Afterwards, the solvent was removed *in vacuo* and the residue was redissolved in DCM (10 mL) and chlorotrimethylsilane (163 mg, 1.50 mmol) was

added. It was stirred for 1 h. Then, the mixture was worked up aqueously and the product was extracted with DCM, washed with brine and water, dried over Na_2SO_4, and filtered off. The solvent was removed under reduced pressure. The crude product was purified by column chromatography (silica, 3% DCM in PE). Subsequently, the solid product was recrystallized out of ethylacetate to yield yellow needles.

Yield: **Route I**: 347 mg (293 µmol, 61 %); **Route II**: 157 mg (132 µmol, 27 %).
^{1}H-NMR (500 MHz, $CDCl_3$): δ = 7.63 (s, 8H, *β*-Thi-*H*), 7.03 (s, 8H, arom. Tip-*H*), 2.95 (sept., 4H, $^3J_{HH}$ = 6.9 Hz, ^{i}Pr-C*H*), 2.63 (sept., 8H, $^3J_{HH}$ = 6.7 Hz, ^{i}Pr-C*H*), 1.32 (d, 24H, $^3J_{HH}$ = 6.9 Hz, *p*-^{i}Pr-CH_3), 1.05 (d, 48H, $^3J_{HH}$ = 6.8 Hz, *o*-^{i}Pr-CH_3) ppm.
^{13}C-NMR (126 MHz, $CDCl_3$): δ = 157.36 (*α*-Th *C*), 150.09 (q *C* next *o*-^{i}Pr), 149.14 (q *C* next to *p*-^{i}Pr), 141.90 (*β*-Th *C*), 137.62 (q Tip-*C*-B), 120.35 (arom. Tip *C*-H), 35.89 (*o*-^{i}Pr-*C*H), 34.42 (*p*-^{i}Pr-*C*H), 24.39 (*o*-^{i}Pr-*C*H_3), 24.23 (*p*-^{i}Pr-*C*H_3) ppm.
^{11}B{^{1}H}-NMR (96 MHz, $CDCl_3$): 57.4 ppm
HRMS (LIFDI) found: m/z = 1184.7058 ([M]$^+$); calcd. for $[C_{76}H_{100}B_4S_4]^+$: 1184.7075.
Elemental Analysis: Calcd (found) [%] for $C_{76}H_{100}B_4S_4$: C 77.03 (77.11), H 8.51 (8.55), S 10.82 (10.70).
Absorption $\lambda_{abs,max}$ = 340 nm in THF.
Fluorescence $\lambda_{em,max}$ = 511 nm (λ_{ex} = 320 nm) in THF (Φ_f = 0.01).

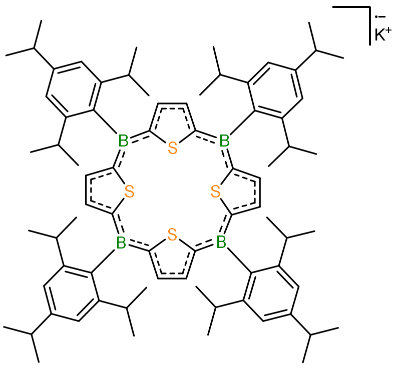

Synthesis of K[4]
A solution of **4** (59.3 mg, 50.0 µmol) in THF (0.5 mL) and a solution of **K_2[4]** (63.2, 0.05 mmol) in THF (0.5 mL) were mixed. The red solution was diluted with *n*-pentane (5 mL) and crystallized at −30 °C. It was filtered off and the product was dried *in vacuo* and obtained as a red crystalline solid.

Yield: 58.8 mg (35.5 µmol, 71 %). Crystallized as [K(THF)$_6$][4]

Absorption $\lambda_{abs,max1}$ = 365 nm, $\lambda_{abs,max2}$ = 455 nm, $\lambda_{abs,max3}$ = 479 nm, $\lambda_{abs,shoulder}$ = 525 nm, $\lambda_{abs,shoulder}$ = 555 nm, $\lambda_{abs,shoulder}$ = 910 nm, $\lambda_{abs,max4}$ = 1035 nm, $\lambda_{abs,max5}$ = 1210 nm in THF.

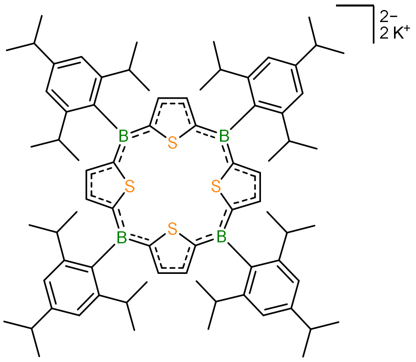

Synthesis of K$_2$[4]

A solution of **4** (119 mg, 100 µmol) in toluene (2.5 mL) was added to a suspension of KC_8 (33.8 mg, 250 µmol) in toluene (2.5 mL) at room temperature. The mixture was stirred for 3 min and then filtered off. The product was precipitated out of the green solution at −30 °C overnight. The dark turquoise solid was filtered off and dried *in vacuo*.

Alternative: The reaction can be carried out in THF analogously with the benefit of yielding dark green crystals at the crystallization-step, which can be analyzed by means of X-ray diffraction. The coordinating THF molecules are difficult to remove though, and therefore the procedure in non-polar solvents grants a solvent-free product.

Yield: 88.4 mg (70.0 µmol, 70 %); NMR-Yield: quantitative

^{1}H-NMR (500 MHz, C_6D_6): δ = 9.34 (s, 8H, *β*-Thi-*H*), 7.59 (s, 8H, arom. Tip-*H*), 3.30-3.19 (-, 12H, ^{i}Pr-C*H*), 1.60 (d, 24H, $^3J_{HH}$ = 6.9 Hz, *p*-^{i}Pr-CH_3), 1.21 (d, 48H, $^3J_{HH}$ = 6.9 Hz, *o*-^{i}Pr-CH_3) ppm.

^{13}C-NMR (126 MHz, $CDCl_3$): δ = 151.78 (q *C* next to *o*-^{i}Pr), 150.56 (q *C* next *p*-^{i}Pr), 147.98 (*α*-Th *C*), 144.01 (q Tip-*C*-B), 137.98 (*β*-Th *C*), 120.47 (arom. Tip *C*-H), 36.03 (*o*-^{i}Pr-CH), 35.27 (*p*-^{i}Pr-*C*H), 25.02 (*o*-^{i}Pr-*C*H$_3$), 24.85 (*p*-^{i}Pr-*C*H$_3$) ppm

^{11}B{^{1}H}-NMR (96 MHz, $CDCl_3$): 38.2 ppm

Absorption $\lambda_{abs,max1}$ = 385 nm, $\lambda_{abs,max2}$ = 405 nm, $\lambda_{abs,max3}$ = 766 nm, $\lambda_{abs,max4}$ =

852 nm in THF.

Fluorescence $\lambda_{em,max}$ = 899 nm (λ_{ex} = 405 nm) in THF (Φ_f = 0.01).

NMR Spectra

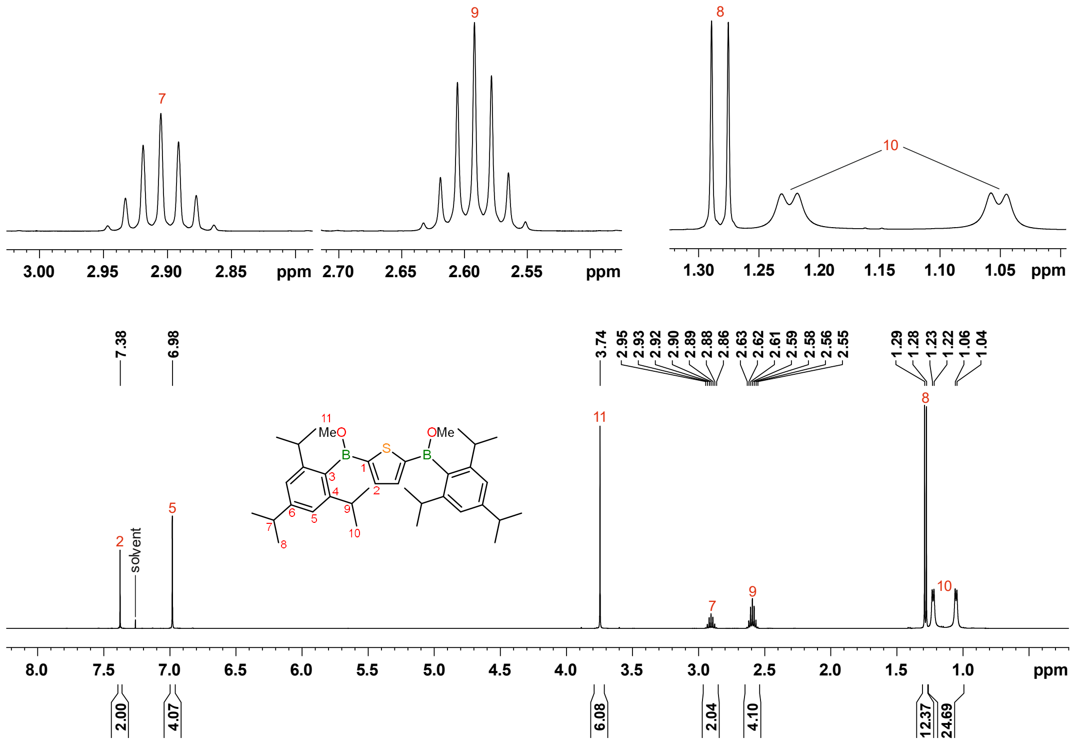

Figure S1: ^{1}H-NMR spectrum of **10** (500 MHz, in $CDCl_3$).

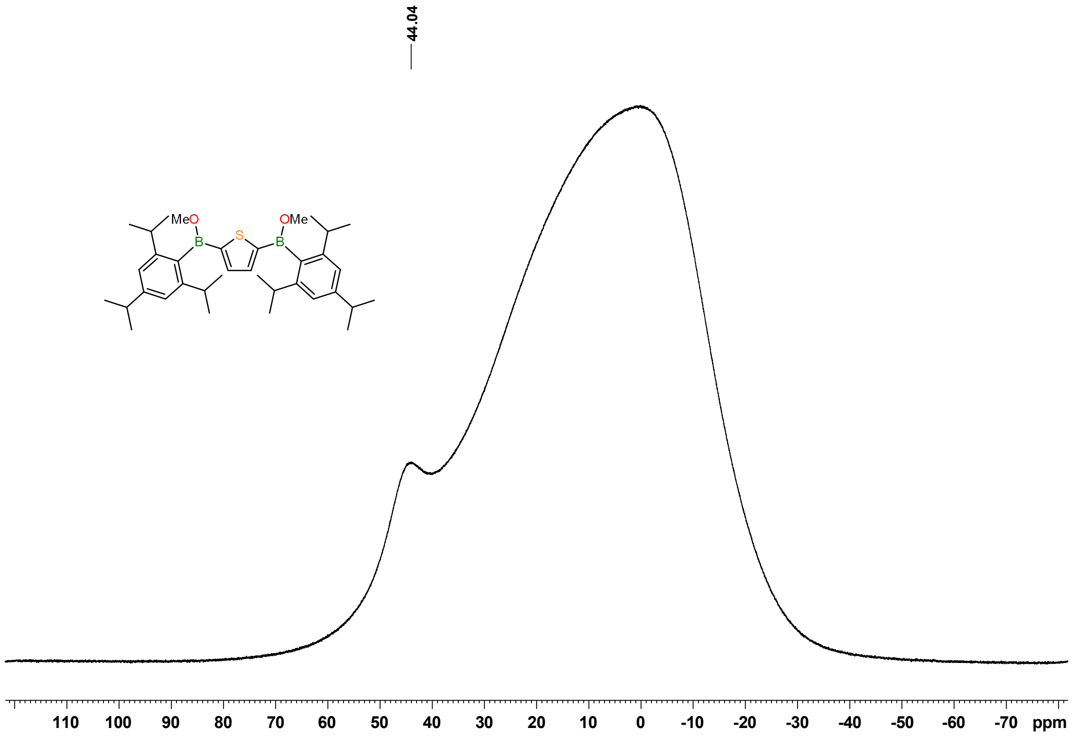

Figure S2: $^{11}B\{^{1}H\}$-NMR spectrum of **10** (160 MHz, in $CDCl_3$).

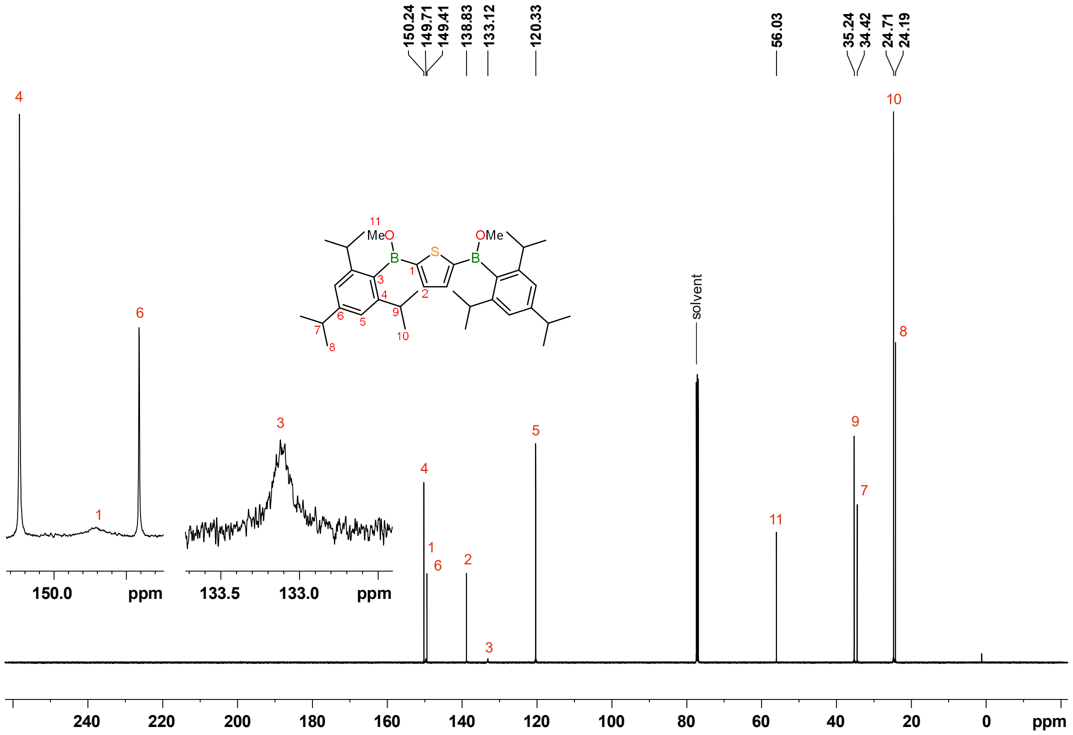

Figure S3: ^{13}C-NMR spectrum of **10** (126 MHz, in $CDCl_3$).

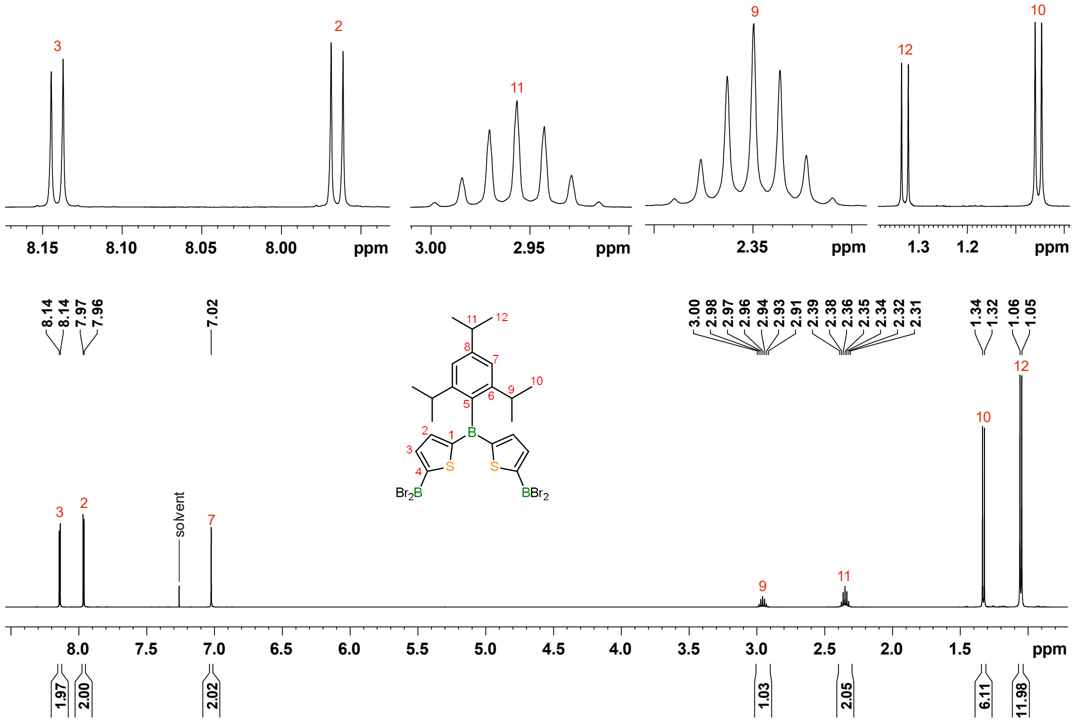

Figure S4: ^{1}H-NMR spectrum of **6** (500 MHz, in $CDCl_3$).

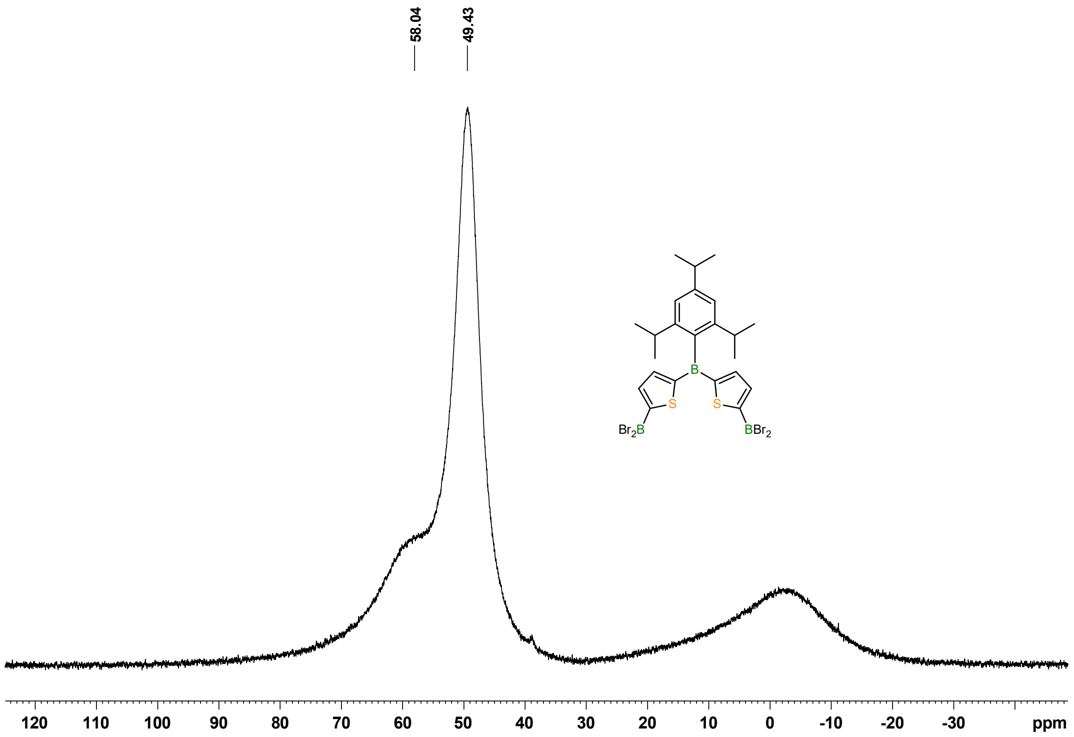

Figure S5: $^{11}B\{^1H\}$-NMR spectrum of **6** (160 MHz, in $CDCl_3$).

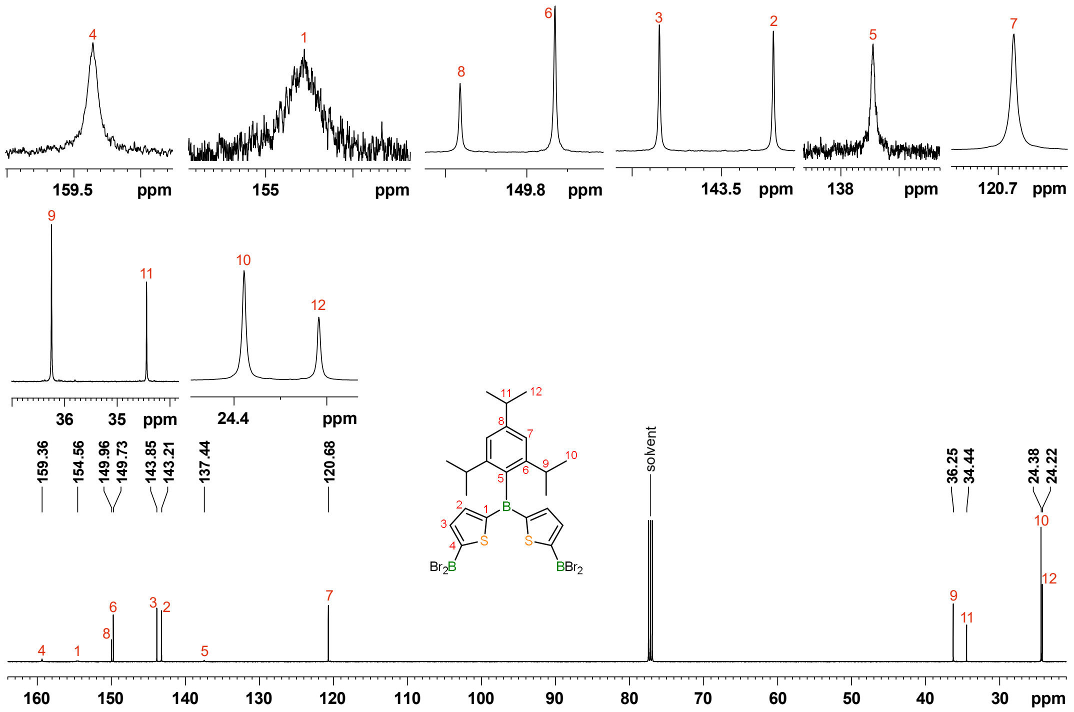

Figure S6: ^{13}C-NMR spectrum of **6** (126 MHz, in $CDCl_3$).

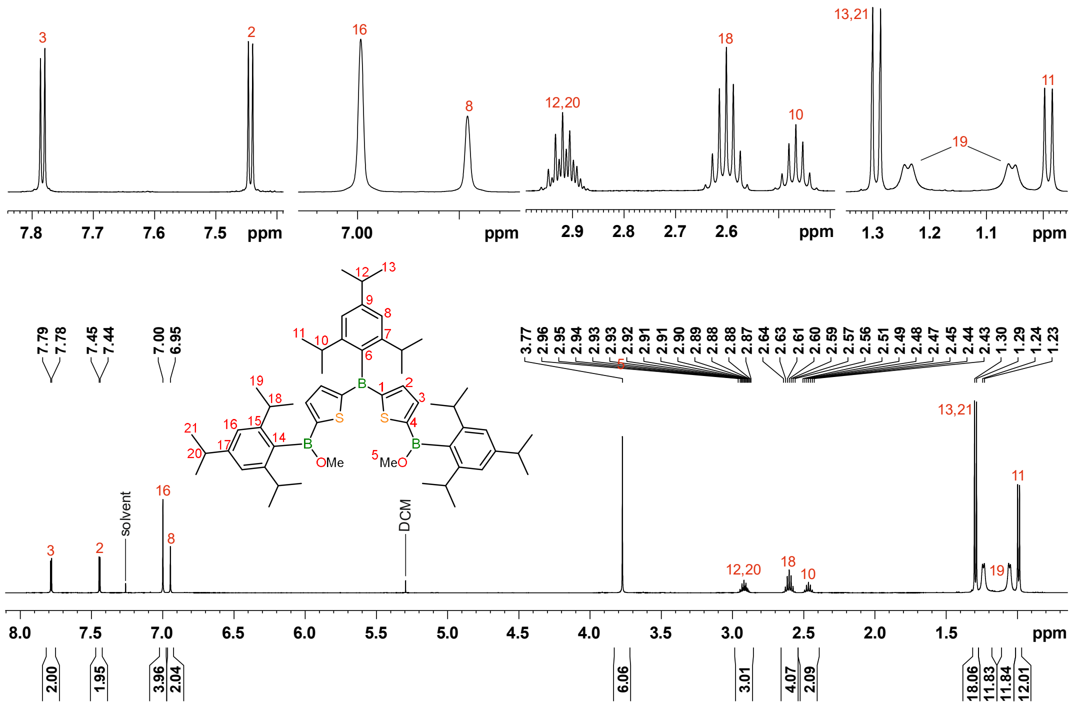

Figure S7: ^{1}H-NMR spectrum of **7** (500 MHz, in $CDCl_3$).

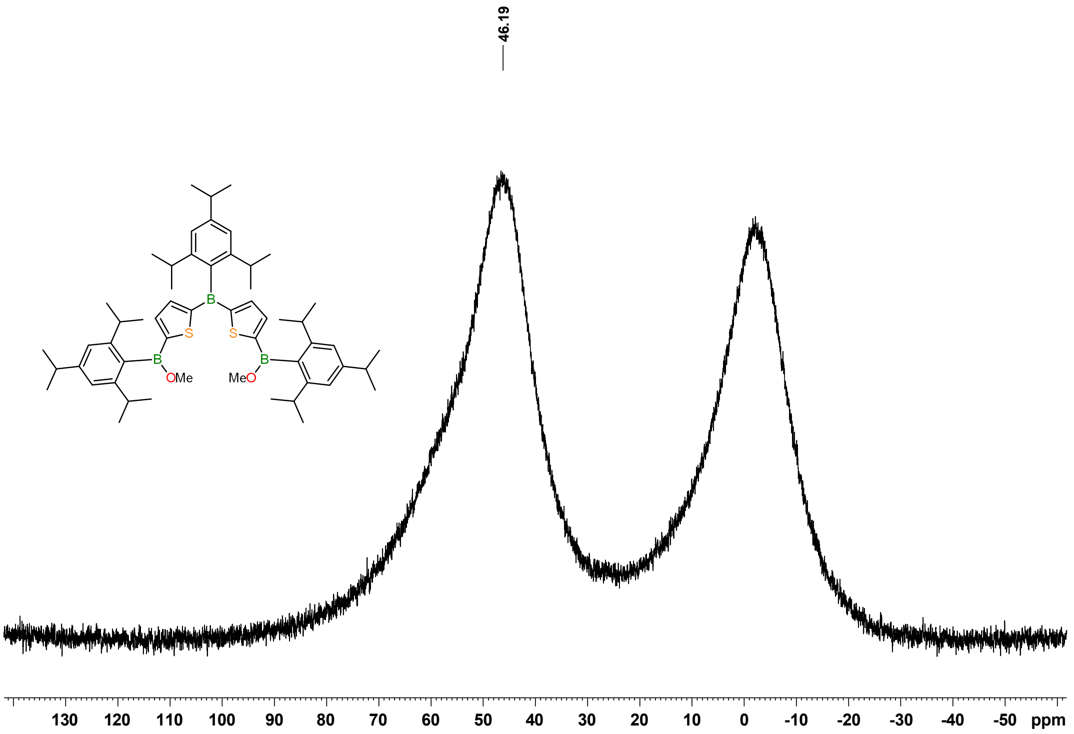

Figure S8: ^{11}B{^{1}H}-NMR spectrum of **7** (160 MHz, in $CDCl_3$).

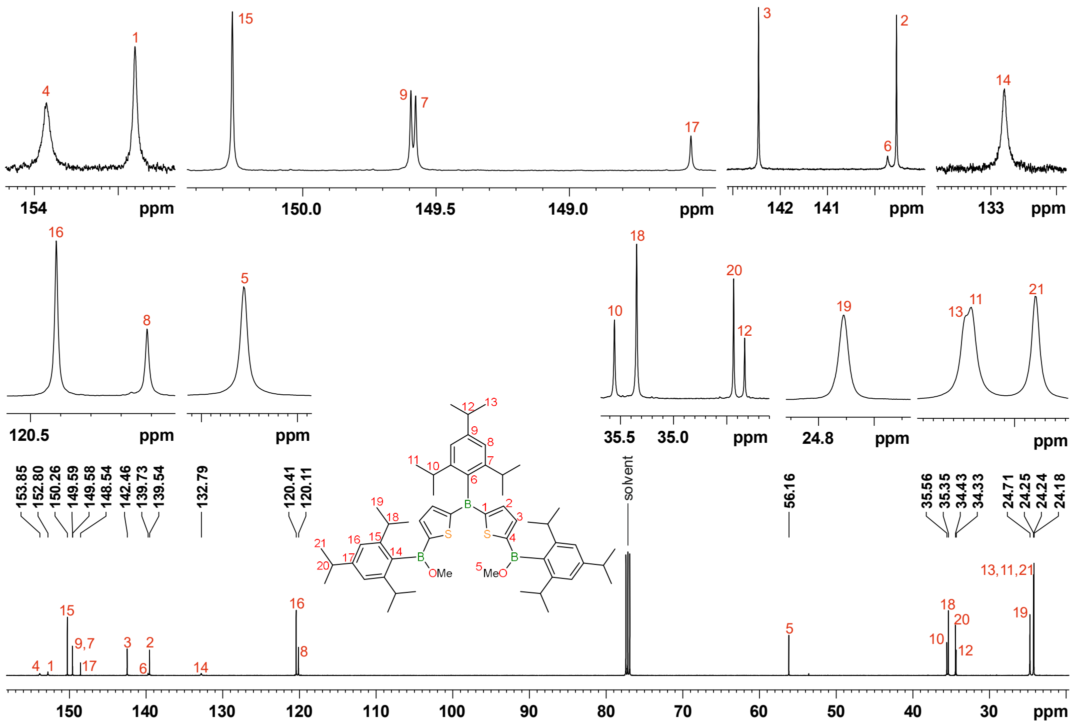

Figure S9: ^{13}C-NMR spectrum of **7** (126 MHz, in $CDCl_3$).

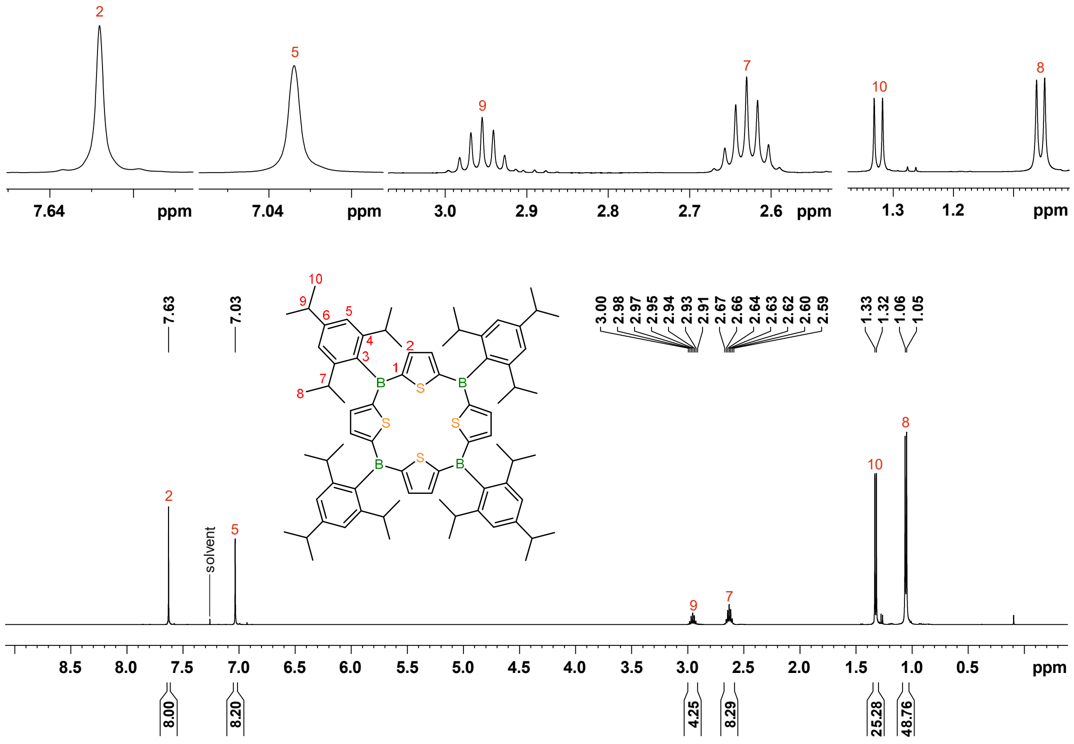

Figure S10: 1H-NMR spectrum of **4** (500 MHz, in $CDCl_3$).

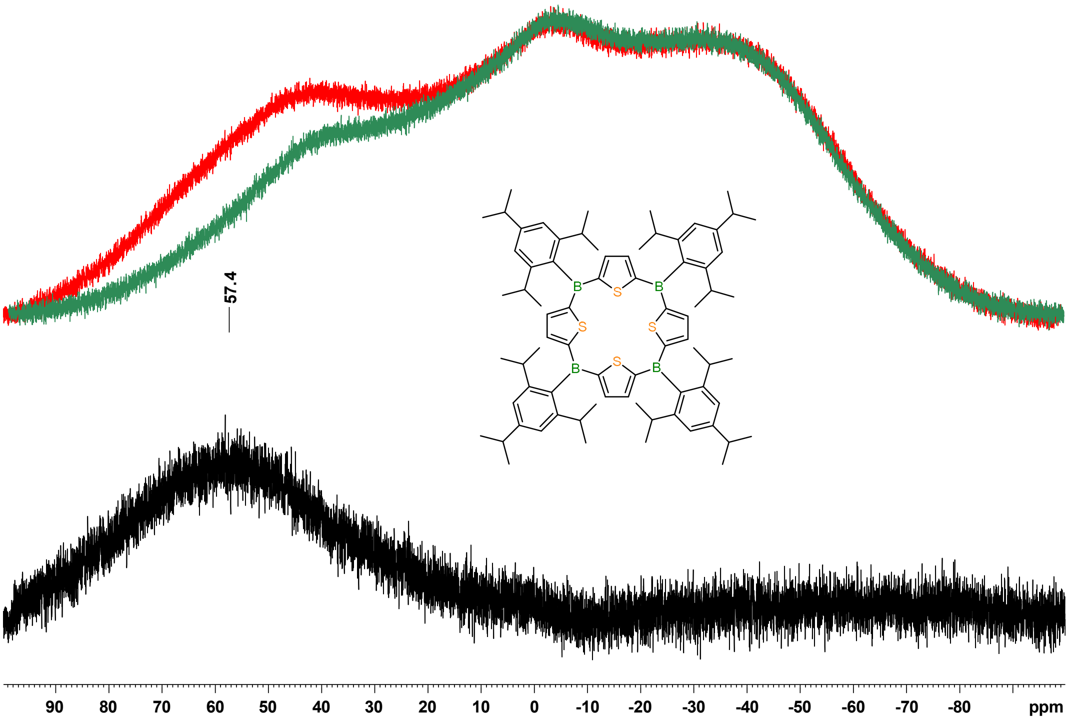

Figure S11: $^{11}B\{^{1}H\}$-NMR spectrum of **4** (96 MHz, in $CDCl_3$). Black: Difference spectrum; red: sample; green: blank.

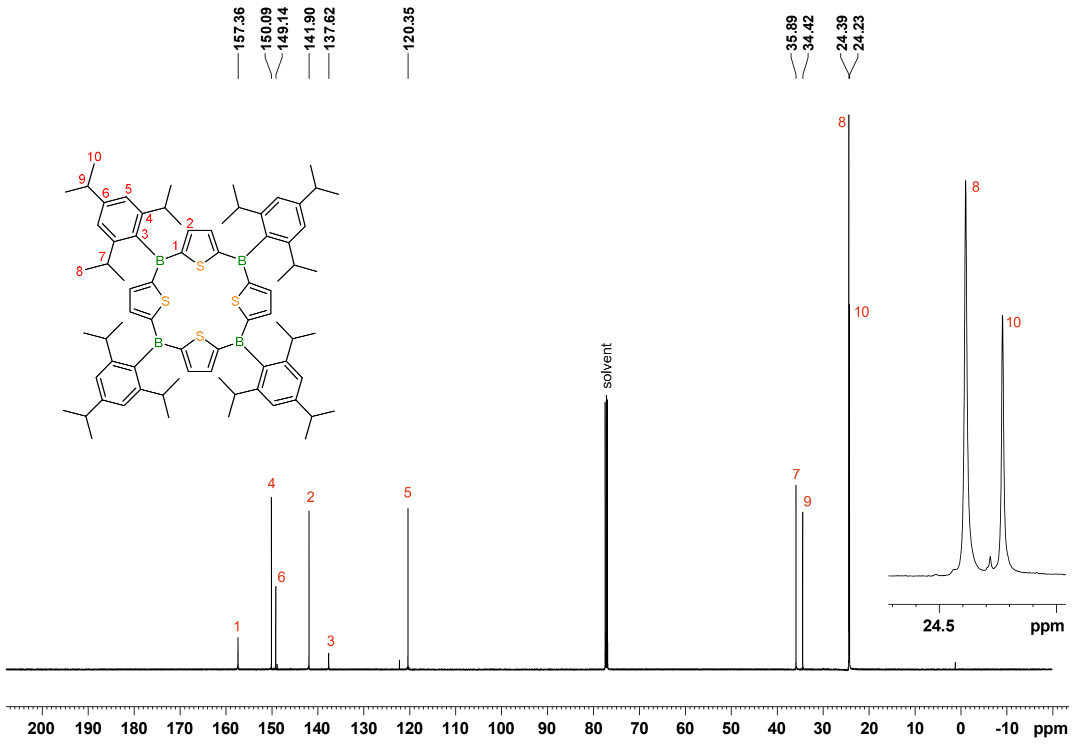

Figure S12: ^{13}C-NMR spectrum of **4** (126 MHz, in $CDCl_3$).

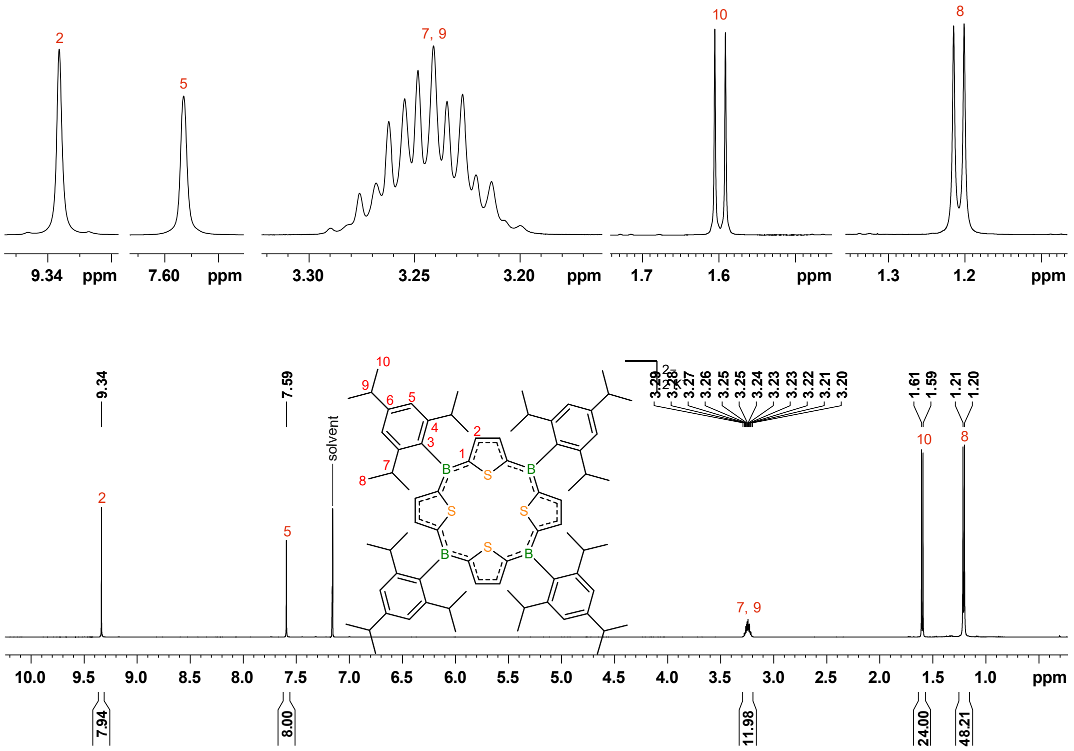

Figure S13: ^{1}H-NMR spectrum of **K_2[4]** (500 MHz, in C_6D_6).

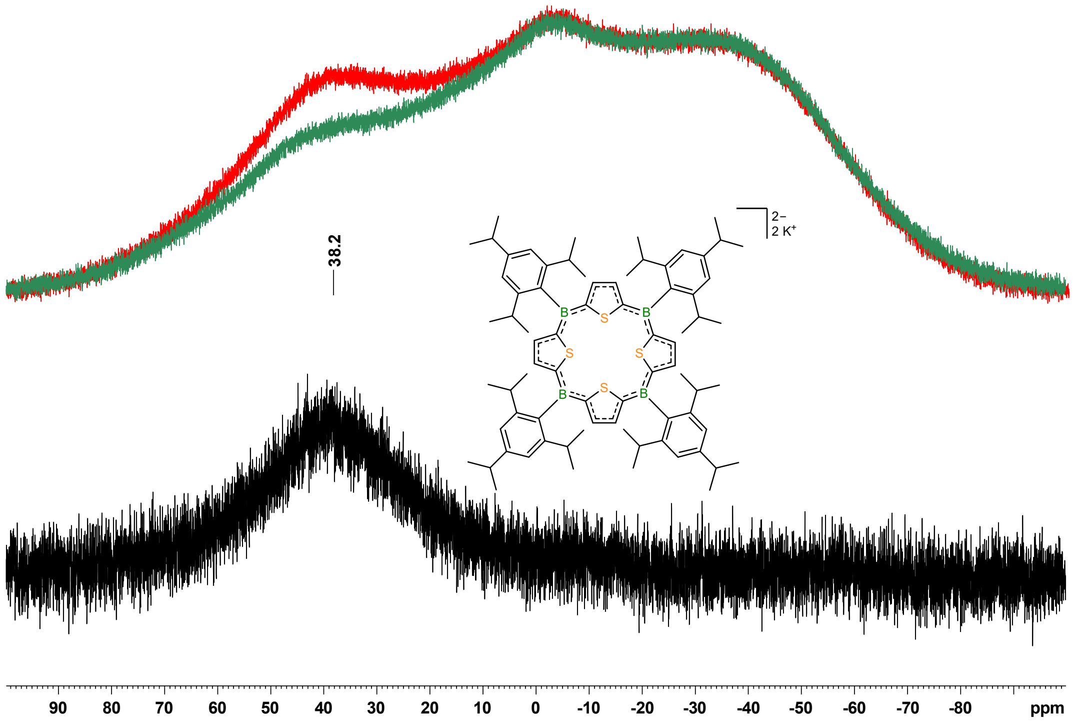

Figure S14: $^{11}B\{^{1}H\}$-NMR spectrum of **K_2[4]** (96 MHz, in C_6D_6). Black: Difference spectrum; red: sample; green: blank.

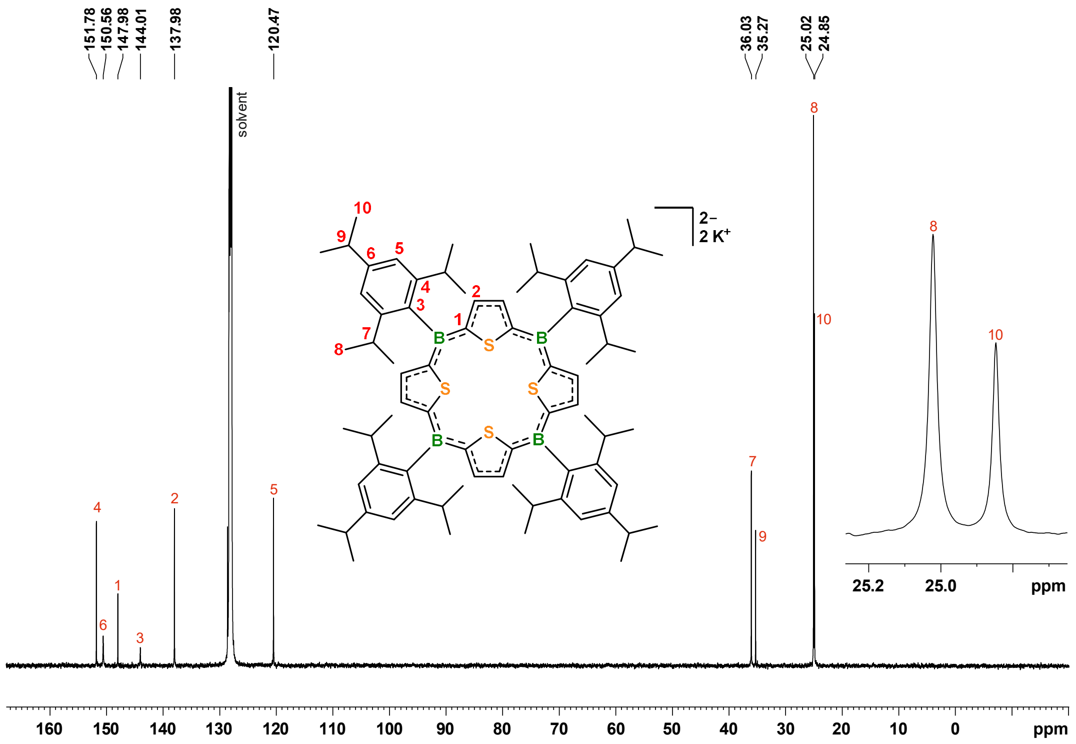

Figure S15: ^{13}C-NMR spectrum of **K₂[4]** (126 MHz, in C_6D_6).

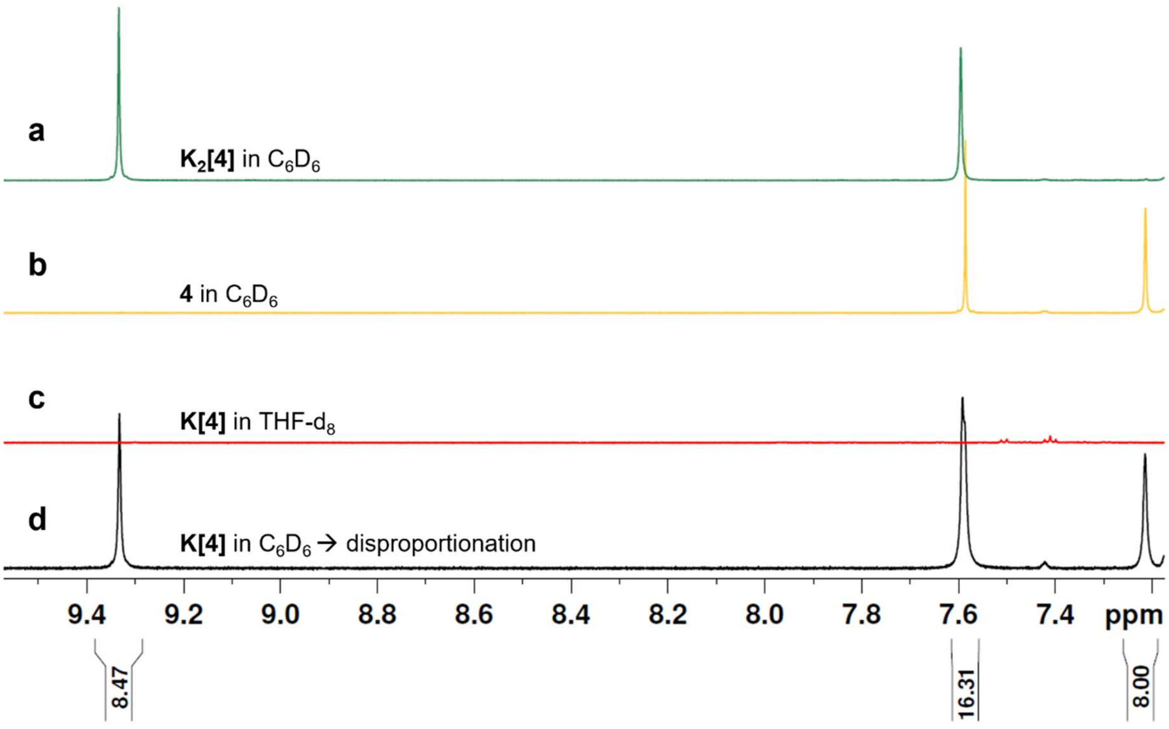

Figure S16: ^{1}H-NMR monitoring of the disproportionation-reaction of $4^{•-}$ in the non-polar solvent C_6D_6. **a**: salt **K₂[4]** of the dianionic species in C_6D_6. **b**: neutral macrocycle **4** in C_6D_6. **c**: persistent radical species **K[4]** in THF-d_8. **d**: disproportionated radical in C_6D_6. The solvent can be exchanged several times to repeat the comproportionation and the disproportionation respectively.

High Resolution Mass Spectra

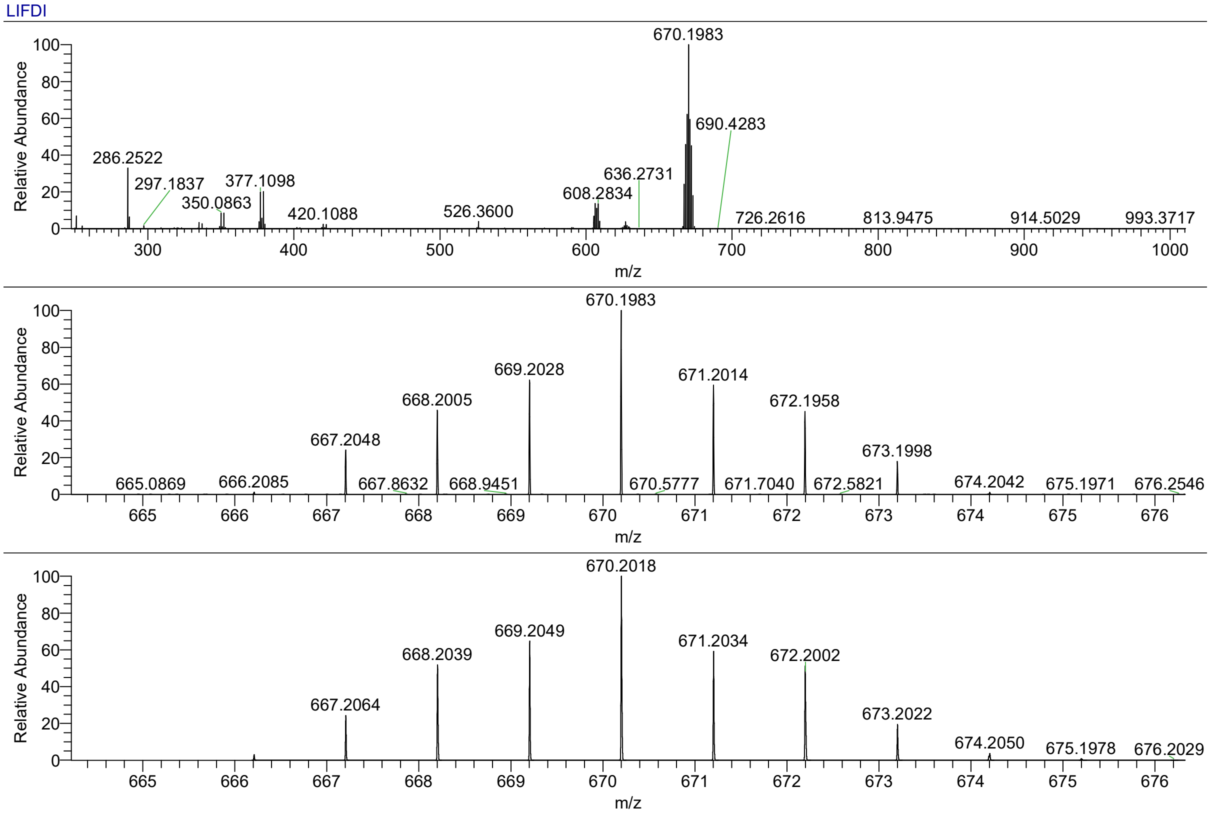

Figure S17: HRMS spectrum (LIFDI) of **9**. The bottom spectrum represents the simulated isotope pattern.

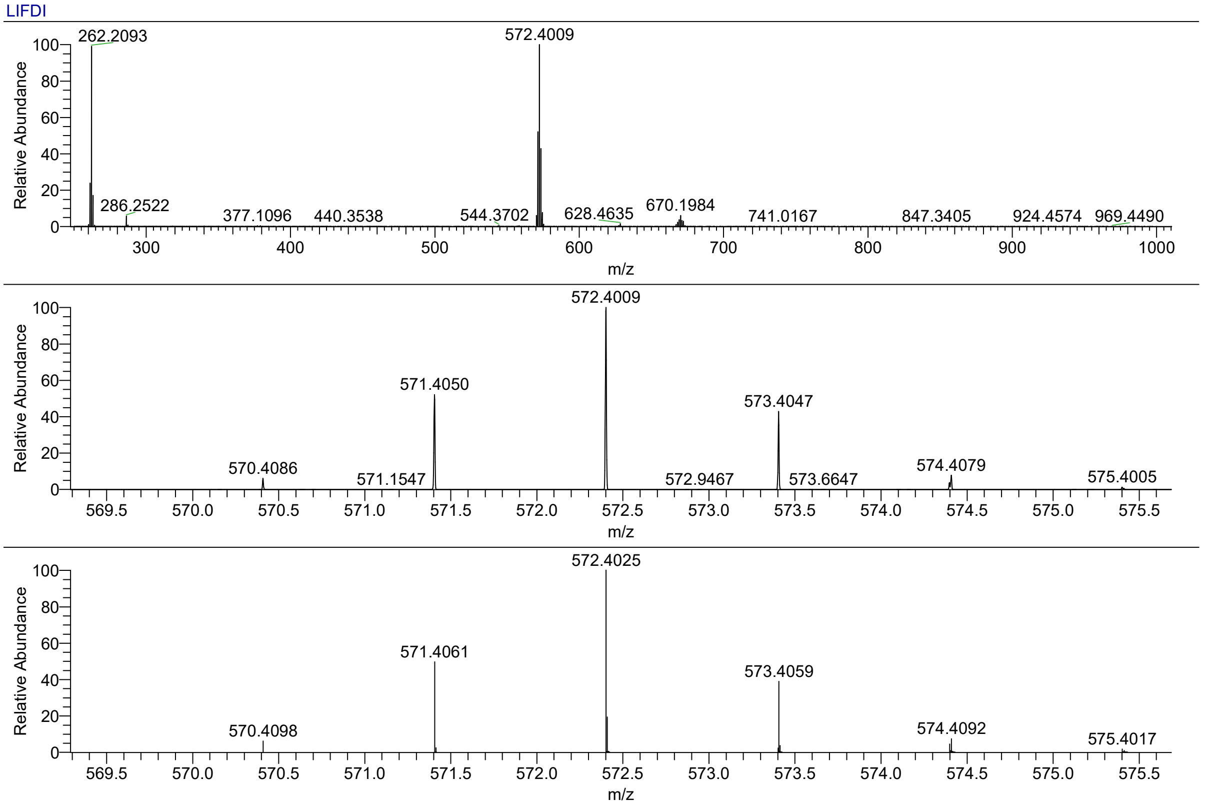

Figure S18: HRMS spectrum (LIFDI) of **10**. The bottom spectrum represents the simulated isotope pattern.

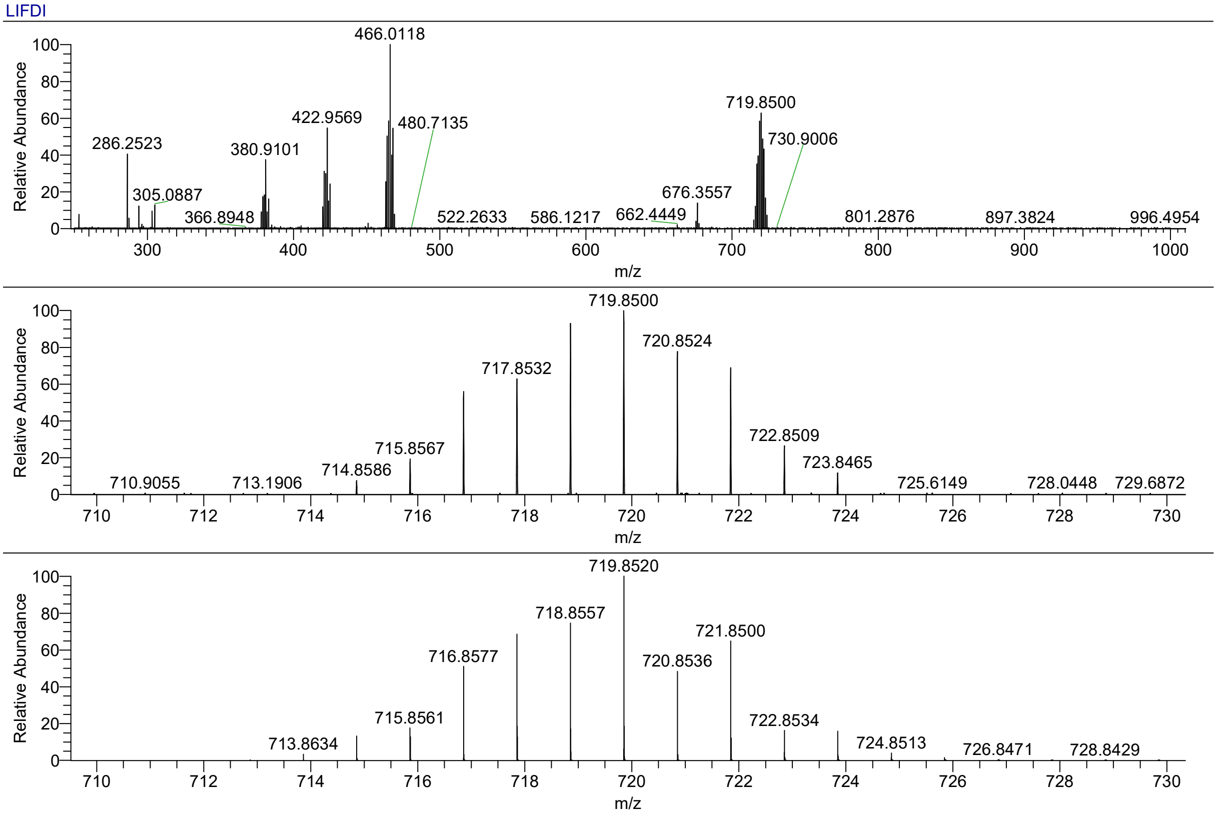

Figure S19: HRMS spectrum (LIFDI) of **6**. The bottom spectrum represents the simulated isotope pattern.

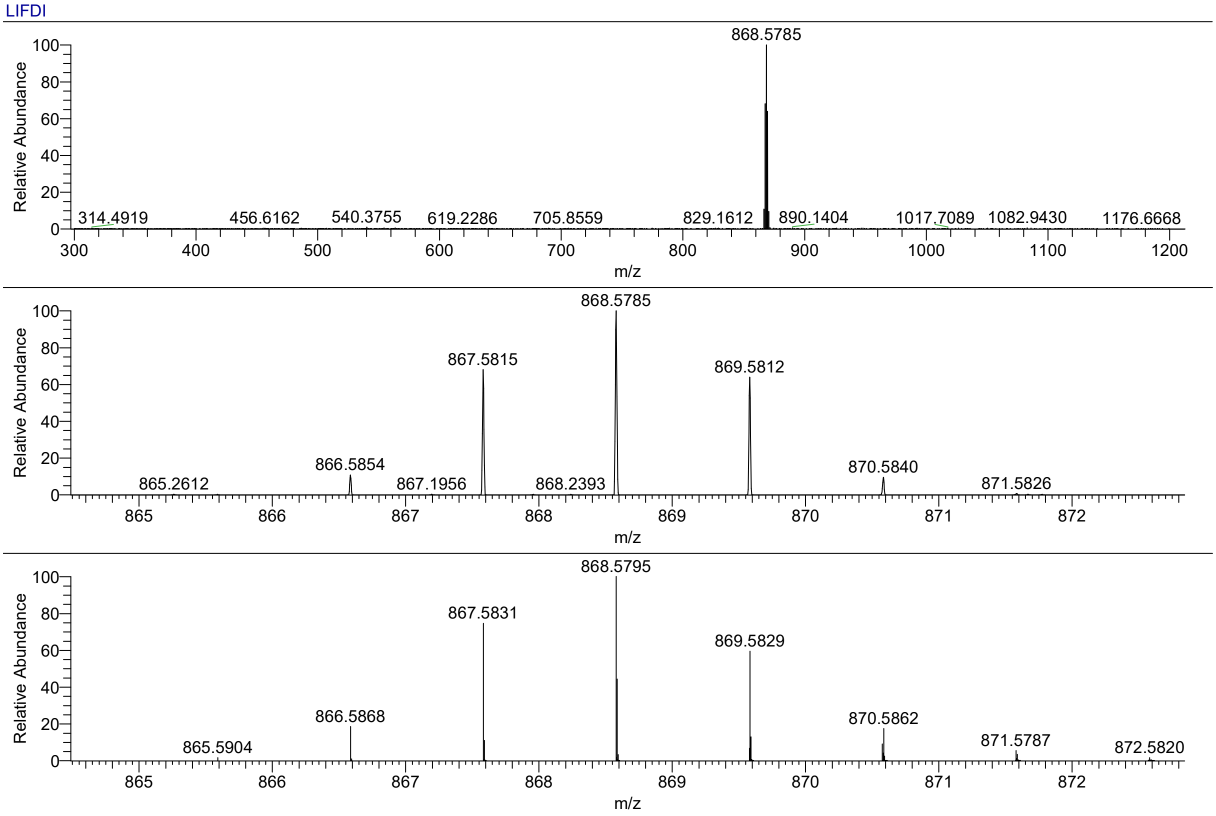

Figure S20: HRMS spectrum (LIFDI) of **7**. The bottom spectrum represents the simulated isotope pattern.

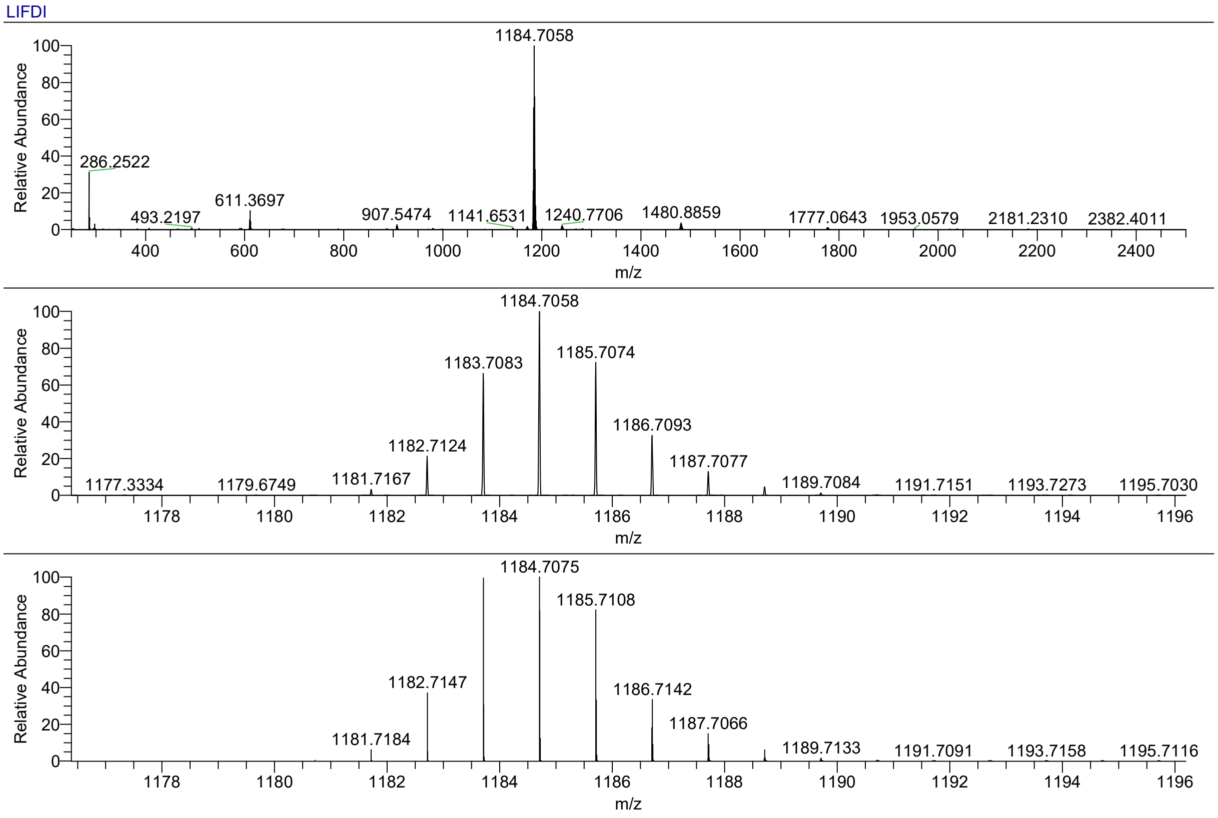

Figure S21: HRMS spectrum (LIFDI) of **4**. The bottom spectrum represents the simulated isotope pattern.

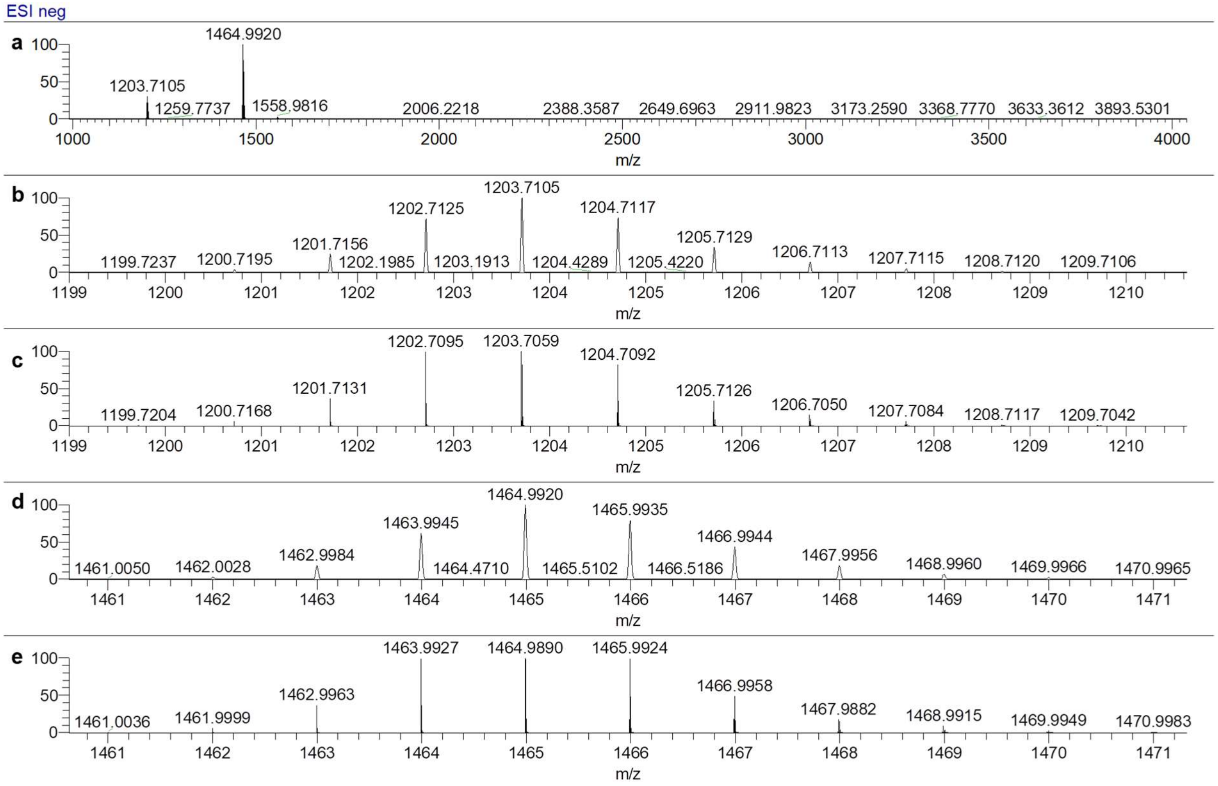

Figure S22: **a**, HRMS spectrum (ESI neg) of the reaction mixture of **4** with TBAF. **b**, found mass of **[4−F]**$^-$. **c**, simulated isotope pattern of **[4−F]**$^-$. **d**, found mass of **[(NBu$_4$)(4−F$_2$)]**$^-$. **e**, simulated isotope pattern of **[(NBu$_4$)(4−F$_2$)]**$^-$.

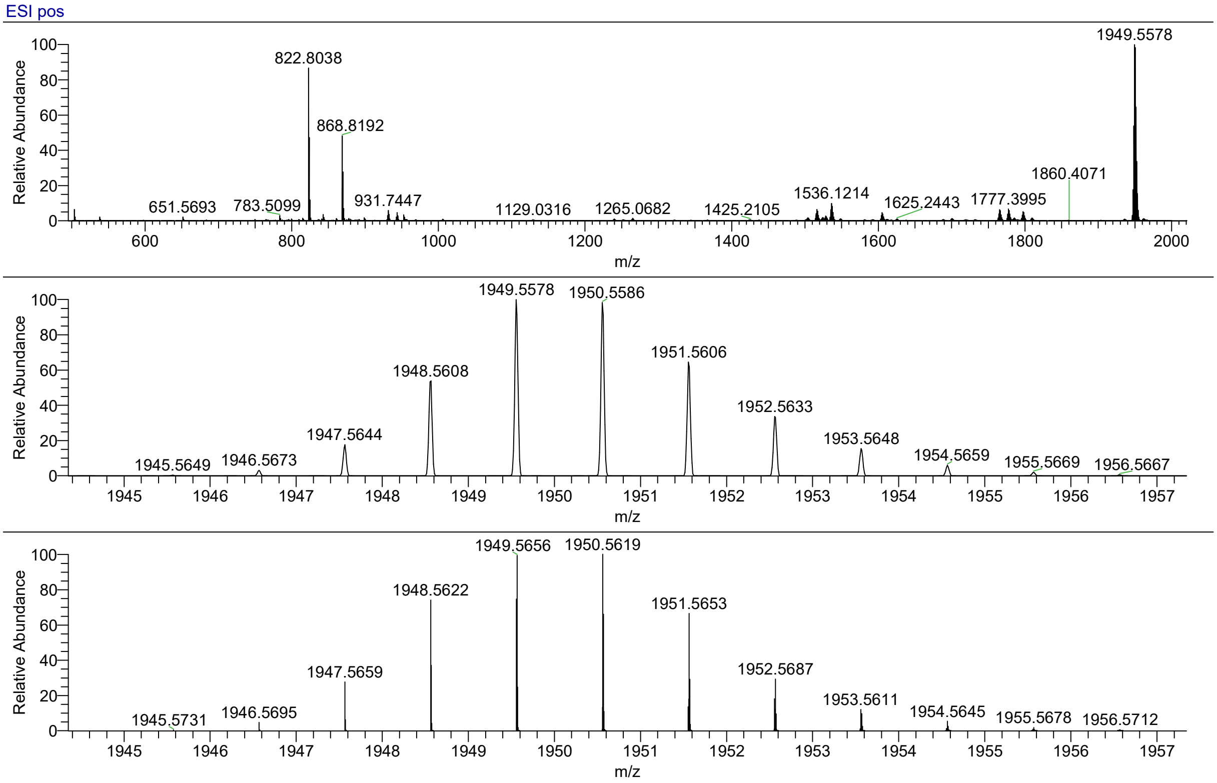

Figure S23: HRMS spectrum (ESI pos) of the reaction mixture of **4** with TBAF. Middle: found mass of **[(NBu$_4$)$_3$(4−F$_2$)]$^+$**. Bottom: simulated isotope pattern of **[(NBu$_4$)$_3$(4−F$_2$)]$^+$**.

UV-vis and Fluorescence Spectra

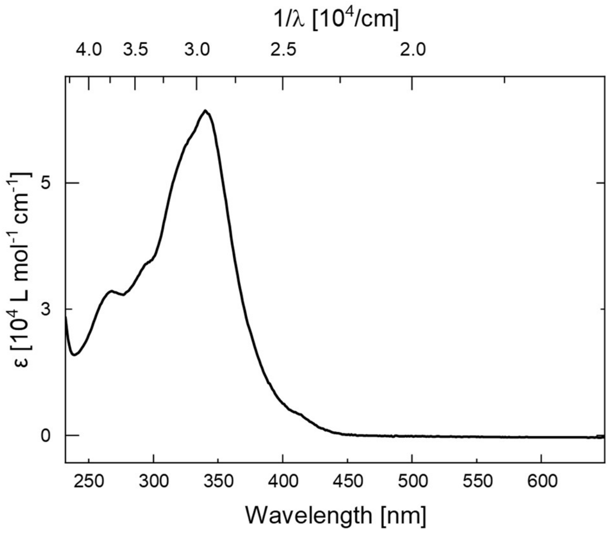

Figure S24: Absorption spectrum of **4** in THF. ε = 64370 L mol^{-1} cm^{-1} for the lowest energy band at 340 nm.

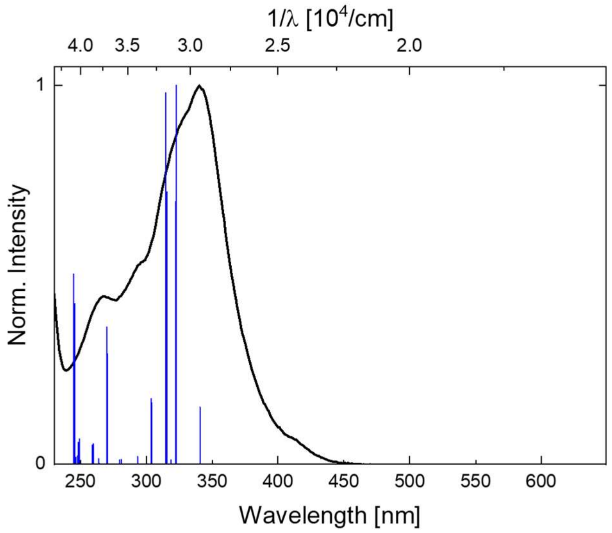

Figure S25: Absorption spectrum of **4** alongside its calculated vertical excitations as a line spectrum (ω_TB97X-D3/def2-SVP, CPCM(THF)).

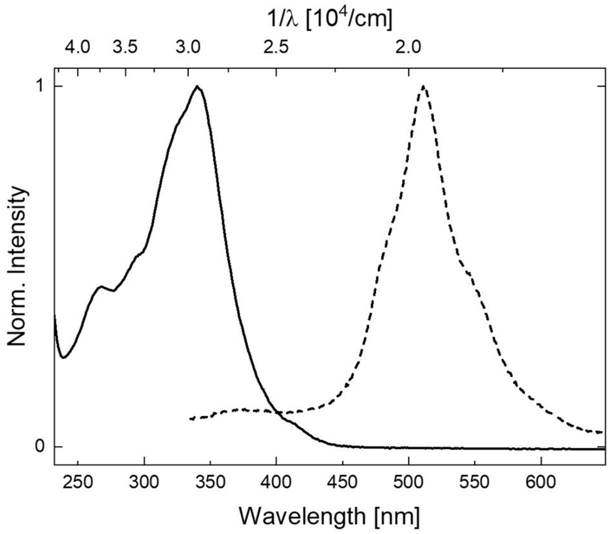

Figure S26: Normalized absorption (solid line) and emission (dashed line) spectra of **4** in THF. $\lambda_{excitation}$ = 320 nm.

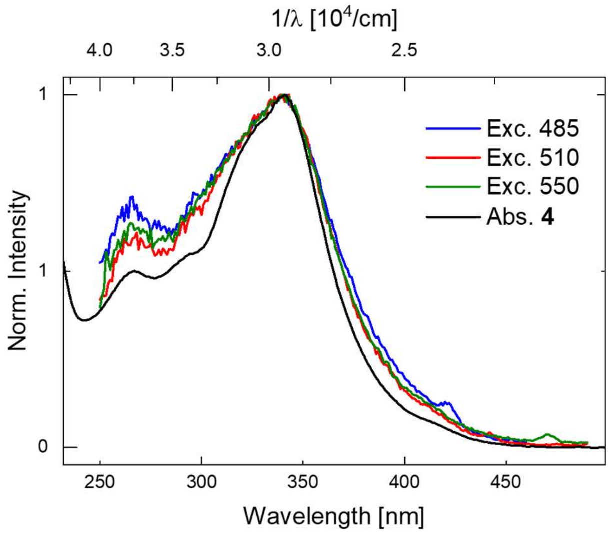

Figure S27: Normalized absorption (black) and associated excitation-spectra of **4** at different emission wavelengths.

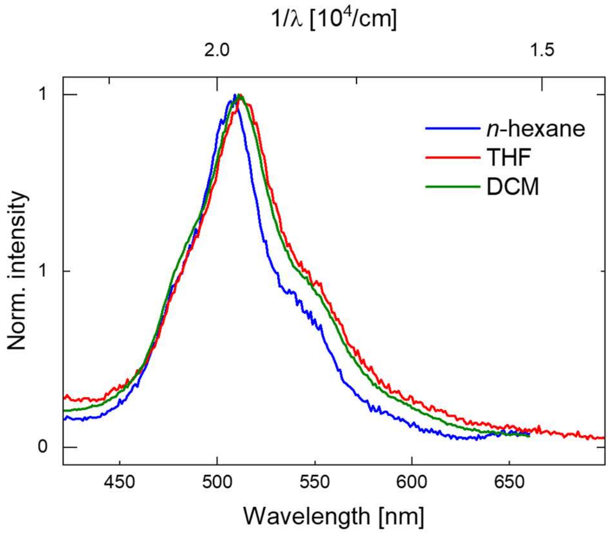

Figure S28: Normalized emission of **4** in different solvents.

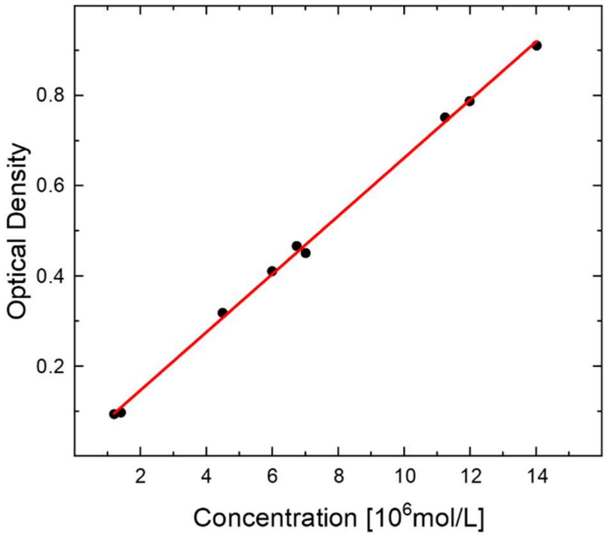

Figure S29: Linear fit of several measurements to determine extinction coefficient of **4**. The optical density of the lowest energy band (340 nm) was considered for the calculation (R^2 = 0.99852).

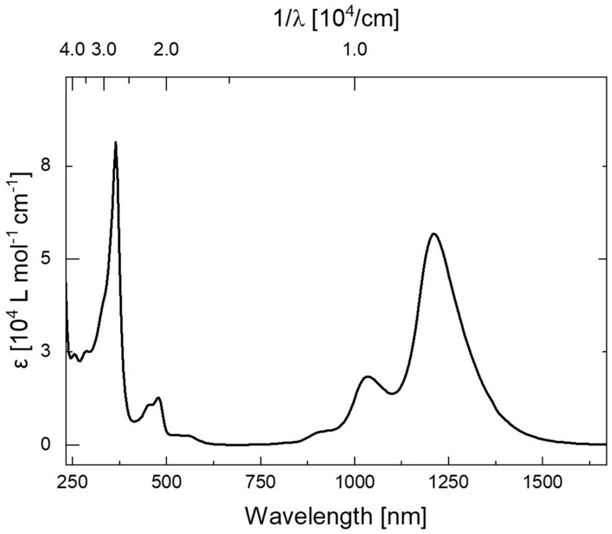

Figure S30: Absorption spectrum of **K[4]** in THF. ε = 81432 L mol^{-1} cm^{-1} for absorption band with the highest optical density at 365 nm.

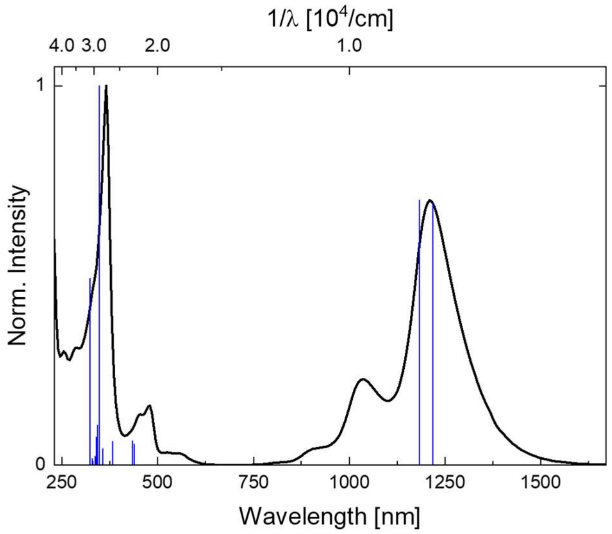

Figure S31: Absorption spectrum of **K[4]** alongside the calculated vertical excitations for $\mathbf{4}^{\bullet -}$ as a line spectrum (UωTB97X-D3/def2-SVP, CPCM(THF)). Calculation without cation.

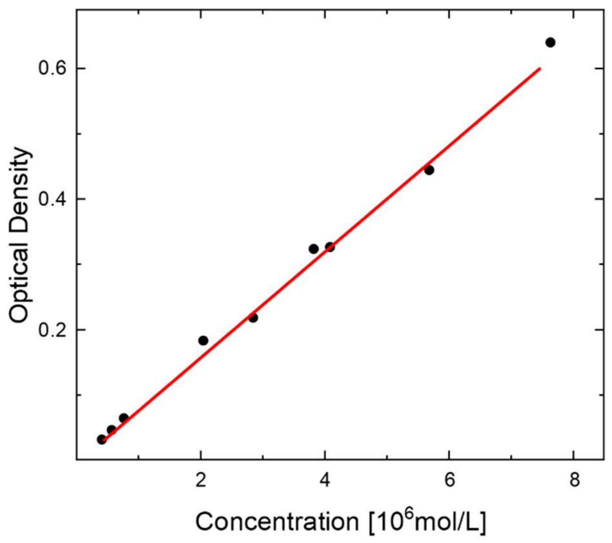

Figure S32: Linear fit of several measurements to determine extinction coefficient of **K[4]**. The optical density of the absorption band with the highest value (366 nm) was considered for the calculation (R^2 = 0.99605).

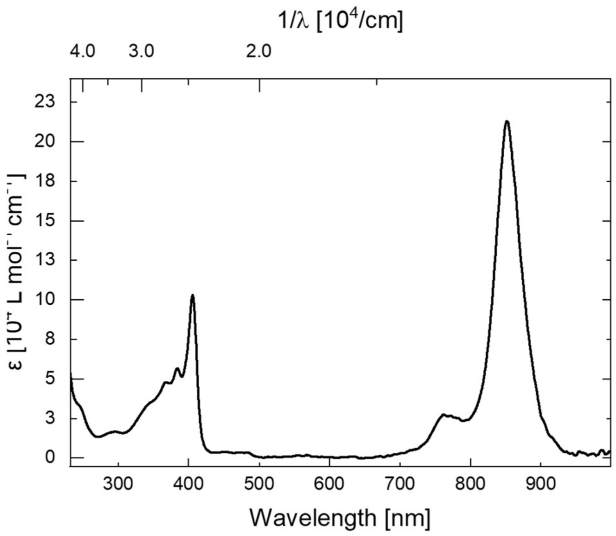

Figure S33: Absorption spectrum of **K_2[4]** in THF. ε = 213120 L mol^{-1} cm^{-1} for the lowest energy band at 852 nm.

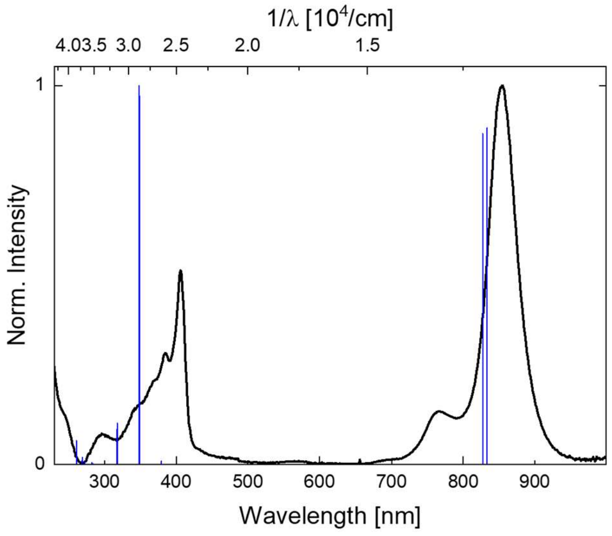

Figure S34: Absorption spectrum of **K_2[4]** alongside the calculated vertical excitations of **4**$^{2-}$ as a line spectrum (ω_TB97X-D3/def2-SVP, CPCM(THF)). Calculation without cation.

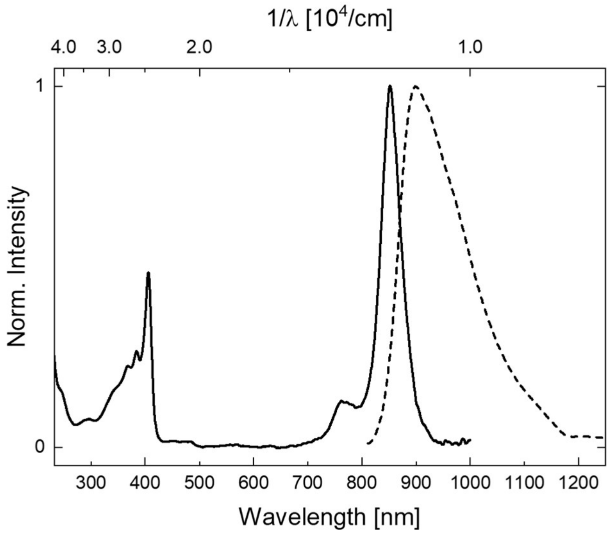

Figure S35: Normalized absorption (solid line) and emission (dashed line) spectra of **K_2[4]** in THF.
$\lambda_{excitation}$ = 405 nm.

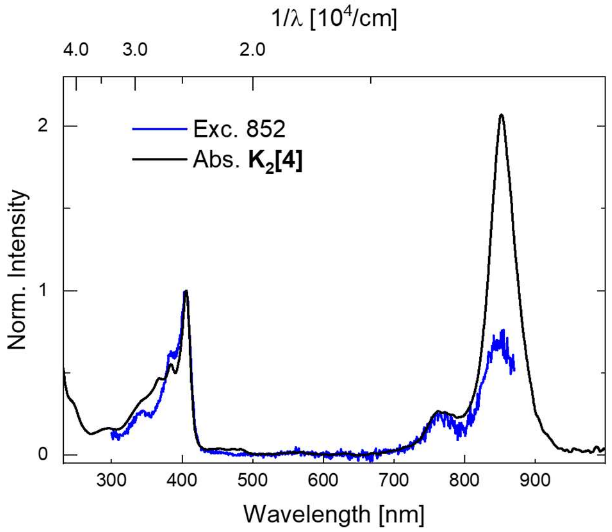

Figure S36: Normalized (to the band at 405 nm) absorption (black) and associated excitation-spectrum of **K_2[4]** (set $\lambda_{emission}$: 852 nm) in THF.

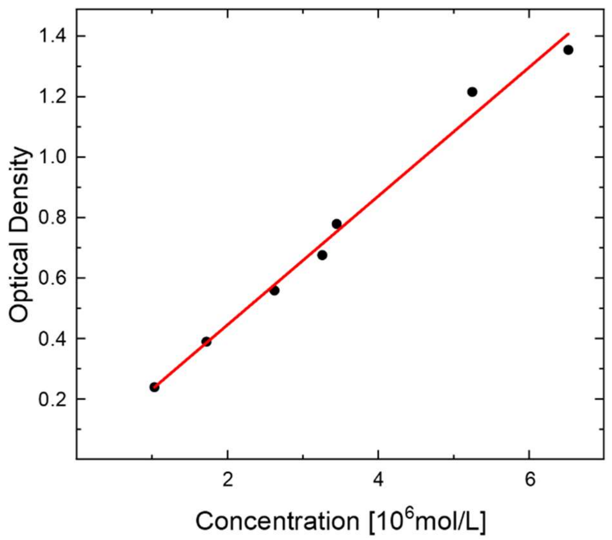

Figure S37: Linear fit of several measurements to determine extinction coefficient of **K_2[4]**. The optical density of the lowest energy band (852 nm) was considered for the calculation (R^2 = 0.98856).

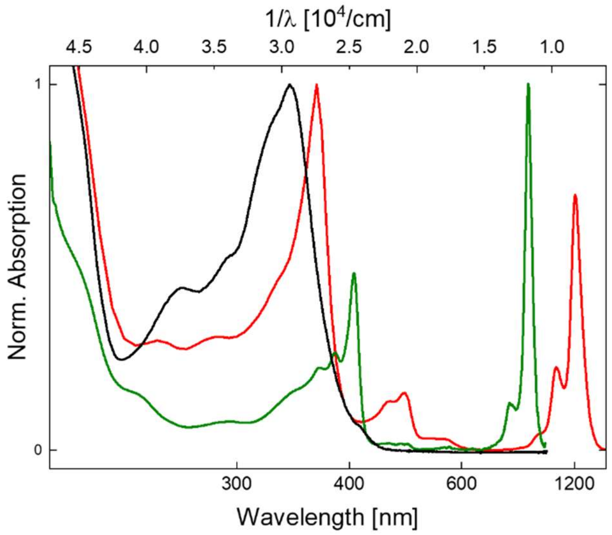

Figure S38: Normalized absorption spectra of macrocycles **4** (black), **K[4]** (red) and **K$_2$[4]** (green)**.** The absorptions band with the highest optical density was normalized to 1 for the respective compounds. It is plotted reciprocally over the wavelength.

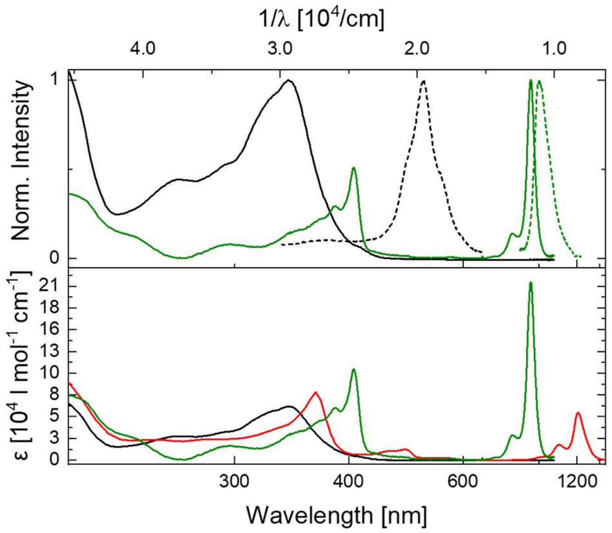

Figure S39: Top: Normalized absorption- (solid lines) and emission-spectra (dashed lines) of **4** (black) and **K$_2$[4]** (green). Bottom: Absorption spectra of **4** (black), **K[4]** (red) and **K$_2$[4]** (green) with respect to their extinction-coefficients.

Table S1: Photophysical data of compounds **1**[10], **2**[10], **3**[10], and **4**, **K[4]**, and **K_2[4]**.

No.	λ_{abs}[nm][a]	ε_{max} [10^3 $Lmol^{-1}$ cm^{-1}]	λ_{em}[nm][a]	Φ_f [%]	Stokes shift [meV]
**1**	265, **326**	24.2	**410**	3	779
**2**	276, 305, 356[b], **367**	23.9	-	-	-
**3**	279, 338, 371, **387**	35.8	-	-	-
**4**	267, **340**, 415[b]	64.4	**511**, 551[b]	1	1225[c], 659[d]
**K[4]**	**365**, 455, 480, 560, 1210	81.4	-	-	-
**K_2[4]**	406, **855**	213	**902**	1	76.1

[a] Highest absorption and emission maxima in bold

[b] shoulder

[c] Shift between maximum absorption band and maximum emission

[d] Stokes shift between lowest energy absorption band and maximum emission

Cyclic Voltammetry

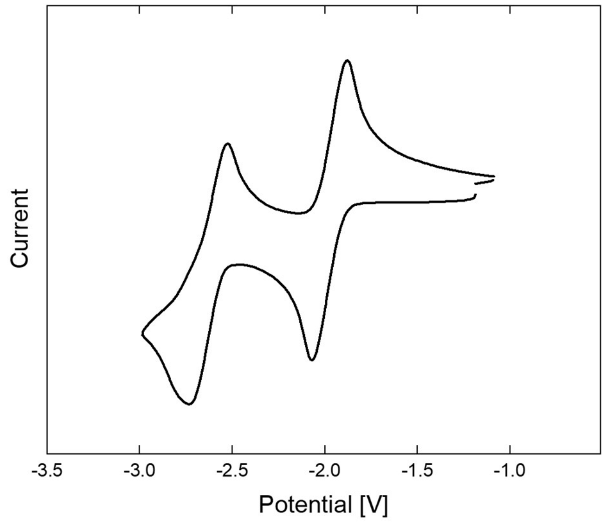

Figure S40: Cyclic voltammograms of **2** in THF (vs. $[Cp_2Fe]^{0/+}$, scan-rate: 250 mVs^{-1}); $E_{1/2,1}$: -1.97 V; $E_{1/2,2}$: -2.63 V.

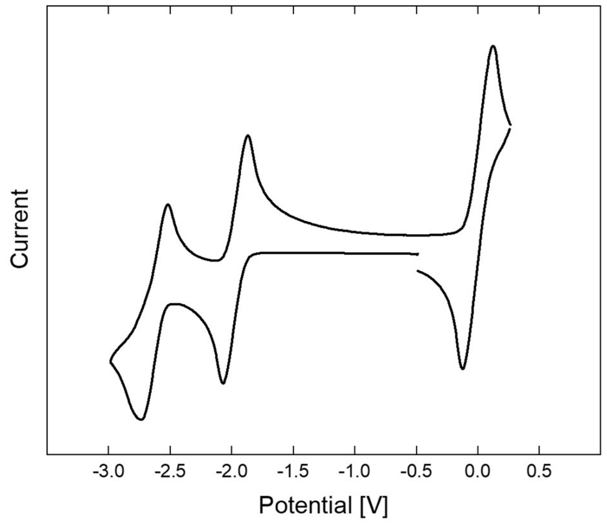

Figure S41: Cyclic voltammograms of **2** in THF with $[Cp_2Fe]^{0/+}$ in the mixture (scan-rate: 250 mVs^{-1}). This voltammogram was used to determine the values of the half-wave potentials.

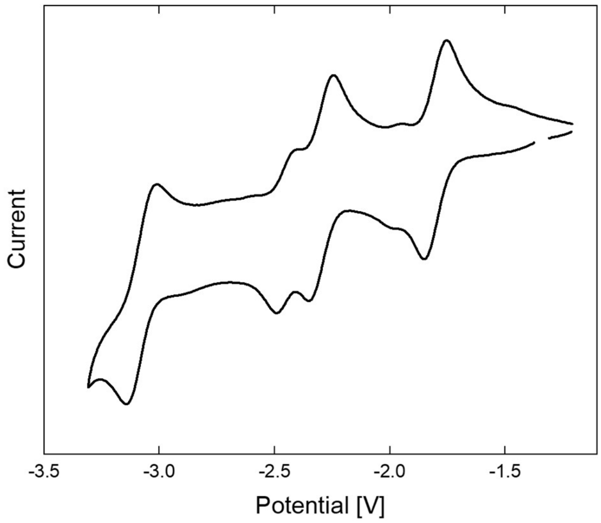

Figure S42: Cyclic voltammograms of **3** in THF (vs. $[Cp_2Fe]^{0/+}$, scan-rate: 250 mVs^{-1}); $E_{1/2,1}$: -1.80 V; $E_{1/2,2}$: - 2.30 V; $E_{1/2,3}$: - 3.08 V.

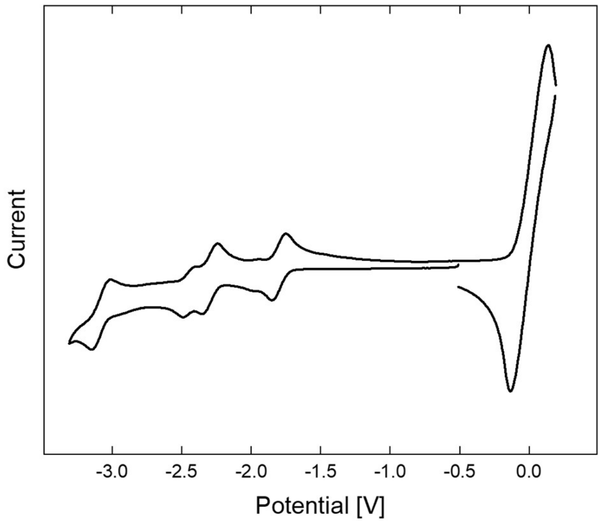

Figure S43: Cyclic voltammograms of **3** in THF with $[Cp_2Fe]^{0/+}$ in the mixture (scan-rate: 250 mVs^{-1}). This voltammogram was used to determine the values of the half-wave potentials.

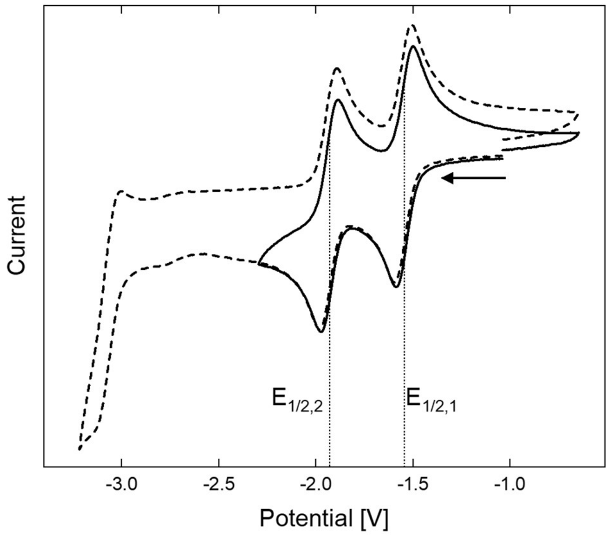

Figure S44: Cyclic voltammograms of **4** in THF (vs. $[Cp_2Fe]^{0/+}$, scan-rate: 250 mVs^{-1}); dashed line: whole reductive range; solid line: first two reversible events only. $E_{1/2,1}$: -1.52 V; $E_{1/2,2}$: -1.93 V.

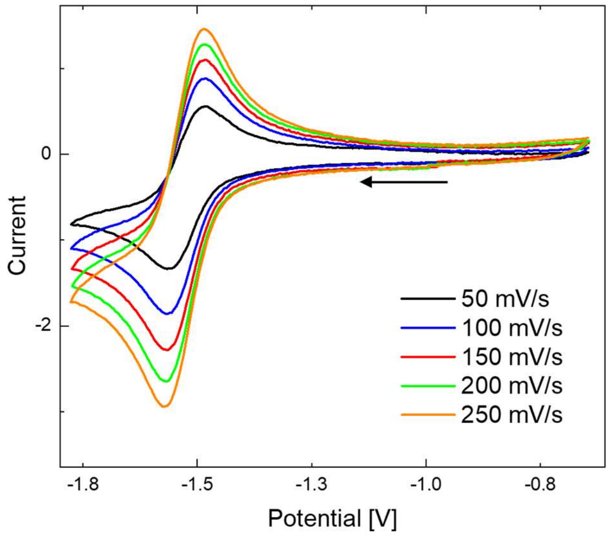

Figure S45: Cyclic voltammograms of the first reversible reduction of **4** in THF (vs. $[Cp_2Fe]^{0/+}$) at different scan-rates.

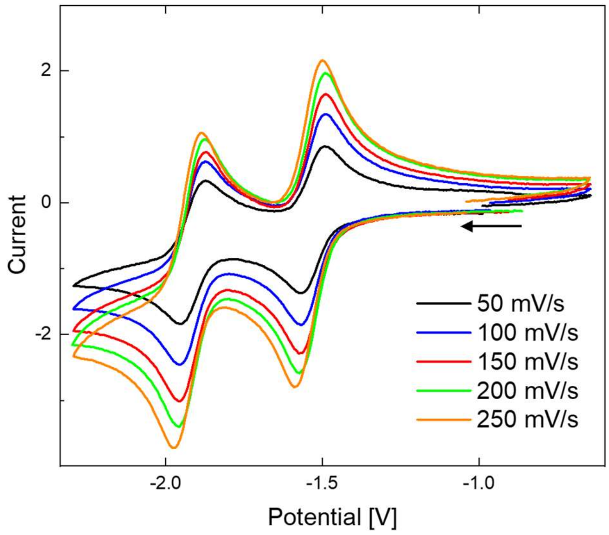

Figure S46: Cyclic voltammograms of the first two reversible reductions of **4** in THF (vs. $[Cp_2Fe]^{0/+}$) at different scan-rates.

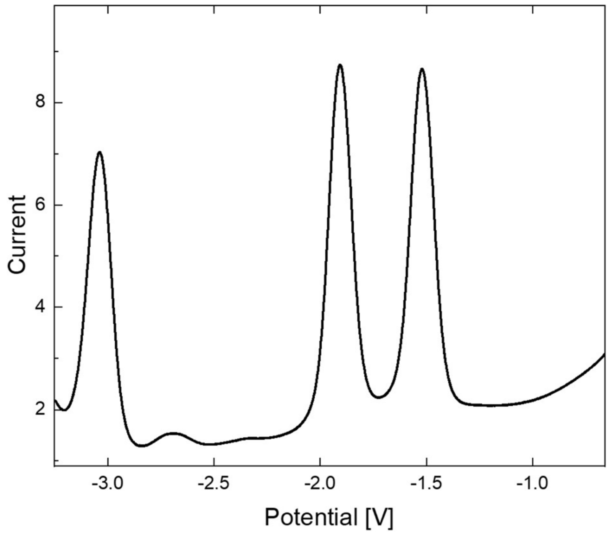

Figure S47: Square-Wave voltammogram of **4** in THF (vs. $[Cp_2Fe]^{0/+}$); 100 MHz, 0.5 mV step rate.

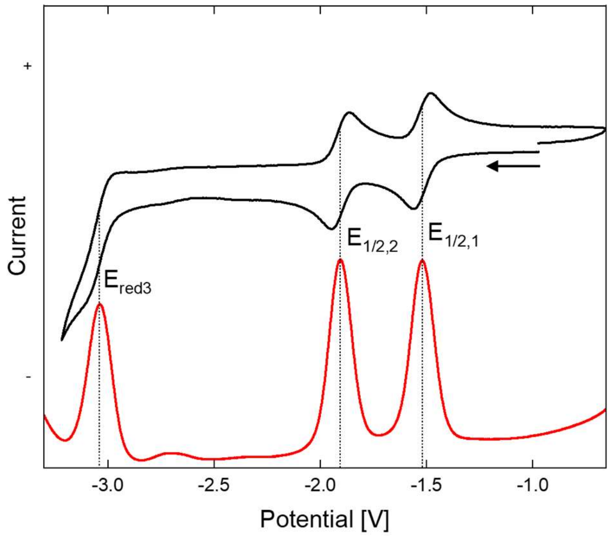

Figure S48: Cyclic voltammograms (black) and associated square wave voltammogram (red) of **4** in THF (vs. $[Cp_2Fe]^{0/+}$, scan rate: 150 mVs^{-1}). $E_{1/2,1}$: -1.52 V; $E_{1/2,2}$: -1.93 V; E_{red3}: -3.04 V.

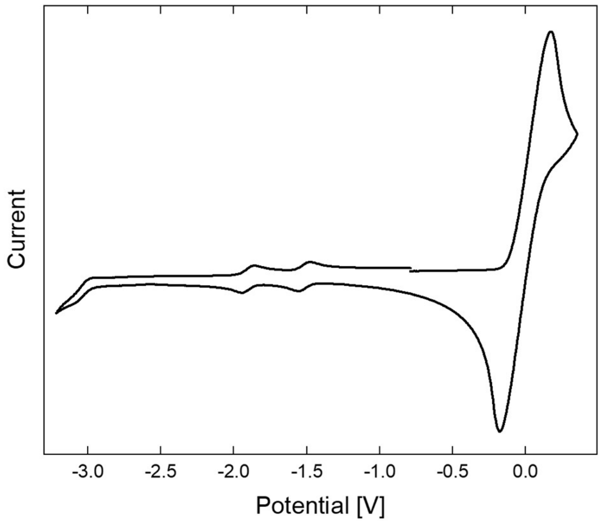

Figure S49: Cyclic voltammograms of **4** in THF with $[Cp_2Fe]^{0/+}$ in the mixture (scan-rate: 150 mVs^{-1}). This voltammogram was used to determine the values of the half-wave potentials.

Electron Paramagnetic Resonance Spectra

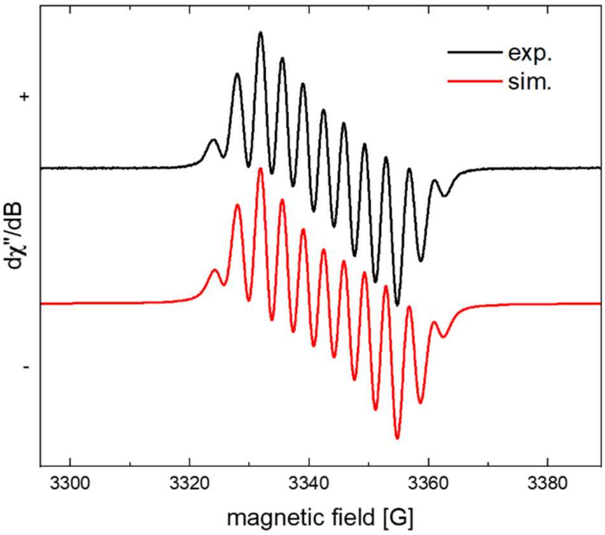

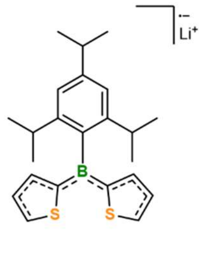

Li[1]

Figure S50: Experimental (black) and simulated (red) continuous-wave (CW) X-band EPR spectra of **Li[1]** in THF at room temperature. Experimental parameters: microwave frequency = 9.37 GHz; microwave power = 2 mW; modulation amplitude = 0.5 G; conversion time = 60 ms; modulation frequency = 100 kHz. The simulation parameters are g_{iso} = 2.0038, a($^{10/11}$B) = 19 MHz, a(^{1}H, 2H) = 10.3 MHz, a(^{1}H, 2H) = 11.6 MHz, and a(^{1}H, 2H) = 1.8 MHz.

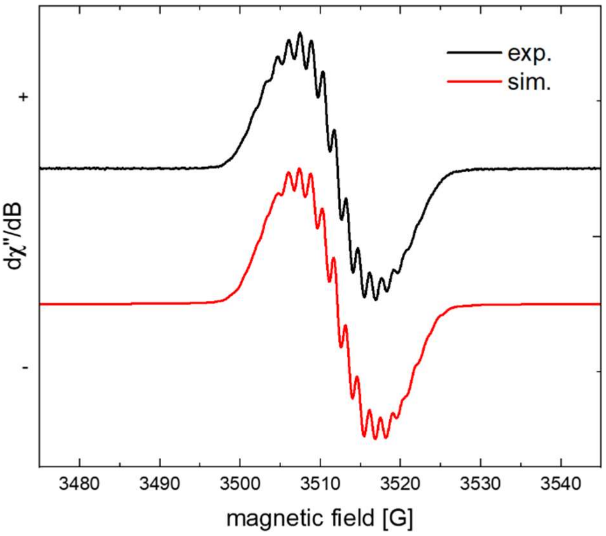

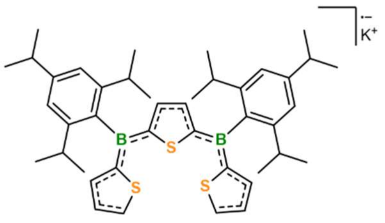

K[2]

Figure S51: Experimental (black) and simulated (red) continuous-wave (CW) X-band EPR spectra of **K[2]** in THF at room temperature. Experimental parameters: microwave frequency = 9.85 GHz; microwave power = 0.2 mW; modulation amplitude = 0.5 G; conversion time = 60 ms; modulation frequency = 100 kHz. The best-fit simulation parameters are g_{iso} = 2.0043, a($^{10/11}$B) = 7.6 MHz, a(^{1}H, 2H) = 5.4 MHz, a(^{1}H, 2H) = 5.1 MHz, a(^{1}H, 2H) = 2.2 MHz, and a(^{1}H, 2H) = 1.8 MHz (starting values derived from DFT calculations/EPR-II).

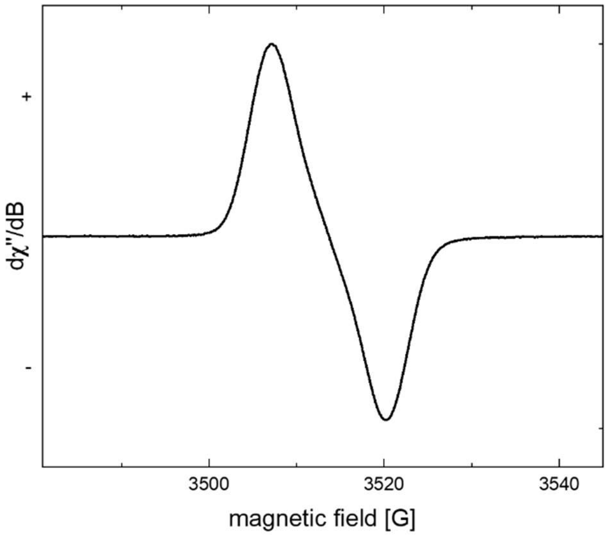

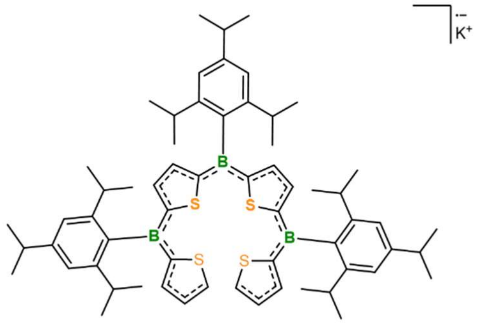

K[3]

Figure S52: Continuous-wave (CW) X-band EPR spectrum of **K[3]** in THF at room temperature. Experimental parameters: microwave frequency = 9.85 GHz; microwave power = 0.2 mW; modulation amplitude = 0.5 G; conversion time = 60 ms; modulation frequency = 100 kHz. g_{iso} = 2.0045.

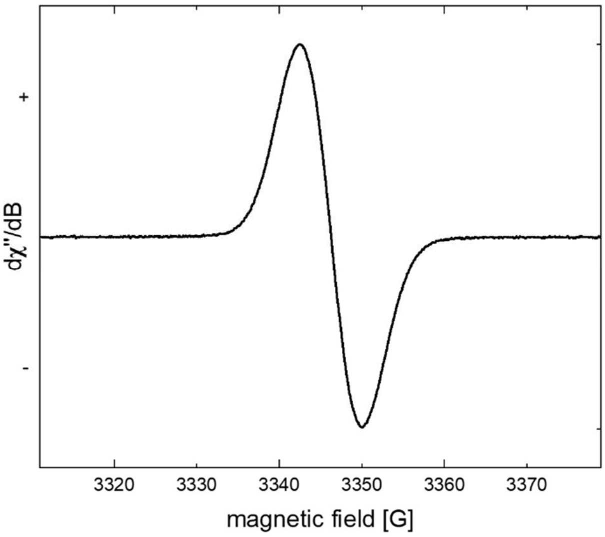

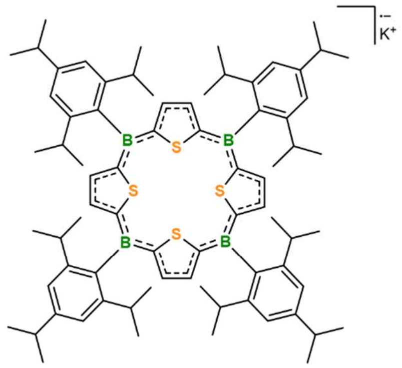

K[4]

Figure S53: Continuous-wave (CW) X-band EPR spectrum of **K[4]** in THF at room temperature. Experimental parameters: microwave frequency = 9.37 GHz; microwave power = 2 mW; modulation amplitude = 0.5 G; conversion time = 60 ms; modulation frequency = 100 kHz. g_{iso} = 2.0041.

Crystallographic Data

Table S2: Crystal structure and refinement data for **9**, **10** and **2**.

No.	9	10	2
**CCDC number**	2333889	2333893	2333890
**Size / mm**	0.298 x 0.342 x 0.372	0.351 x 0.438 x 0.468	0.261 x 0.320 x 0.430
**Empiric Formula**	'C34 H48 B2 Br2 S'	'C37 H56 B2 Cl2 O2 S'	'C42 H54 B2 S3'
**M**	670.22	657.39	676.36
**Crystal system**	monoclinic	orthorhombic	triclinic
**Space group**	'P 2/c'	'P c a 21'	'P -1'
**a/Å**	17.038(4)	25.290(8)	12.413(3)
**b/Å**	6.3964(13)	10.595(4)	12.952(3)
**c/Å**	16.709(6)	28.561(11)	13.234(3)
α/°	90	90	96.943(8)
β/°	107.42(2)	90	105.395(7)
γ/°	90	90	103.217(8)
**V/Å^3**	1737.4(8)	7653(5)	1959.3(7)
**Z**	2	8	2
μ**/mm^{-1}**	2.414	0.254	0.217
**T/K**	100(2)	100(2)	100(2)
$\theta_{min,max}$	1.253, 26.414	1.426, 26.476	2.529, 26.457
**Completeness**	0.999	0.996	0.975
**Reflections: total/independent**	3557, 3055	14428, 12940	7957, 6905
R_{int}	0.0494	0.0959	0.0663
**Final R1 and wR2**	0.0459, 0.1220	0.1078, 0.2263	0.0739, 0.1828
**Largest peak, hole/eÅ^{-3}**	1.095, -0.921	0.559, -0.992	0.812, -0.411
ρ_{calc}**/g cm^{-3}**	1.281	1.141	1.147

Table S3: Crystal structure and refinement data for **4**, **K[4]** and **K_2[4]**.

**No.**	**4**	**K[4]**	**K_2[4]**
**CCDC number**	2333888	2333892	2333891
**Size / mm**	0.134 x 0.173 x 0.429	0.222 x 0.279 x 0.366	0.254 x 0.308 x 0.387
**Empiric Formula**	'C88 H124 B4 O6 S4'	'C100 H148 B4 K O6 S4'	'C58 H90 B2 K O5 S2 '
**M**	1449.34	1656.76	992.13
**Crystal system**	monoclinic	triclinic	tetragonal
**Space group**	'C 2/c'	'P -1'	'P 4 n c'
**a/Å**	36.092(8)	14.848(3)	17.670(2)
**b/Å**	9.380(2)	15.062(2)	17.670(2)
**c/Å**	29.515(5)	23.574(4)	18.642(8)
α/°	90	78.942(9)	90
β/°	120.909(8)	83.888(7)	90
γ/°	90	72.934(7)	90
**V/Å^3**	8573(3)	4939.4(13)	5821(3)
**Z**	4	2	4
μ**/mm^{-1}**	0.160	0.188	0.207
**T/K**	100(2)	100(2)	100(2)
$\theta_{min,max}$	2.269, 26.393	2.076, 26.373	1.588, 26.500
**Completeness**	0.997	0.999	0.995
**Reflections: total/independent**	8772, 7457	20185, 17793	6011, 5350
R_{int}	0.0626	0.0532	0.0703
**Final R1 and wR2**	0.0475, 0.1129	0.0486, 0.1174	0.0490, 0.1318
**Largest peak, hole/eÅ^{-3}**	0.562, -0.526	0.808, -0.497	0.678, -0.393
ρ_{calc}**/g cm^{-3}**	1.123	1.114	1.132

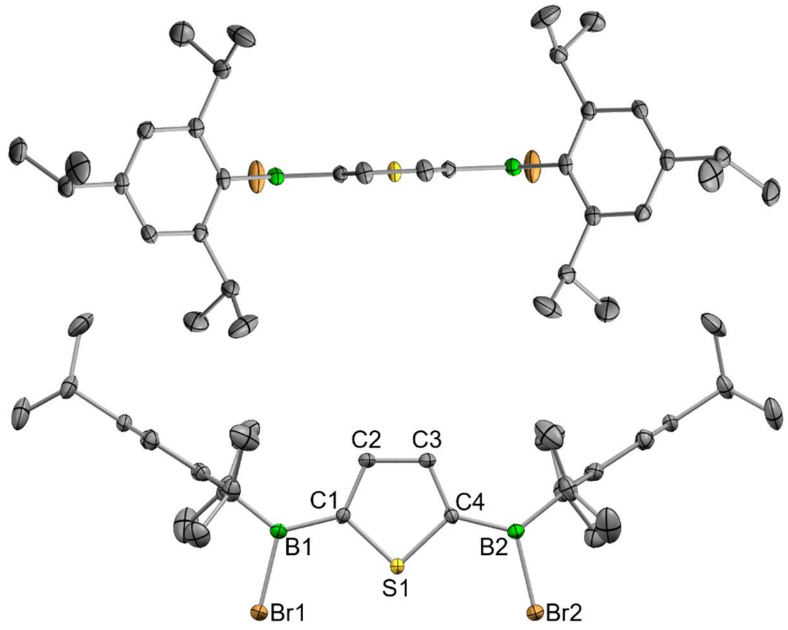

Figure S54: Molecular structure of **9** (top: view parallel to the thiophene plane; bottom: view perpendicular to the thiophene plane) in the solid state determined by single-crystal X-ray diffraction. All atomic displacement ellipsoids are drawn at the 50 % probability level. H-atoms are omitted for clarity.

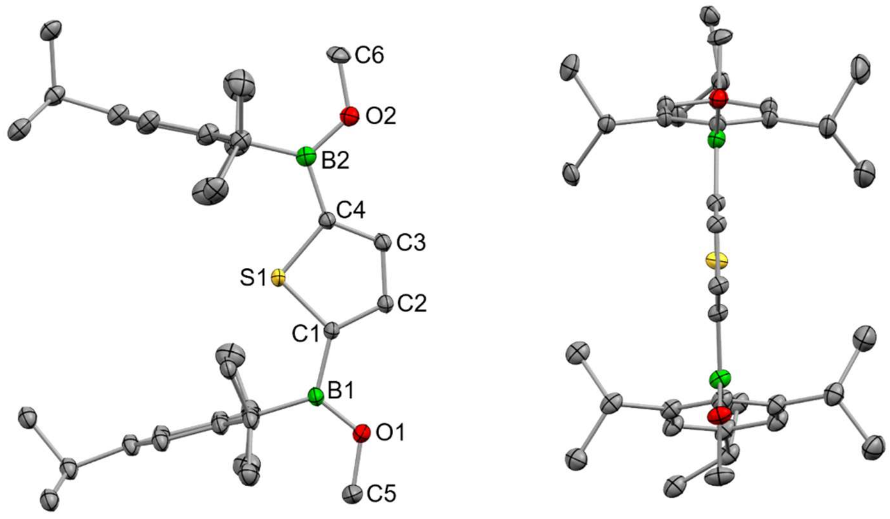

Figure S55: Molecular structure of **10** (left: view perpendicular to the thiophene plane; right: view parallel to the thiophene plane) in the solid state determined by single-crystal X-ray diffraction. All atomic displacement ellipsoids are drawn at the 50 % probability level. H-atoms are omitted for clarity. Solvent molecules (DCM), disorder of the *para*-isopropyl groups of Tip and second molecule in the cell are omitted for clarity.

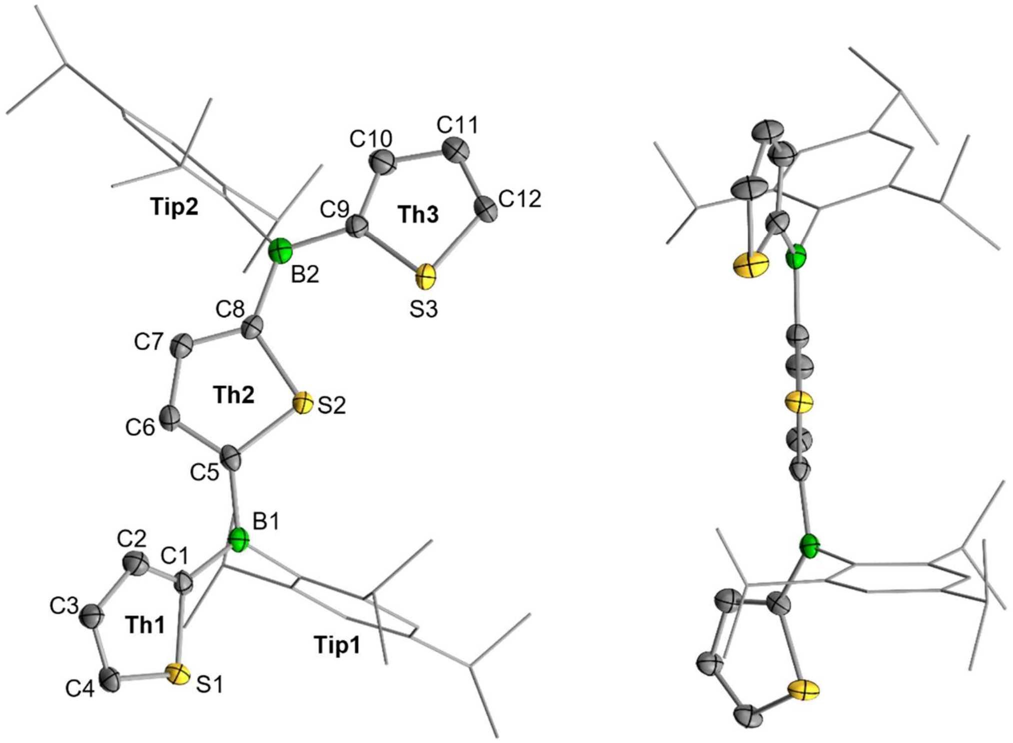

Figure S56: Molecular structure of **2** in the solid state determined by single-crystal X-ray diffraction. All atomic displacement ellipsoids are drawn at the 50 % probability level. H-atoms and are omitted for clarity. Terminal thiophenes are simplified and only one disorder is displayed. Tip1-2 and Th1-3 represent the respective 2,4,6-triisopropylphenyl (Tip) or thienyl (Th) units. Tip-substituents are depicted as wireframes for clarity.

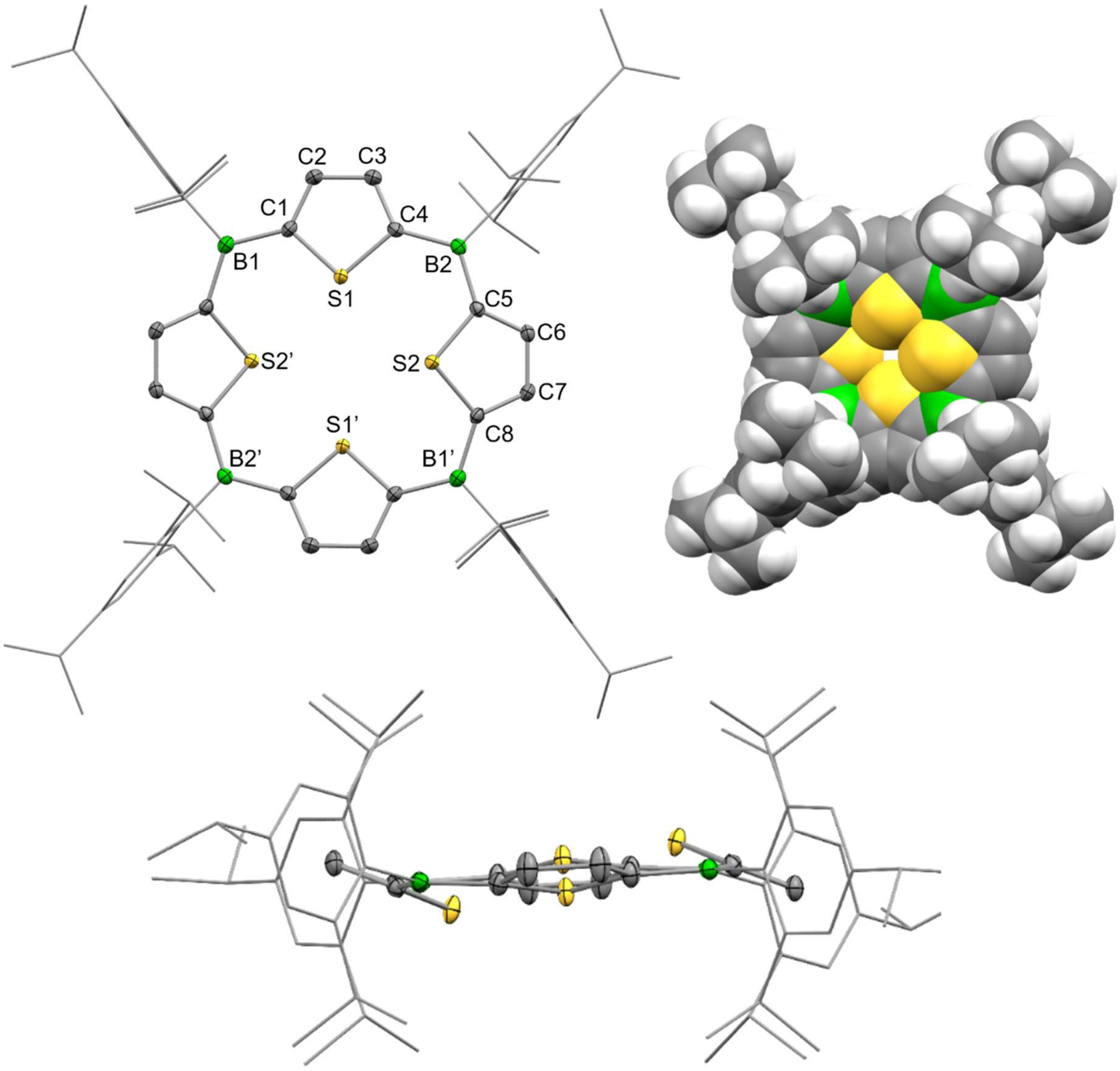

Figure S57: Molecular structure of **4** in the solid state determined by single-crystal X-ray diffraction. Heteroatoms marked with an apostrophe display the symmetry-generated ones. All atomic displacement ellipsoids are drawn at the 50 % probability level. H-atoms and solvent molecules (ethylacetate) are omitted for clarity. Tip-substituents depicted as wireframes for clarity. Top right: Spacefill-model revealing a narrow gap in the middle of the macrocycle. Bottom: Side view parallel to the mean-plane.

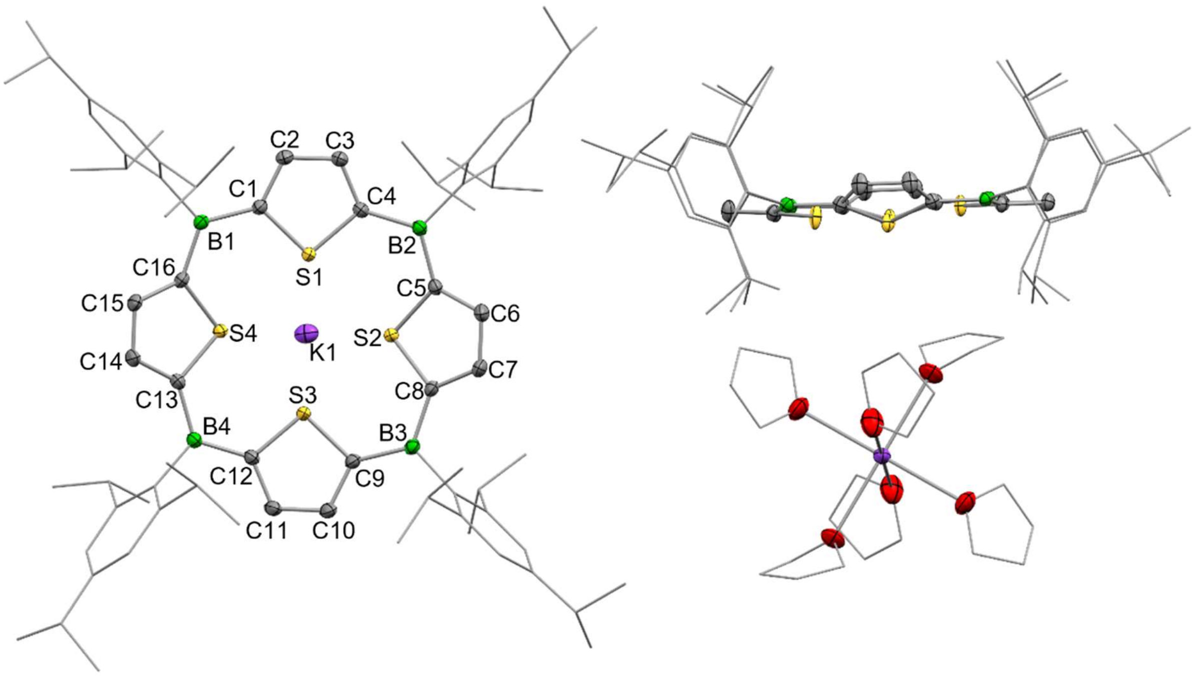

Figure S58: Molecular structure of **K[4]** (left: view perpendicular to the macrocyclic plane, in which THF molecules are omitted for clarity; right: view along the macrocyclic plane) in the solid state determined by single-crystal X-ray diffraction. All atomic displacement ellipsoids are drawn at the 50 % probability level. H-atoms are omitted for clarity. Tip-substituents and THF-carbon-scaffold depicted as wireframes for clarity.

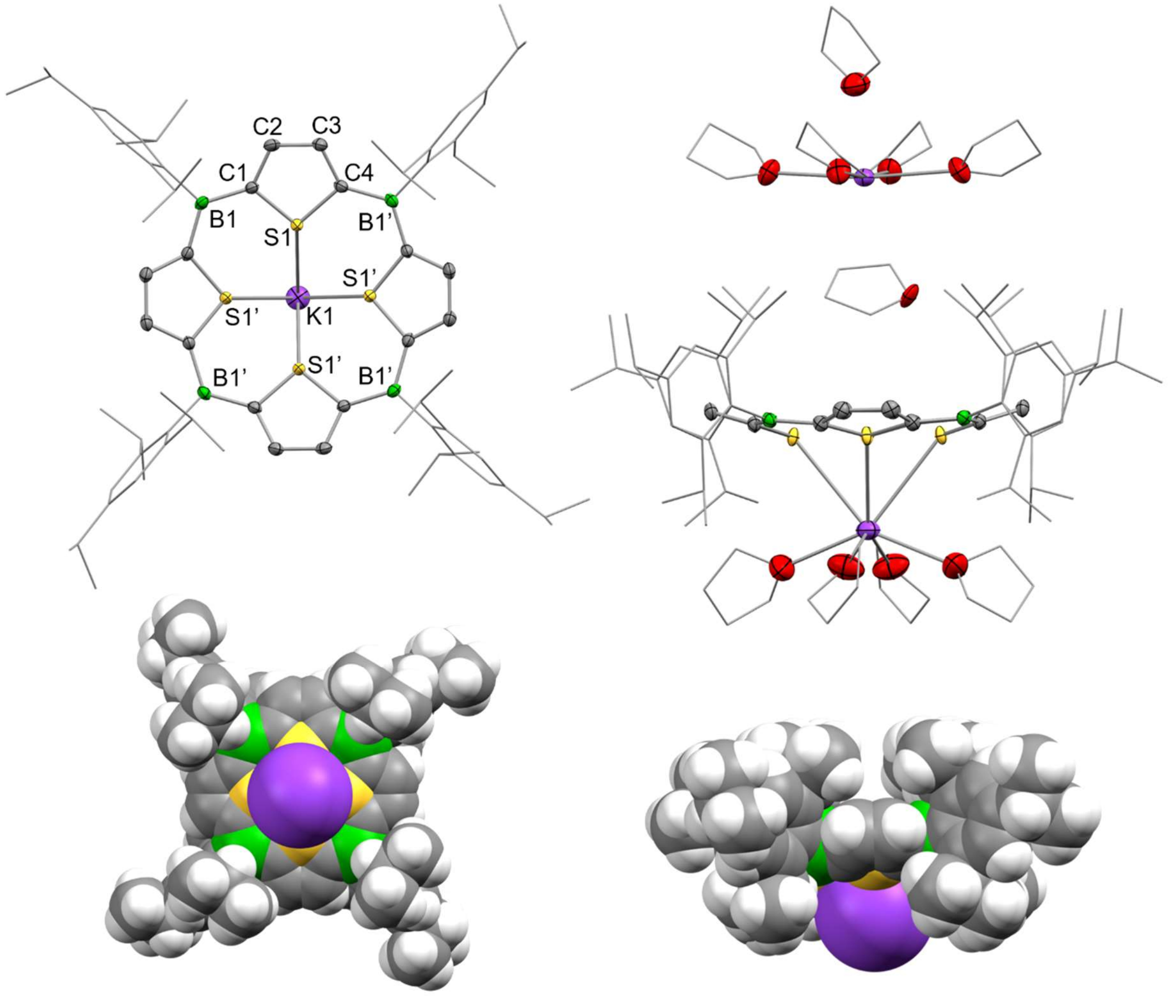

Figure S59: Molecular structure of **K_2[4]** (top left: view perpendicular to the macrocyclic plane, in which THF molecules and the non-coordinated potassium cation are omitted for clarity; top right: view along the macrocyclic plane) in the solid state determined by single-crystal X-ray diffraction. Heteroatoms marked with an apostrophe display the symmetry-generated ones. All atomic displacement ellipsoids are drawn at the 50 % probability level. H-atoms are omitted for clarity. Tip-substituents depicted as wireframes for simplicity-reasons. Bottom: Spacefill-models; left: perpendicular to the mean-plane; right: along the mean-plane.

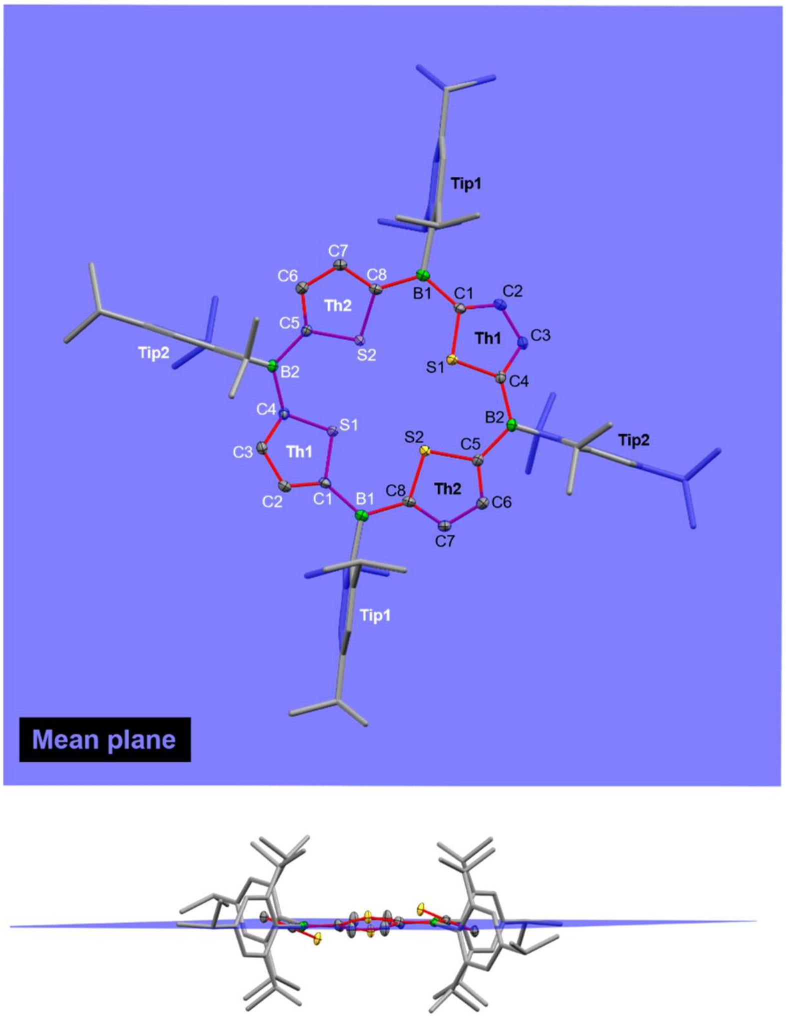

Figure S60: Mean-plane (MP) of **4** (blue plane). All atoms constructing the core of the macrocycle were used to determine the Mean-plane-deviation (MPD) (atoms connected *via* red bonds). Tip1-4 and Th1-4 represent the respective 2,4,6-triisopropylphenyl (Tip) or thienyl (Th) units. Atoms and units labelled in white represent parts of the compound generated by symmetry.

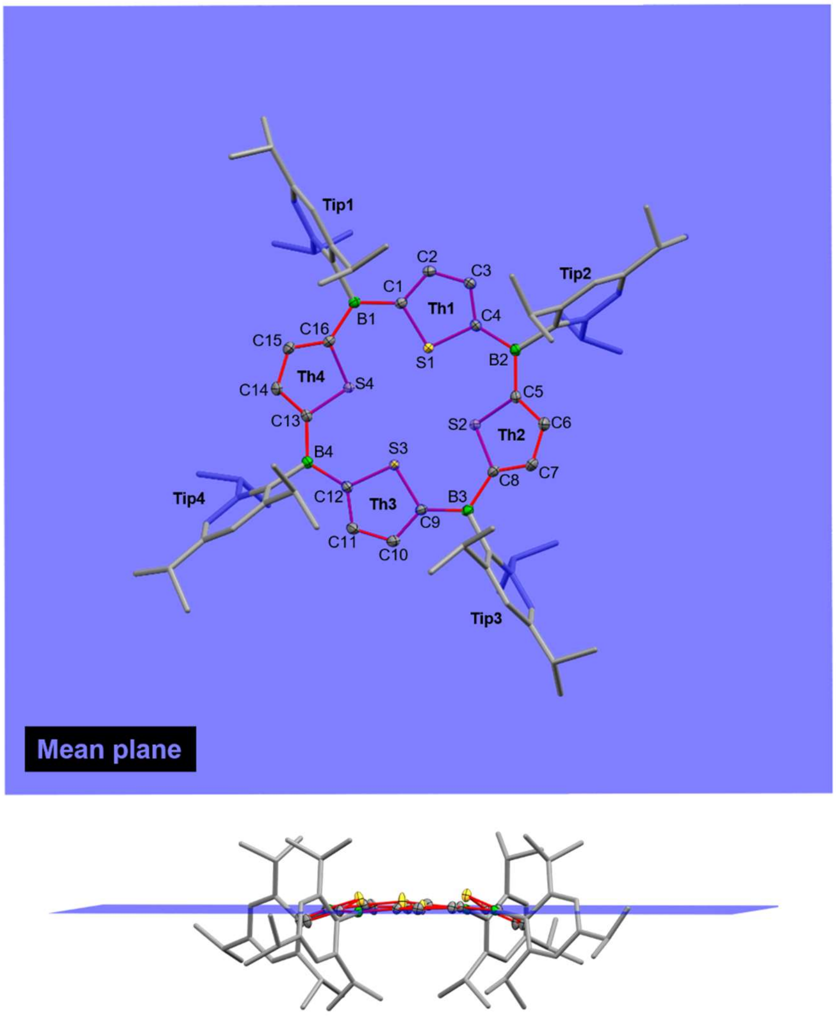

Figure S61: Mean-plane (MP) of **K[4]** (blue plane). All atoms constructing the core of the macrocycle were used to determine the Mean-plane-deviation (MPD) (atoms connected *via* red bonds). Tip1-4 and Th1-4 represent the respective 2,4,6-triisopropylphenyl (Tip) or thienyl (Th) units.

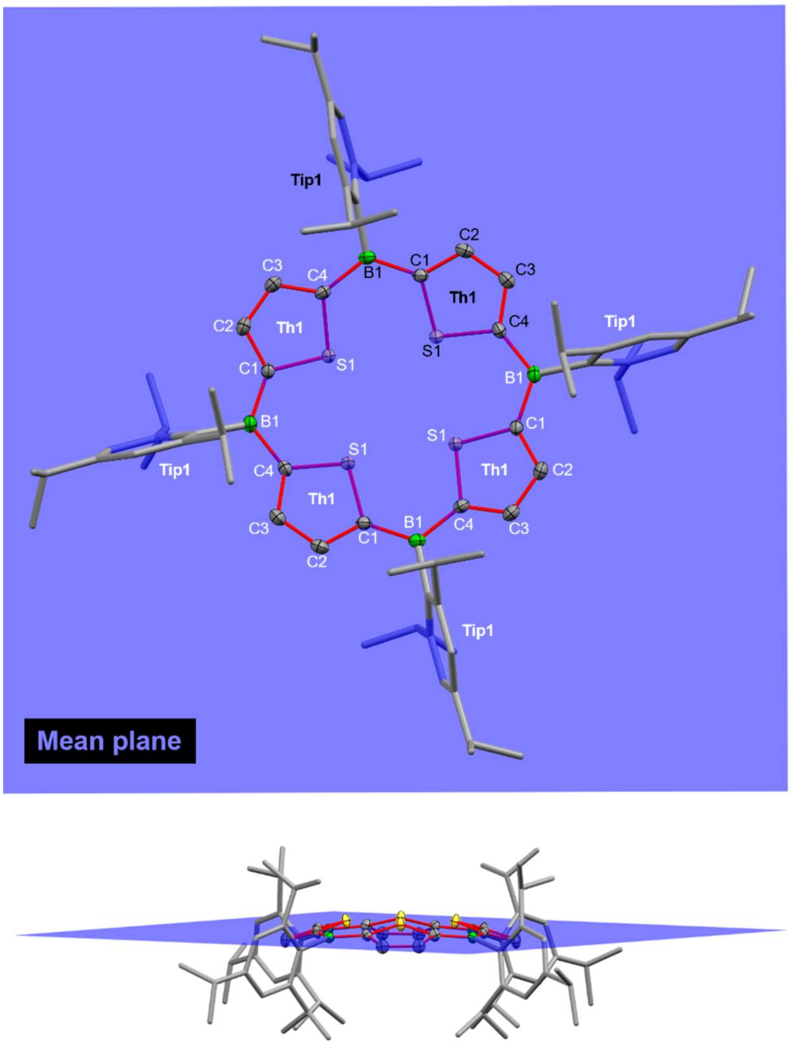

Figure S62: Mean-plane (MP) of **K_2[4]** (blue plane). All atoms constructing the core of the macrocycle were used to determine the Mean-plane-deviation (MPD) (atoms connected *via* red bonds). Tip1-4 and Th1-4 represent the respective 2,4,6-triisopropylphenyl (Tip) or thienyl (Th) units. Atoms and units labelled in white represent parts of the compound generated by symmetry.

Table S4: Structural data of the macrocycles **4**, **K[4]** and **K_2[4]** derived from the respective crystal structures. See Fig. S56, S60, S61 and S62 for details regarding the assignments of the respective abbreviations and atoms. Values for **1** were obtained from literature[9]. In case of **1** and **2** the mean values of the respective terminal disordered thiophenes are given. Bond lengths that are not debatable due to *disorder* are labelled as such.

		**1**	**2**	**4**	**K[4]**	**K_2[4]**
MPD [Å]		-	-	0.204	0.180	0.202
∡ BR_3 [°]	B1	360.00	359.90	359.98	359.82	359.57
	B2	-	359.90	359.63	359.76	
	B3	-	-		359.63	
	B4	-	-		359.61	
	Average	360.00	359.90	359.81	359.71	359.57
Dihedral angle between Tip plane and MP [°]	Tip1	-	-	88.22	79.60	81.04
	Tip2	-	-	88.57	70.56	
	Tip3	-	-		76.52	
	Tip4	-	-		74.52	
	Average	-	-	88.40	75.30	81.04
Dihedral angle between Tip plane and BR_3-plane [°]	Tip1−B1	86.04	82.53	85.37	81.59	82.18
	Tip2−B2	-	81.70	88.50	74.29	
	Tip3−B3	-	-		78.61	
	Tip4−B4	-	-		79.15	
	Average	86.04	82.16	86.94	78.41	82.18
Dihedral angle between thiophene-plane and MP [°]	Th1	-	-	22.30	1.45	17.35
	Th2	-	-	11.34	26.20	
	Th3	-	-		5.45	
	Th4	-	-		20.42	
	Average	-	-	16.82	13.38	17.35
Dihedral angle between thiophene-plane and BR_3-plane [°]	Th1−B1	17.14	5.19	24.12	8.89	11.58
	Th1−B2 (Th2−B2 for **2**)	-	17.59	17.78	11.27	12.85[b]
	Th2−B2	-	34.11	9.70	18.13	
	Th2−B3 (Th3−B3 for **2**)	-	14.04	13.41[a]	16.52	
	Th3−B3	-	-		11.34	
	Th3−B4	-	-		11.40	
	Th4−B4	-	-		11.83	
	Th4−B1 (Th2−B1 for **1**)	17.35	-		12.88	
	Average	17.25	14.04	16.25	12.78	12.22
Dihedral angle between thiophene-planes [°]	Th1-Th2	30.40	18.88	27.48	27.65	24.35[c]
	Th2-Th3	-	44.64		26.79	
	Th3-Th4	-	-		21.16	
	Th4-Th1	-	-		18.97	
	Average	30.40	31.76	27.48	23.64	24.35
Bond-Lengths [Å] (deviations in parentheses)	B1−C1	1.532(3)	1.541(7)	1.562(2)	1.546(3)	1.526(4)
	C1−C2	*disorder*	*disorder*	1.385(3)	1.403(3)	1.412(4)
	C2−C3	*disorder*	*disorder*	1.408(2)	1.397(2)	1.393(4)
	C3−C4	*disorder*	*disorder*	1.392(3)	1.402(2)	1.410(4)
	C1−S1	*disorder*	*disorder*	1.720(2)	1.732(2)	1.736(3)
	C4−S1	*disorder*	*disorder*	1.720(2)	1.729(2)	1.741(3)
	C4−B2	-	-	1.555(2)	1.547(2)	1.533(4)[b]
	B2−C5 (B1−C5 for **1/2**)	1.542(3)	1.554(5)	1.556(3)	1.547(3)	
	C5−C6	*disorder*	1.381(5)	1.389(2)	1.398(2)	
	C6−C7	*disorder*	1.400(4)	1.404(3)	1.400(3)	
	C7−C8	*disorder*	1.381(6)	1.386(2)	1.395(3)	
	C5−S2	*disorder*	1.733(4)	1.724(2)	1.727(2)	
	C8−S2	*disorder*	1.731(4)	1.717(2)	1.732(2)	
	C8−B3 (C8−B2 for **2**)	-	1.550(5)	1.558(3)[a]	1.547(3)	
	B3−C9 (B2−C9 for **2**)	-	1.539(7)		1.543(3)	
	C9−C10	-	*disorder*		1.392(3)	
	C10−C11	-	*disorder*		1.398(2)	
	C11−C12	-	*disorder*		1.393(3)	
	C8−S3	-	*disorder*		1.729(2)	
	C12−S3	-	*disorder*		1.725(2)	
	C12−B4	-	-		1.552(2)	
	B4−C13	-	-		1.541(3)	
	C13−C14	-	-		1.399(2)	
	C14−C15	-	-		1.398(3)	
	C15−C16	-	-		1.399(3)	
	C13−S4	-	-		1.725(2)	
	C16−S4	-	-		1.727(2)	
	C16−B1	-	-		1.540(3)	
	S1-K	-	-	-	-	3.390

[a] Due to symmetry generated parts, B3 equals B1 in this case
[b] Due to symmetry generated parts, B2 equals B1 in this case
[c] Due to symmetry generated parts, Th2 equals Th1 in this case

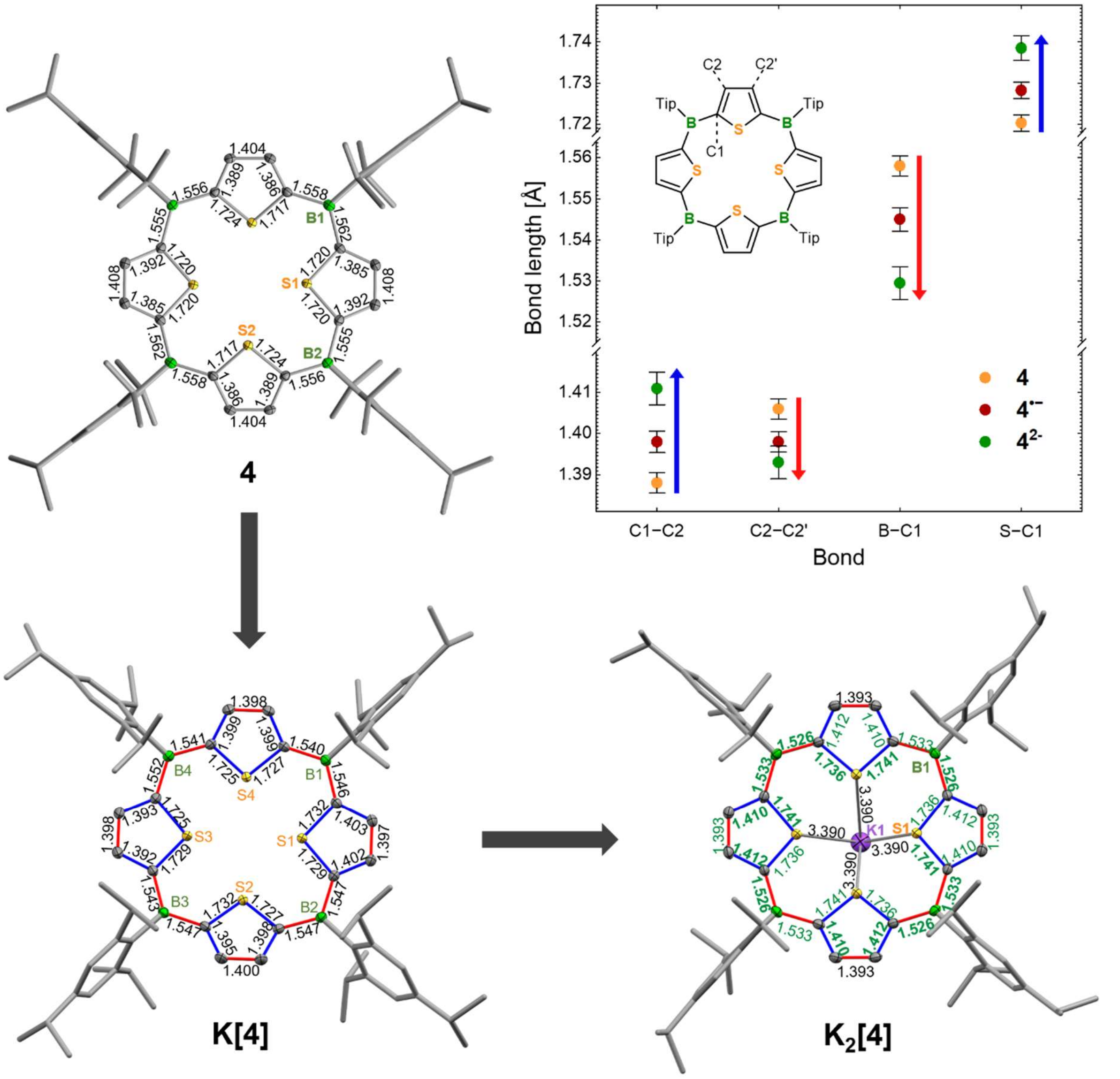

Figure S63: Comparison of the molecular structures of macrocycles **4**, **K[4]** and **K$_2$[4]** in the solid state and their bond length trends. Red and blue colored bonds indicate that they have shortened or are elongated, respectively, upon one-electron reduction. The bond lengths (in Å) at the respective bonds are given in bold if root-mean-square deviations compared to the species with one higher oxidation state are statistically significant. The green highlighted bond lengths for **K$_2$[4]** depict statistically significant changes compared to **4**. Ellipsoids of Tip substituents, THF molecules coordinating the potassium-cation in **K[4]**, non-coordinated potassium cations in the periphery of **K[4]** and **K$_2$[4]** and hydrogen-atoms are omitted for clarity. Right: Graphical illustration of the bond-length trends. To compare the specific bonds the average bond lengths were used for the calculations as **4** and **K$_2$[4]** inherit symmetry-generated parts in comparison to **K[4]**. Arrows indicate trends from **4** (orange) and **4$^{•-}$** (red) to **4^{2-}** (green). The markers at the nodes represent the average margin of error derived from the crystal structure for the respective bond lengths.

a

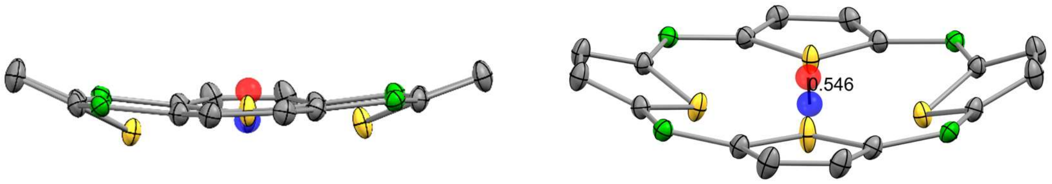

b

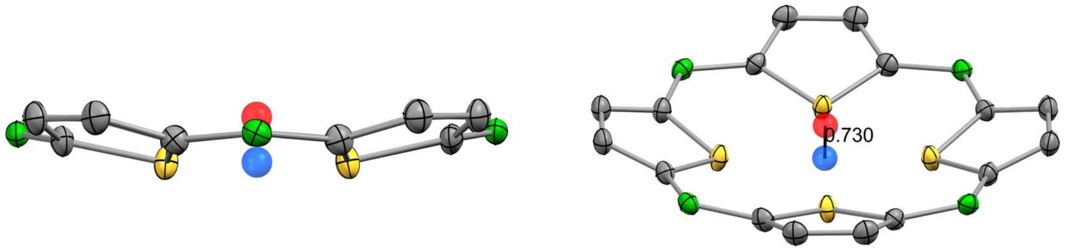

c

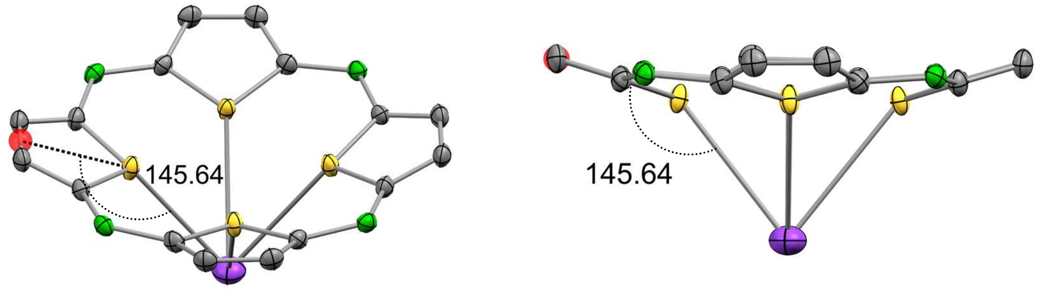

Figure S64: **a,b**, Bowl-depth-variation from **4**$^{\bullet-}$ (**a**) to **4**$^{2-}$ (**b**). Upon transition to the dianion a small increase in the bowl-depth is observed. Everything but the main-macrocyclic structure is omitted for clarity. Values are given in Ångström [Å]. Two centroids were used for each compound to determine the depth of the bowl-type structure. The upper centroid (red) is located in the middle of all backbone carbon atoms of the thiophene moieties. The lower centroid (blue) is situated in the middle of the sulfur-atoms. **c**, Bond-angle [°] of the sulfur-coordinated potassium-cation (purple) with respect to the thiophene plane. A centroid (red) between the backbone-carbon atoms, the sulfur atom (yellow) and the potassium cation served as the reference-points to determine the angle.

Computational methods

DFT geometry optimizations were carried out with the Gaussian 16, Revision C.01 program package[11] using the ωB97X-D[12] functional in combination with 6-31+G(d,p)[13-20] basis set in gas phase. The equilibrium geometries were characterized as minima by frequency computations. Vertical singlet excitations were calculated by means of time-dependent DFT with the program ORCA 5[21,22] using the ωB97X-D3[23] functional with optimal tuned ω parameters[24-26], the def2-SVP[27] basis set and the CPCM[28] solvation model mimicking tetrahydrofuran (ε = 7.25) as solvent.

The standard approach involves fitting the ω value along with other parameters concerning various ground state properties.[23] Within the optimal tuning procedure, the ω parameter is used to enforce Koopman's theorem as far as possible. In this procedure, ω is varied to minimize the sum of the energy difference between the computed ionization potential and the HOMO orbital energy and the energy difference between the computed ionization potential of the anionic system and the LUMO orbital energy of the anionic system.[24-26] Kronik and Baer[24-26] suggested this procedure since these energy differences should be zero for the exact Kohn-Sham functional.[29] It was shown that the description of charge transfer states (CT) is markedly improved if the optimal tuned functional is employed[24] especially if you compare its functionals with missing long-range-correction like e.g. B3LYP[30] which are not the best choice for the computation of those kind of states.[31]

Our results also suggest that the tuning approach in combination with the def2-SVP basis set and the CPCM[28] solvation model mimicking tetrahydrofuran is an appropriate manner to reproduce the experimental spectrum of the macrocyclic system so that it was also applied for the other systems. Because of the fact that the bonds between the boron centre and the thiophene units are freely rotatable in **1**, **2**, and **3** there is high number of isomers that are not all discussed here. We picked the isomers that fit best the experimental spectra and are energetically favourable.

The optimization and spin density computation of the radical compounds $\mathbf{1}^{\bullet-}$, $\mathbf{2}^{\bullet-}$, $\mathbf{3}^{\bullet-}$ and $\mathbf{4}^{\bullet-}$ were performed at the UωB97X-D[11]/6-31+G(d,p)[13-20] level of theory using the Gaussian 16, Revision C.01 program package.[11] The hyperfine coupling constants (HFCCs) of the compounds $\mathbf{1}^{\bullet-}$ and $\mathbf{2}^{\bullet-}$ were carried out with the functional UωB97X-D in combination with the basis sets 6-311+G(d,p)[13,16,20,32] and EPR-II.[33] The former one

was merely used on the sulfur atoms, whereas the latter one was used for the remaining atoms.

Single-point energy calculations for the fluoride adducts were performed with ωB97X-D/6-311+G(d,p) basis set and the PCM[34] solvation model mimicking THF (ε = 7.4257) as solvent using the Gaussian 16, Revision C.01 program package. Thermodynamic parameters were calculated at a temperature of 298.15 K and a pressure of 1.00 atm. A concentration correction of $\Delta G^{0\rightarrow *} = RT \cdot \ln(24.46) = 1.89$ kcal mol^{-1} (T = 298.15 K) was added to the free energies of all calculated species. This was done to change the 1.00 atm gas phase values to the condensed phase standard state concentration of 1.00 mol·L^{-1}, which leads to a proper description of associative/dissociative steps. This is necessary because pure gas estimations overestimate the entropy penalty for the formation of complexes.[35]

NICS-scans[36,37] (nucleus-independent chemical shift) and ACID[38] (anisotropy of the current-induced density) calculations were also carried out using the Gaussian 16, Revision C.01 program package.

Computational Results

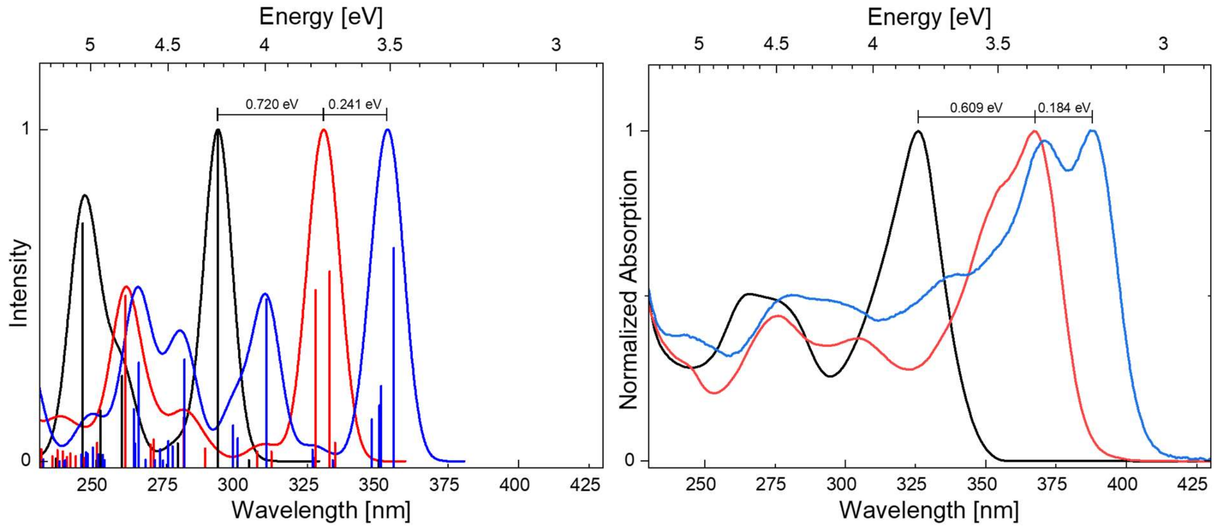

Figure S65: Left: Calculated absorption-spectra of compounds **1** (black), **2** (red), and **3**. Right: Experimental absorption spectra of **1** (black), **2** (red), and **3** in THF. The respective energy-differences between the maxima are shown.

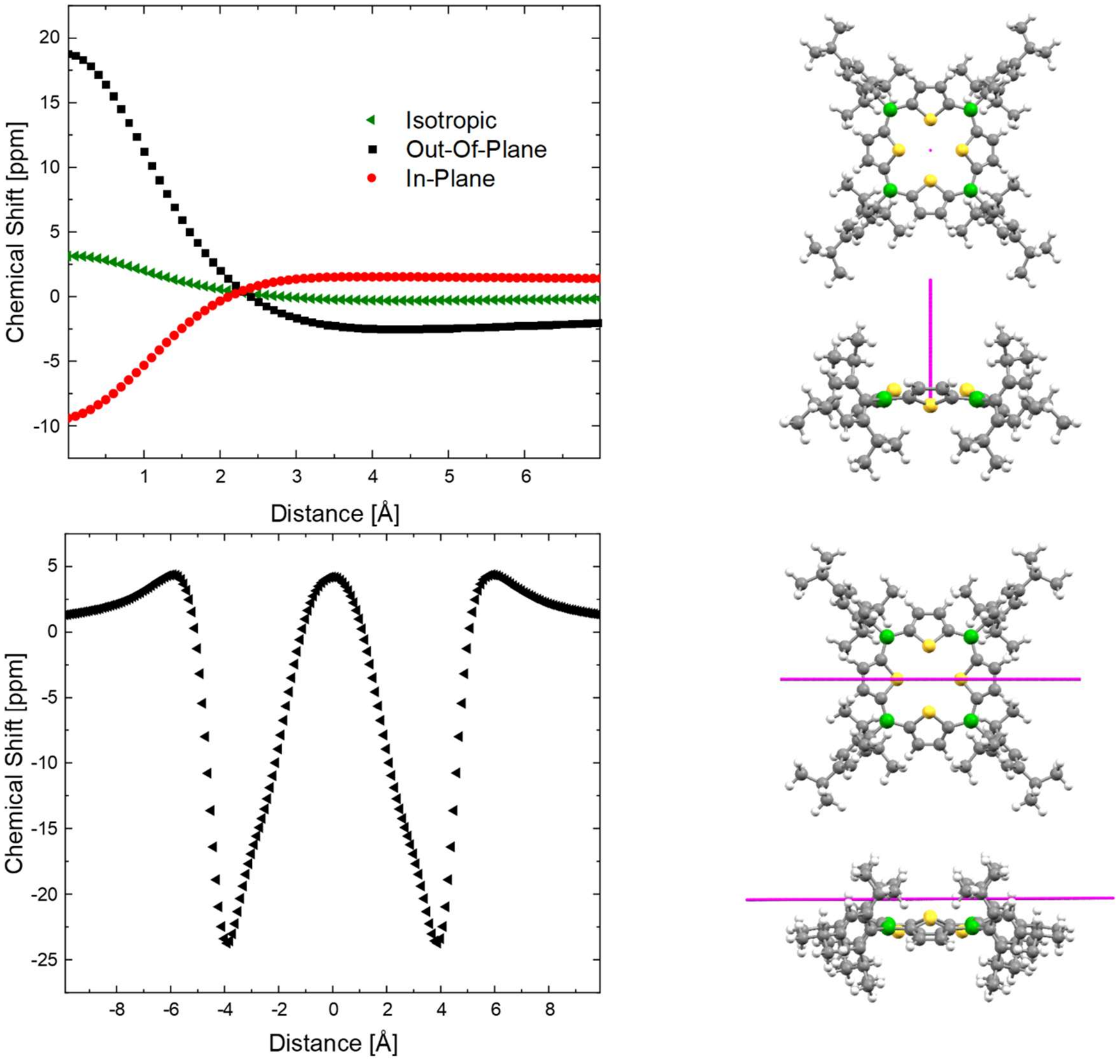

Figure S66: **Top**: NICS-Scan through **4**. The values were obtained *via* a dummy atom (purple) going from the middle of the molecule (Z = 0 Å) perpendicular to the mean-plane. **Bottom**: X-NICS-Scan of **4**. The observed dummy atom (purple) was moved parallel to the mean plane with a distance of Z = 1.7 Å to it.

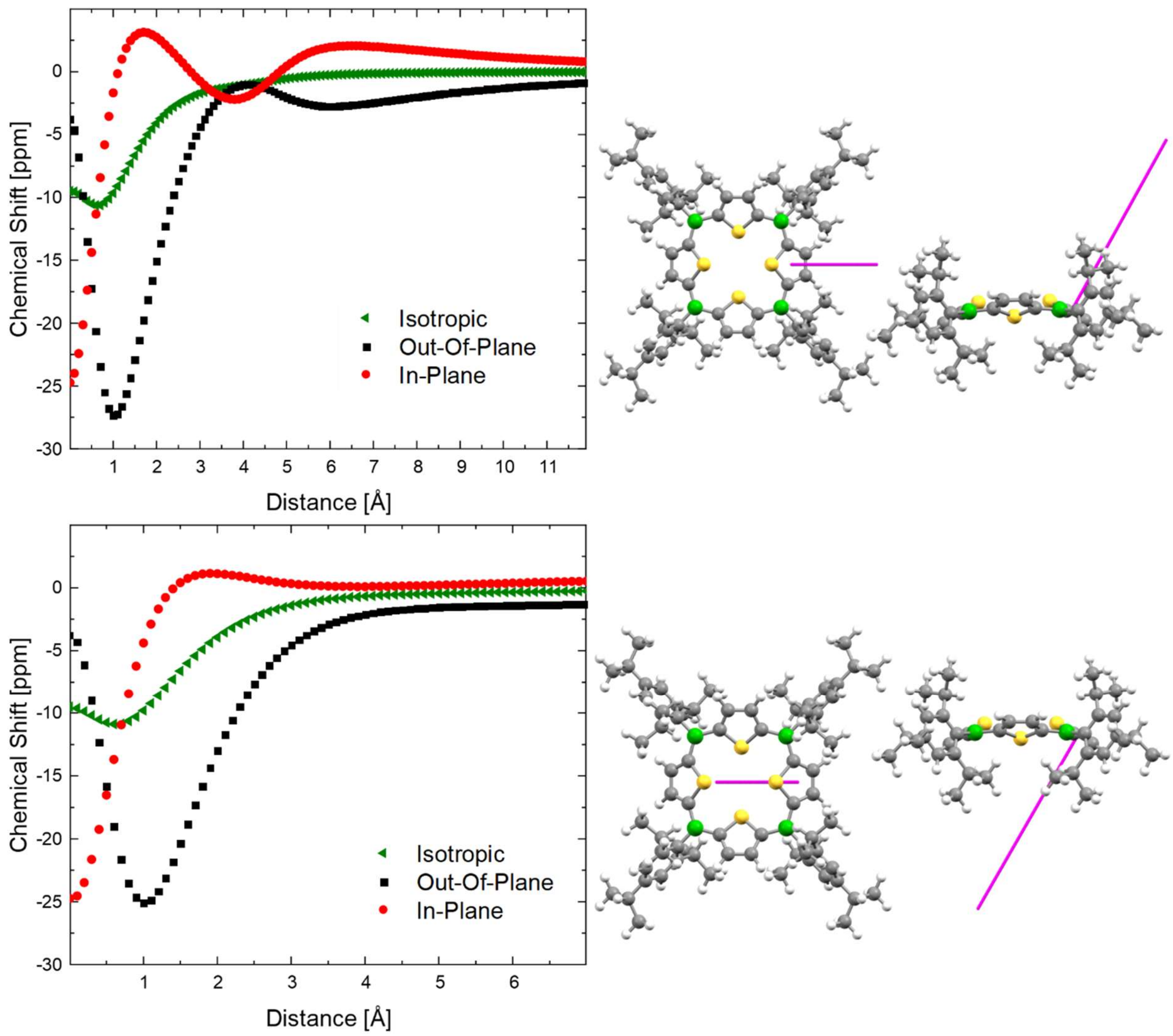

Figure S67: **Top**: NICS-Scan of the thiophene moiety of **4** going outwards. The values were obtained *via* a dummy atom (purple) going from the middle of the thiophene (Z = 0 Å) perpendicular to its-plane. **Bottom**: NICS-Scan of the thiophene moiety of **4** going inwards. The values were obtained *via* a dummy atom (purple) going from the middle of the thiophene (Z = 0 Å) perpendicular to its-plane

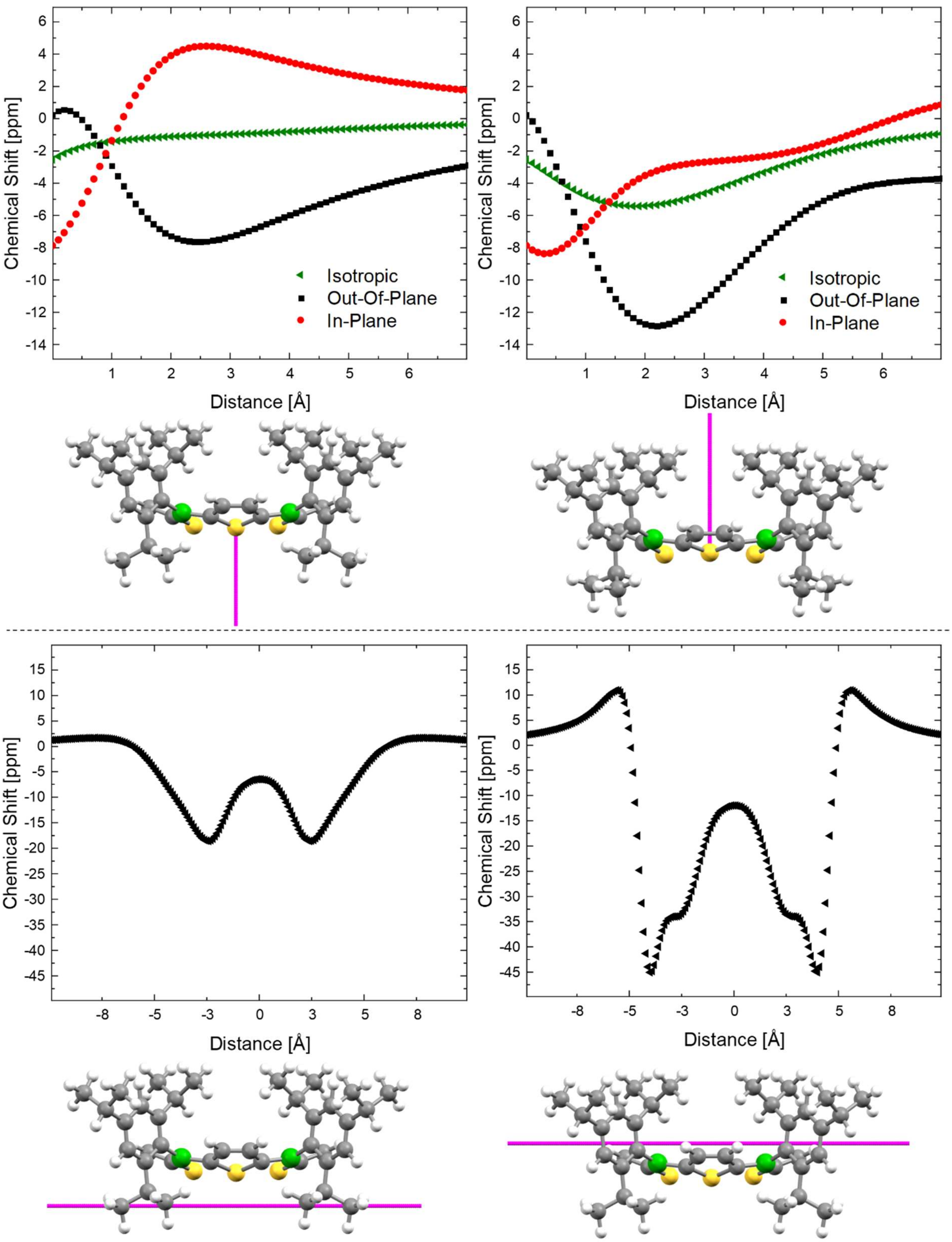

Figure S68: NICS-Scan of **$4^{\bullet-}$**: **Upper half**: Z-NICS-Scans; left side: dummy atom (purple) was moved perpendicular to the mean plane outwards the bowl-type structure; right side: dummy atom (purple) was moved perpendicular to the mean plane inwards the bowl-type structure. **Lower half**: X-NICS-Scans; left side: dummy atom (purple) was moved parallel to the mean plane on the convex side of the bowl-type structure; right side: dummy atom (purple) was moved parallel to the mean plane on the concave side the bowl-type structure. Both at a distance of Z = 1.7 Å.

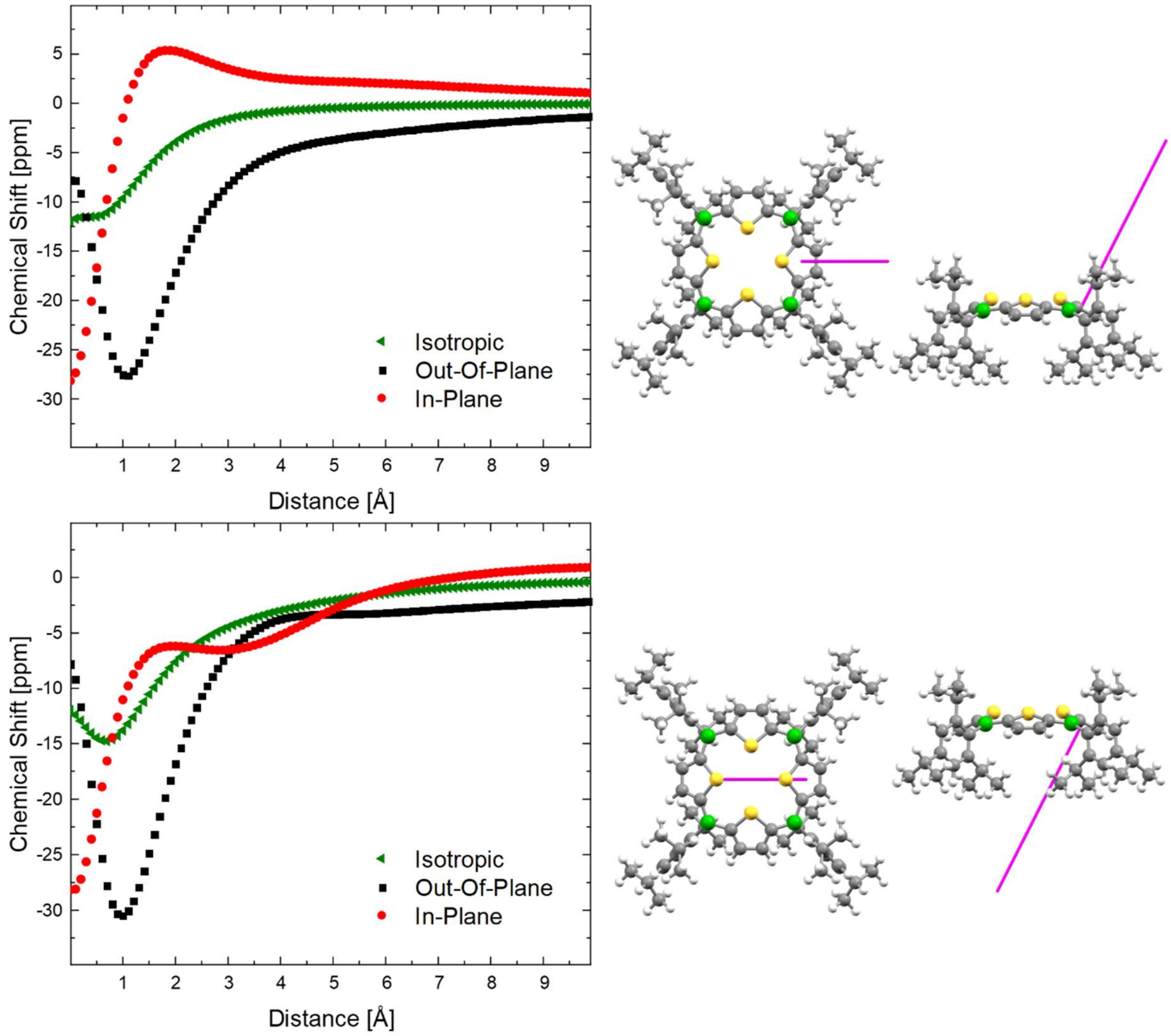

Figure S69: **Top**: NICS-Scan of the thiophene moiety of **4**$^{\bullet-}$ going outwards. The values were obtained *via* a dummy atom (purple) going from the middle of the thiophene (Z = 0 Å) perpendicular to its-plane. **Bottom**: NICS-Scan of the thiophene moiety of **4**$^{\bullet-}$ going inwards. The values were obtained *via* a dummy atom (purple) going from the middle of the thiophene (Z = 0 Å) perpendicular to its-plane

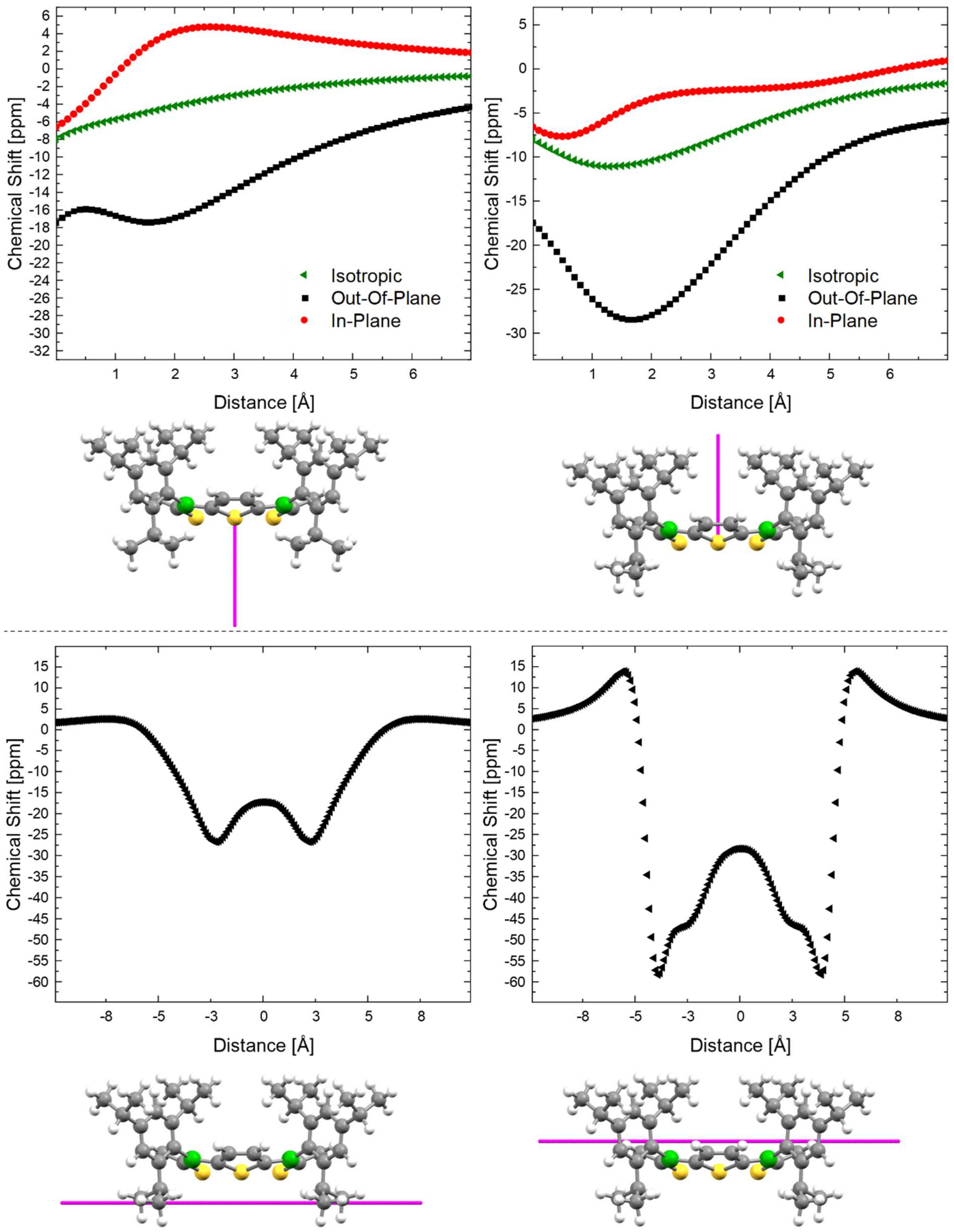

Figure S70: NICS-Scan of $\mathbf{4}^{2-}$: **Upper half**: Z-NICS-Scans; left side: dummy atom (purple) was moved perpendicular to the mean plane outwards the bowl-type structure; right side: dummy atom (purple) was moved perpendicular to the mean plane inwards the bowl-type structure. **Lower half**: X-NICS-Scans; left side: dummy atom (purple) was moved parallel to the mean plane on the convex side the bowl-type structure; right side: dummy atom (purple) was moved parallel to the mean plane on the concave side the bowl-type structure. Both at a distance of Z = 1.7 Å.

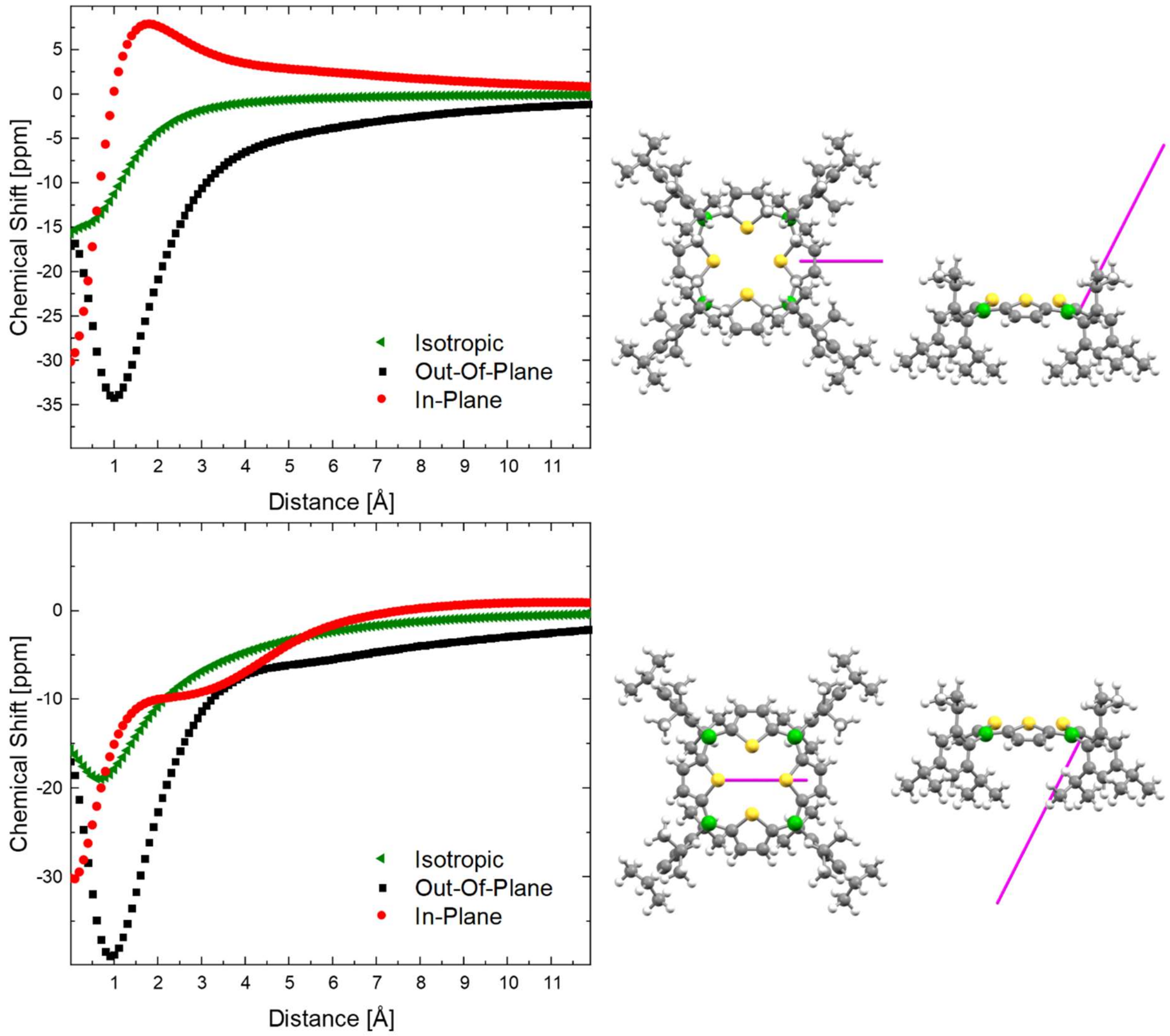

Figure S71: **Top**: NICS-Scan of the thiophene moiety of $\mathbf{4}^{2-}$ going outwards. The values were obtained *via* a dummy atom (purple) going from the middle of the thiophene (Z = 0 Å) perpendicular to its-plane. **Bottom**: NICS-Scan of the thiophene moiety of $\mathbf{4}^{2-}$ going inwards. The values were obtained *via* a dummy atom (purple) going from the middle of the thiophene (Z = 0 Å) perpendicular to its-plane

Table S5: Results from TD-DFT calculations for the compounds for **1**. Transitions of minor contribution in grey.

State	λ / nm	Oscillator strength *f*	Orbital contributions	$\vert c\vert^2$ / %
S_1	304.2	0.0010	HOMO → LUMO	92.7
S_2	293.3	0.4136	HOMO−2 → LUMO<br>HOMO−1 → LUMO	59.5<br>35.2
S_3	279.0	0.0223	HOMO−1 → LUMO<br>HOMO−2 → LUMO	60.2<br>34.5
S_4	259.3	0.1064	HOMO−3 → LUMO	93.5
S_5	251.5	0.0633	HOMO−4 → LUMO<br>HOMO−5 → LUMO	70.5<br>24.2
S_6	245.3	0.2969	HOMO−5 → LUMO<br>HOMO−4 → LUMO	68.5<br>25.3

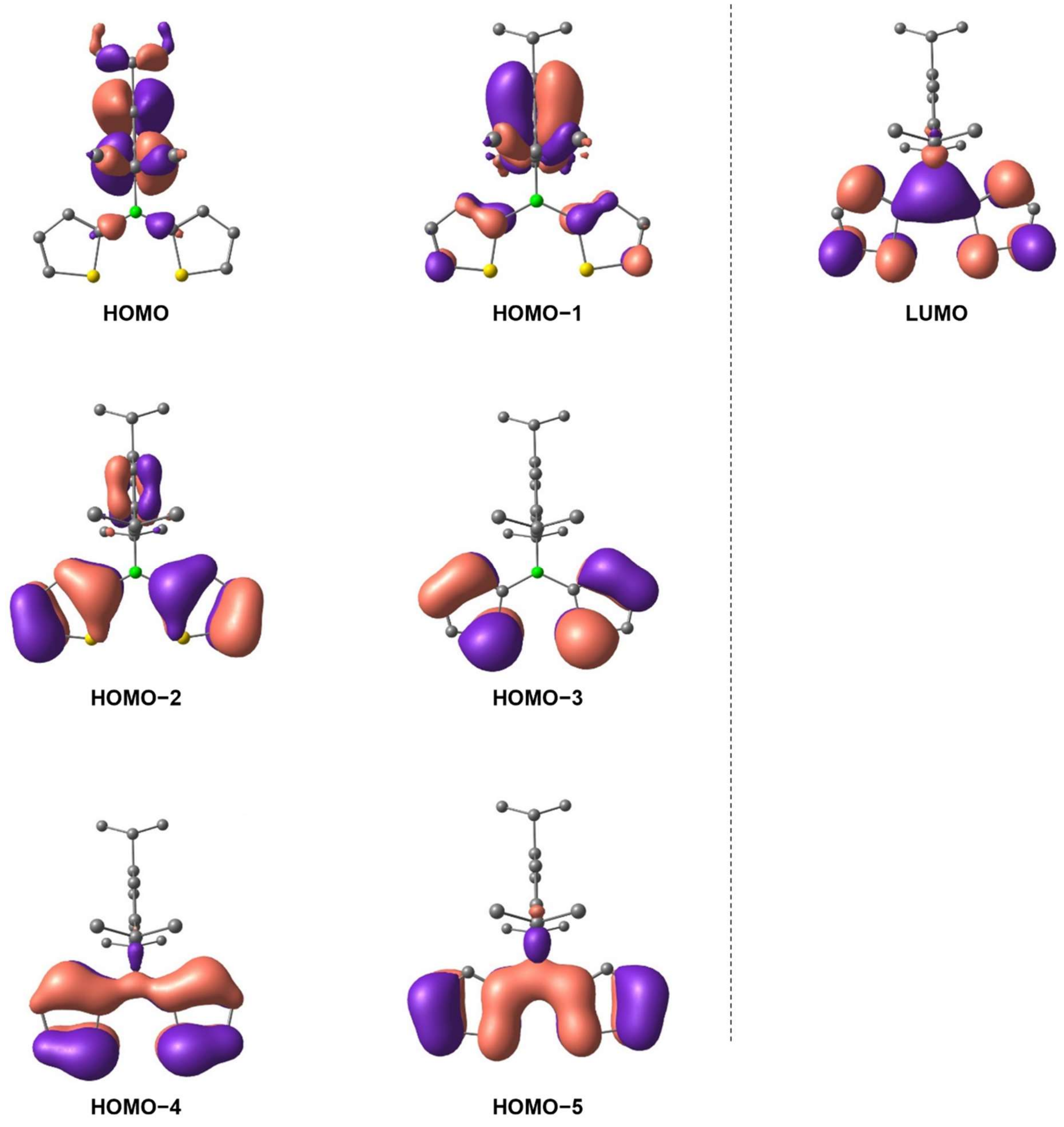

Figure S72: Calculated frontier orbitals (isovalue 0.03 a.u.) of **1** (ω_TB97X-D3/def2-SVP, CPCM(THF), ω_T = 0.16).

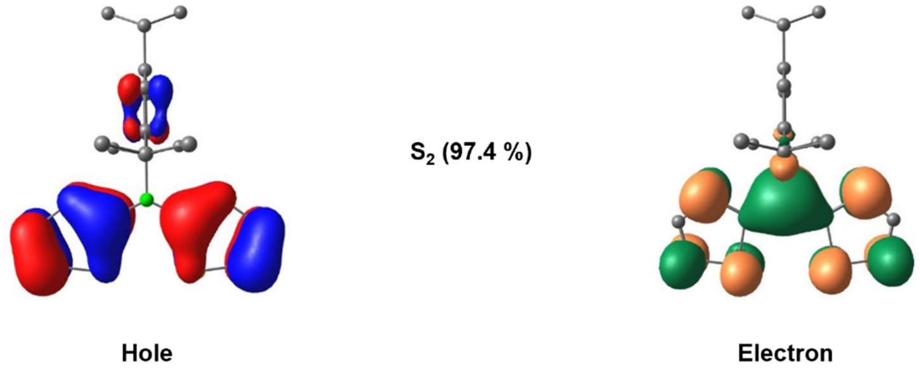

Figure S73: Calculated natural transition orbitals (NTOs) (isovalue 0.03 a.u.) of **1** (ω_TB97X-D3/def2-SVP, CPCM(THF), ω_T = 0.16) (excitation from “Hole” to “Electron”).

Table S6: Results from TD-DFT calculations for the compounds for **2**. Transitions of minor contribution in grey.

State	λ / nm	Oscillator strength *f*	Orbital contributions	$\|c\|^2$ / %
S_1	334.8	0.0388	HOMO → LUMO<br>HOMO−1 → LUMO	48.3<br>37.5
**S_2**	332.9	0.3977	**HOMO−1 → LUMO**<br>**HOMO → LUMO**<br>**HOMO−4 → LUMO**	40.7<br>28.4<br>18.0
**S_3**	327.9	0.3582	**HOMO−2 → LUMO**<br>**HOMO−4 → LUMO**	49.4<br>26.1
S_4	312.2	0.0198	HOMO−3 → LUMO<br>HOMO−2 → LUMO<br>HOMO−4 → LUMO	48.7<br>20.3<br>16.9
S_5	307.2	0.0206	HOMO−3 → LUMO<br>HOMO−4 → LUMO<br>HOMO−2 → LUMO	37.4<br>28.0<br>15.7
S_6	288.7	0.0267	HOMO−6 → LUMO<br>HOMO−5 → LUMO	71.7<br>11.8
**S_7**	281.2	0.0925	**HOMO−5 → LUMO**<br>**HOMO−6 → LUMO**<br>**HOMO−4 → LUMO+1**	58.6<br>15.1<br>11.6
S_8	270.5	0.0454	HOMO−7 → LUMO	73.7
S_9	269.4	0.0355	HOMO−8 → LUMO<br>HOMO−8 → LUMO+1	76.0<br>11.7
**S_{10}**	260.5	0.3469	**HOMO−9 → LUMO**<br>**HOMO−5 → LUMO+1**	81.5<br>11.5

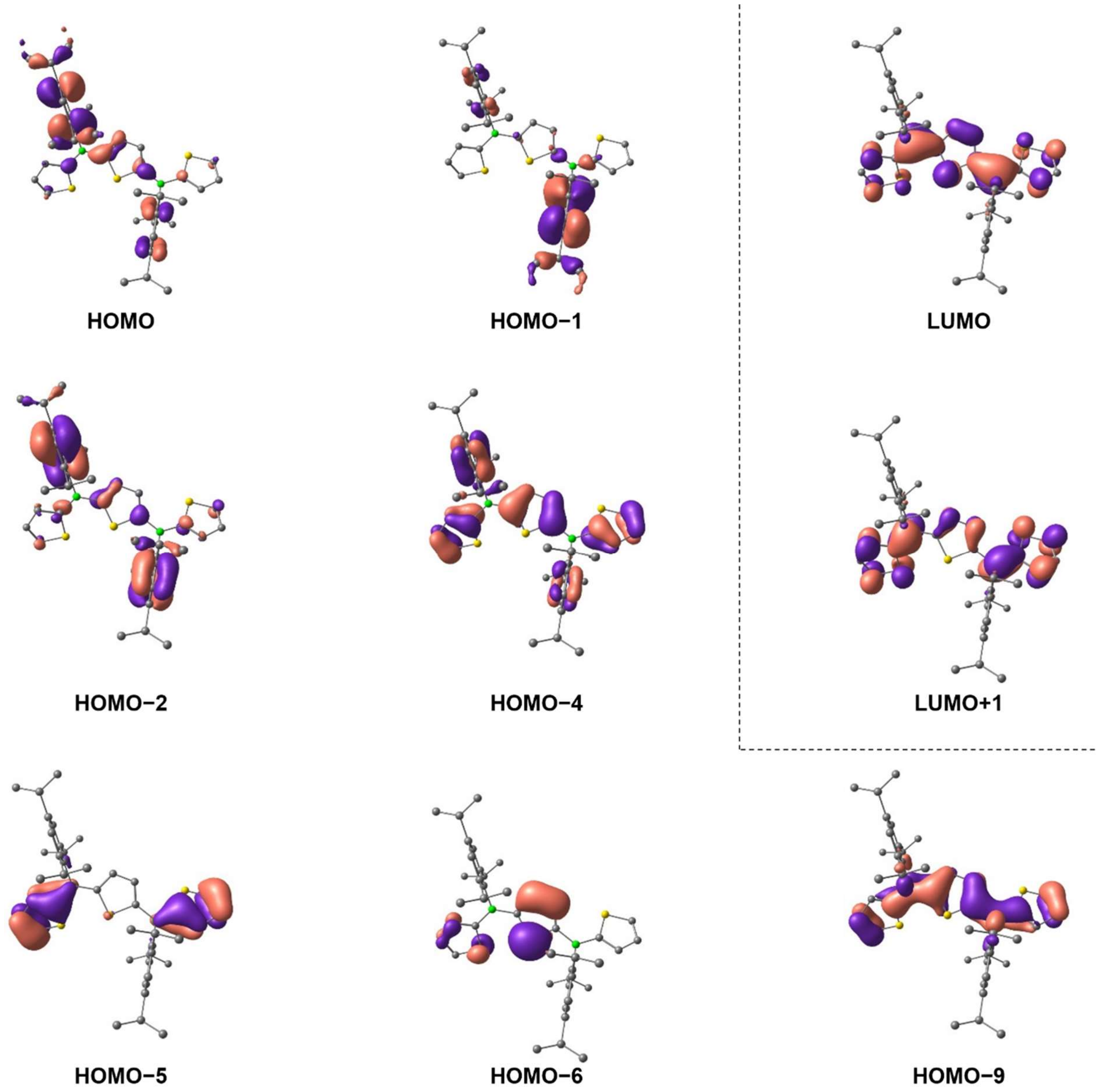

Figure S74: Calculated frontier orbitals (isovalue 0.03 a.u.) of **2** (ω_TB97X-D3/def2-SVP, CPCM(THF), ω_T = 0.13).

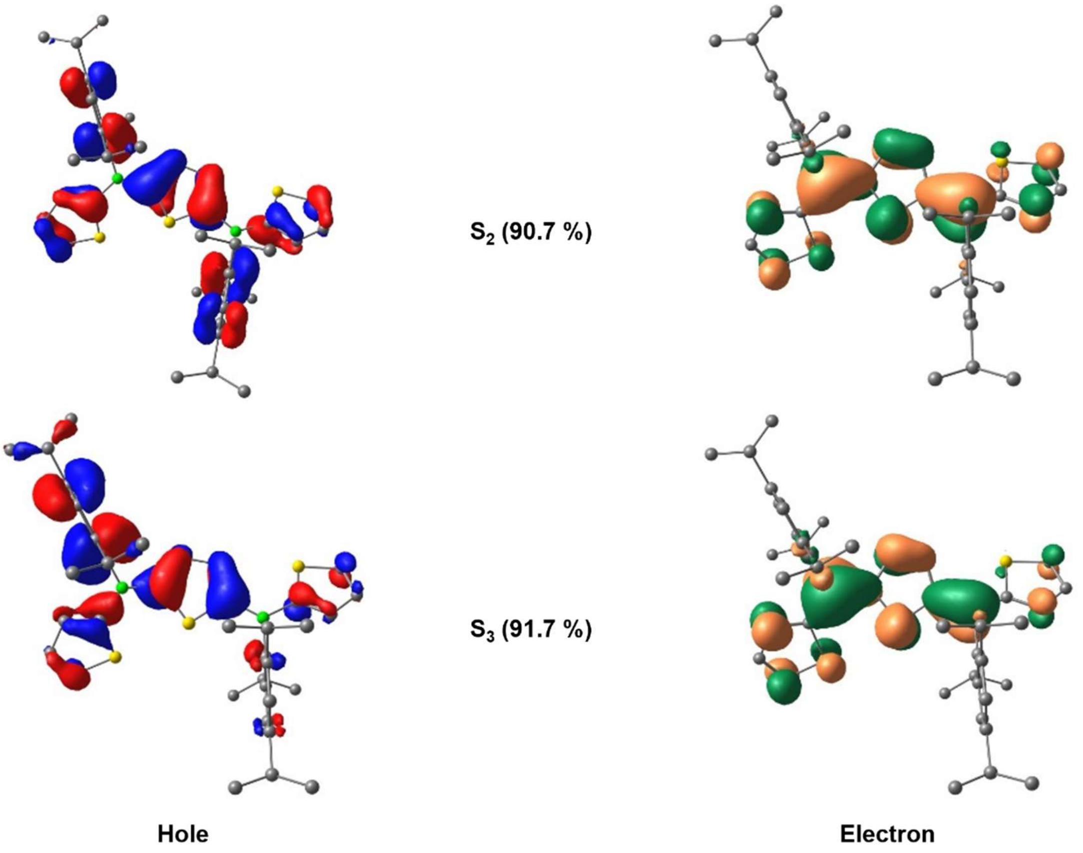

Figure S75: Calculated natural transition orbitals (NTOs) (isovalue 0.03 a.u.) of **2** (ω_TB97X-D3/def2-SVP, CPCM(THF), ω_T = 0.13) (excitation from "Hole" to "Electron").

Table S7. Results from TD-DFT calculations for the compounds for **3**. Transitions of minor contribution in grey.

State	λ / nm	Oscillator strength *f*	Orbital contributions	|c|^2 / %
**S$_1$**	355.6	0.4565	**HOMO → LUMO**<br>**HOMO−4 → LUMO**<br>**HOMO−6 → LUMO**	27.2<br>28.3<br>16.7
S$_2$	351.1	0.1613	HOMO−2 → LUMO<br>HOMO−3 → LUMO<br>HOMO → LUMO	53.0<br>14.5<br>10.3
S$_3$	350.6	0.1194	HOMO−1 → LUMO<br>HOMO → LUMO<br>HOMO → LUMO+1<br>HOMO−2 → LUMO	37.4<br>18.2<br>10.7<br>10.2
S$_4$	347.9	0.0897	HOMO−1 → LUMO<br>HOMO−2 → LUMO<br>HOMO−1 → LUMO+1<br>HOMO−3 → LUMO	28.3<br>23.5<br>20.3<br>11.3
S$_5$	334.1	0.0019	HOMO−3 → LUMO<br>HOMO−4 → LUMO<br>HOMO → LUMO<br>HOMO−3 → LUMO+1	33.1<br>27.8<br>13.2<br>12.9
S$_6$	327.2	0.0077	HOMO−5 → LUMO	76.4
S$_7$	326.8	0.0245	HOMO−6 → LUMO<br>HOMO−4 → LUMO	50.3<br>20.6
**S$_8$**	310.4	0.3463	**HOMO−7 → LUMO**<br>**HOMO−6 → LUMO+1**	57.9<br>17.9
S$_9$	300.3	0.0489	HOMO−8 → LUMO<br>HOMO−10 → LUMO	65.5<br>13.1
S$_{10}$	298.6	0.0769	HOMO−9 → LUMO<br>HOMO−8 → LUMO<br>HOMO−11 → LUMO<br>HOMO−12 → LUMO	34.4<br>16.6<br>13.6<br>13.0
**S$_{11}$**	281.4	0.2182	**HOMO−7 → LUMO+1**<br>**HOMO−11 → LUMO**<br>**HOMO−12 → LUMO**	25.7<br>26.9<br>17.3
S$_{12}$	277.1	0.0322	HOMO−10 → LUMO<br>HOMO−10 → LUMO+1<br>HOMO−9 → LUMO	37.5<br>15.5<br>10.4
S$_{13}$	275.7	0.0430	HOMO−12 → LUMO<br>HOMO−9 → LUMO<br>HOMO−11 → LUMO<br>HOMO−12 → LUMO+1<br>HOMO−9 → LUMO+1	21.6<br>17.8<br>16.5<br>12.7<br>10.7

S_{14}	273.8	0.0015	**HOMO → LUMO+1**<br>**HOMO→ LUMO**	40.4<br>17.6
S_{15}	272.7	0.0257	**HOMO−1 → LUMO+1**<br>**HOMO−1 → LUMO**<br>**HOMO → LUMO+1**	29.2<br>22.0<br>13.1
S_{16}	271	0.0018	**HOMO−1 → LUMO+1**<br>**HOMO−3 → LUMO+1**<br>**HOMO−13 → LUMO**	20.0<br>18.5<br>11.6
S_{17}	267.6	0.0026	**HOMO−4 → LUMO+1**<br>**HOMO−3 → LUMO+1**<br>**HOMO−3 → LUMO**	28.5<br>25.4<br>11.3
**S_{18}**	265.2	0.2114	**HOMO−13 → LUMO**<br>**HOMO−2 → LUMO+1**	30.9<br>25.6

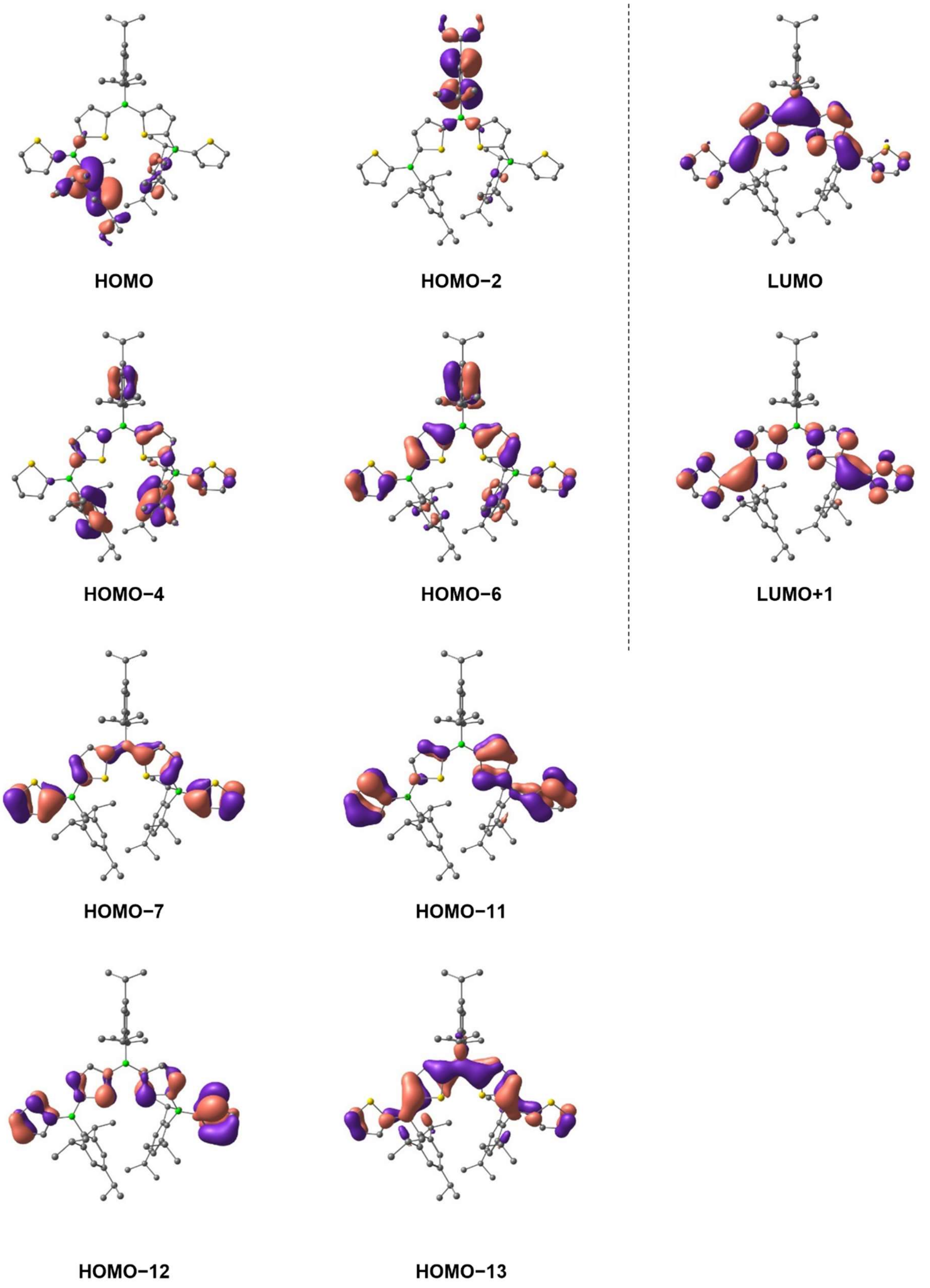

Figure S76: Calculated frontier orbitals (isovalue 0.03 a.u.) of **3** (ω_TB97X-D3/def2-SVP, CPCM(THF), ω_T = 0.12).

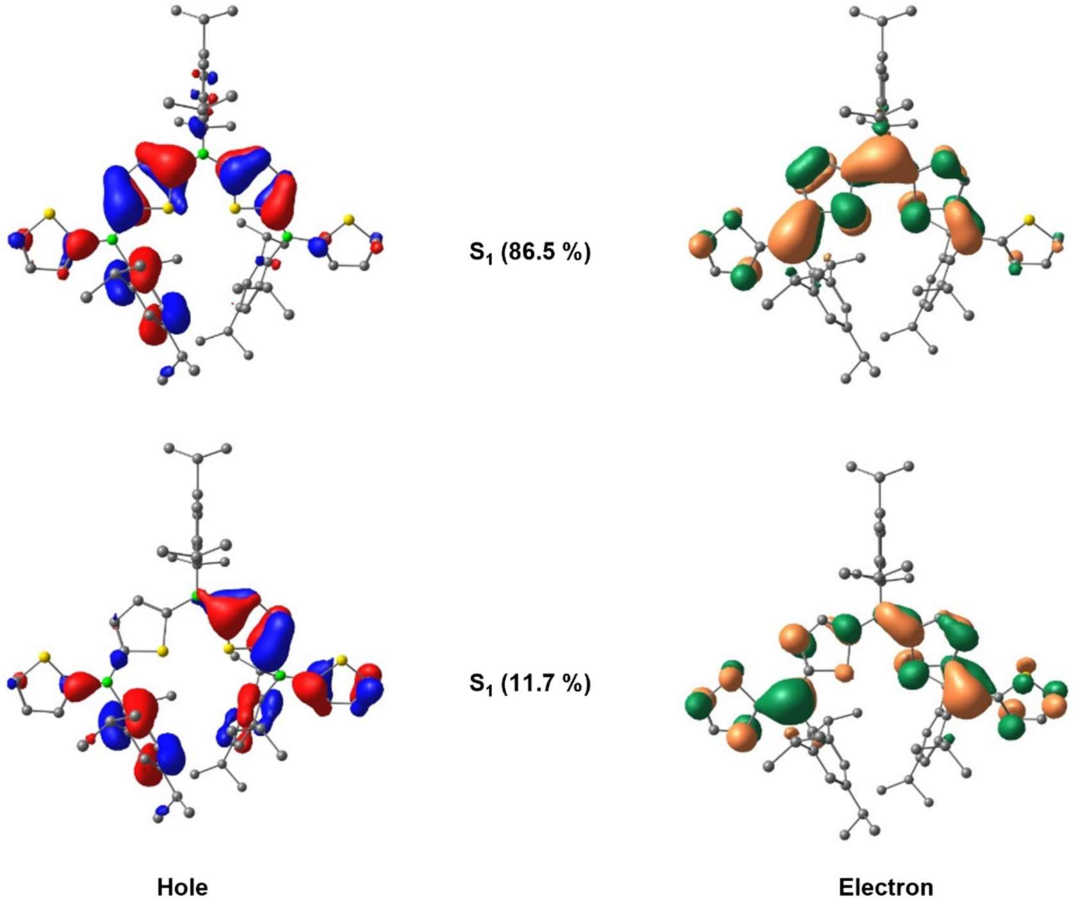

Figure S77: Calculated natural transition orbitals (NTOs) (isovalue 0.03 a.u.) of **3** (ω_TB97X-D3/def2-SVP, CPCM(THF), ω_T = 0.12) (excitation from “Hole” to “Electron”).

Table S8. Results from TD-DFT calculations for the compounds for **4**. Transitions of minor contribution in grey.

State	λ / nm	Oscillator strength *f*	Orbital contributions	$\|c\|^2$ / %
S1	379.9	0.0000	HOMO−1 → LUMO	62.0
			HOMO−8 → LUMO	17.3
S_2	346.4	0.0000	HOMO → LUMO	59.1
			HOMO−2 → LUMO+1	13.2
			HOMO−3 → LUMO+2	12.8
S_3	340.7	0.0683	HOMO−2 → LUMO	61.1
			HOMO → LUMO+1	14.9
			HOMO−4 → LUMO+2	10.6
S_4	340.7	0.0849	HOMO−3 → LUMO	61.4
			HOMO → LUMO+2	14.6
			HOMO−4 → LUMO+1	11.1
S_5	337.9	0.0002	HOMO−4 → LUMO	62.8
			HOMO−3 → LUMO+1	10.4
S_6	324.2	0.0002	HOMO−9 → LUMO	77.7
**S_7**	322.5	0.5595	**HOMO−6 → LUMO**	39.7
			**HOMO−10 → LUMO**	26.1
			**HOMO−1 → LUMO+1**	19.3
**S_8**	322.0	0.3884	**HOMO−5 → LUMO**	49.3
			**HOMO−11 → LUMO**	18.1
			**HOMO−1 → LUMO+2**	16.5
S_9	318.7	0.0068	HOMO−7 → LUMO	62.2
			HOMO−5 → LUMO+1	14.3
			HOMO−6 → LUMO+2	13.9
S_{10}	318.6	0.0000	HOMO−8 → LUMO	55.6
			HOMO−6 → LUMO+1	13.3
			HOMO−5 → LUMO+2	13.3
			HOMO−1 → LUMO	10.4
**S_{11}**	315.6	0.4030	**HOMO−6 → LUMO**	26.0
			**HOMO−10 → LUMO**	37.2
			**HOMO−8 → LUMO+1**	17.3
**S_{12}**	314.6	0.5488	**HOMO−5 → LUMO**	17.2
			**HOMO−11 → LUMO**	45.4
			**HOMO−8 → LUMO+2**	16.5

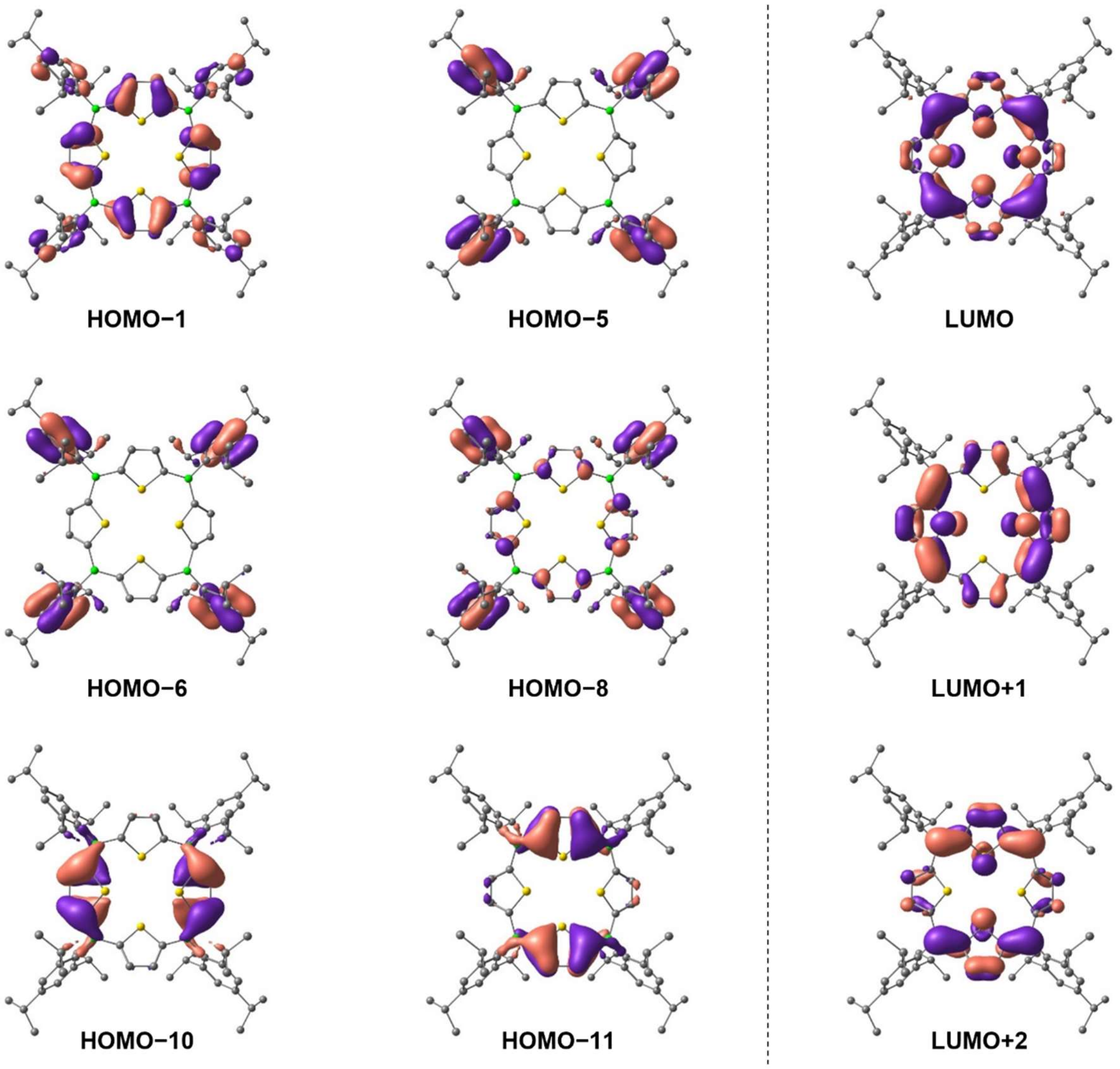

Figure S78: Calculated frontier orbitals (isovalue 0.03 a.u.) of **4** (ω_TB97X-D3/def2-SVP, CPCM(THF), ω_T = 0.125).

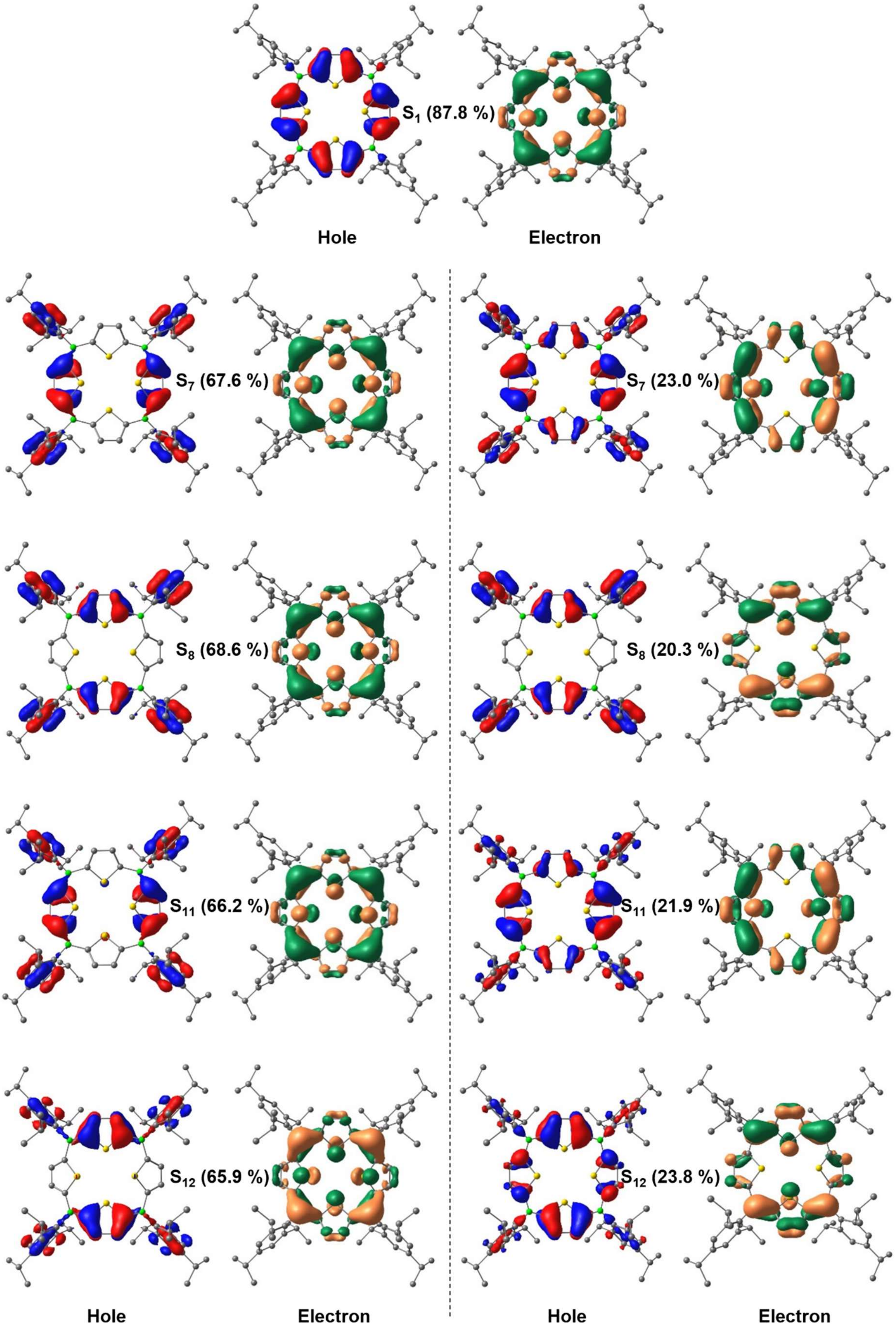

Figure S79: Calculated natural transition orbitals (NTOs) (isovalue 0.03 a.u.) of **4** (ω_TB97X-D3/def2-SVP, CPCM(THF), ω_T = 0.125) (excitation from "Hole" to "Electron").

Table S9. Results from TD-DFT calculations for the compounds for **4**$^{•-}$. Transitions of minor contribution in grey.

State	λ / nm	Oscillator strength *f*	Orbital contributions	|c|² / %
**D_1**	1218.3	0.2959	**SOMOa → LUMOa**	98.6
**D_2**	1183.0	0.300	**SOMOa → LUMO+1a**	98.5
D_3	486.6	0.0000	HOMOb → SOMOb	81.8
D_4	442.4	0.0000	SOMOa → LUMO+3a	86.3
D_5	438.2	0.0242	HOMOa → LUMO+1a<br>HOMO−10b → SOMOb<br>HOMOb → LUMO+1b	38.3<br>22.7<br>15.9
D_6	434.0	0.0275	HOMOa → LUMO+2a<br>HOMO−11b → SOMOb<br>HOMOb → LUMO+2b	38.1<br>28.2<br>15.4
D_7	413.2	0.0000	HOMO−4b → SOMOb<br>SOMOa → LUMO3a	49.1<br>27.4
D_8	412	0.0000	SOMOa → LUMO3a<br>HOMO−4b → SOMOb	55.3<br>23.3
D_9	382.8	0.0170	HOMO−11b → SOMOb<br>HOMO−12b → SOMOb<br>HOMO−4b → LUMOb	28.1<br>22.7<br>11.1
D_{10}	382.5	0.0266	HOMO−10b → SOMOb<br>HOMO−13b → SOMOb	31.5<br>20.1
D_{11}	382.4	0.0000	HOMO−15b → SOMOb<br>HOMO−10a → LUMOa<br>HOMO−11a → LUMO+1a	20.2<br>12.9<br>11.8
D_{12}	370.9	0.0000	SOMOa → LUMO+12a	89.5
D_{13}	364.7	0.0000	HOMO−1b → SOMOb	81.7
D_{14}	356.9	0.0185	HOMO−2b → SOMOb	70.0
D_{15}	356.4	0.0002	HOMO−3b → SOMOb	77.7
D_{16}	354.3	0.0012	HOMO−14b → SOMOb<br>HOMO−10a → LUMO+1a<br>HOMO−11a → LUMOa	36.0<br>15.0<br>12.7
D_{17}	347.8	0.0001	HOMO−5b → SOMOb	74.7
**D_{18}**	347.7	0.4294	**HOMOa → LUMO+1a**<br>**HOMO−5a → LUMOa**<br>**HOMO−11b → SOMOb**	39.4<br>11.3<br>17.0

			**HOMOa → LUMOa**	34.9
**D_{19}**	347.4	0.4271	**HOMO−6b → SOMOb**	11.5
			**HOMO−10b → SOMOb**	9.8

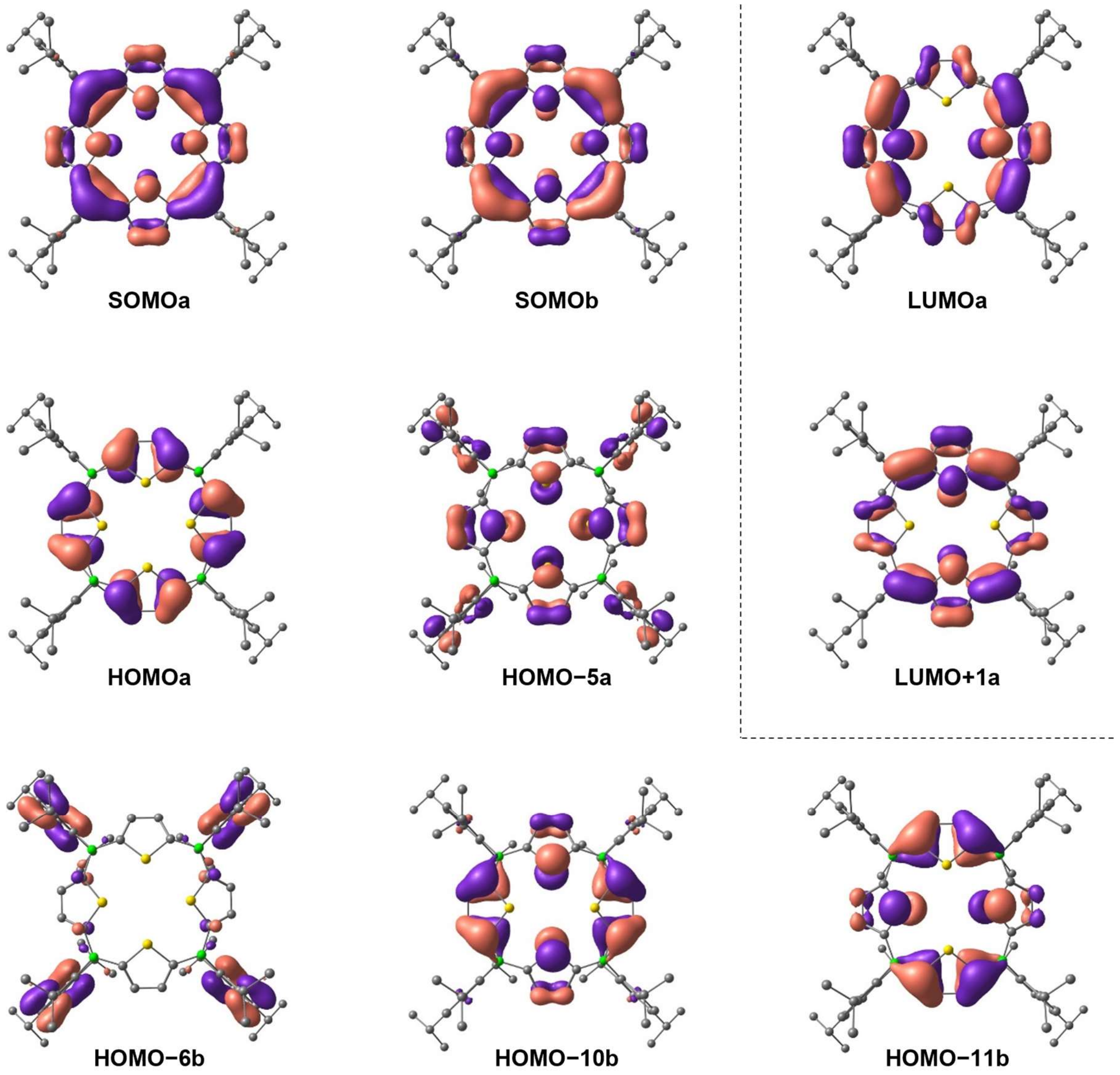

Figure S80: Calculated frontier orbitals (isovalue 0.03 a.u.) of $\mathbf{4}^{\bullet-}$ (Uω_TB97X-D3/def2-SVP, CPCM(THF), ω_T = 0.1).

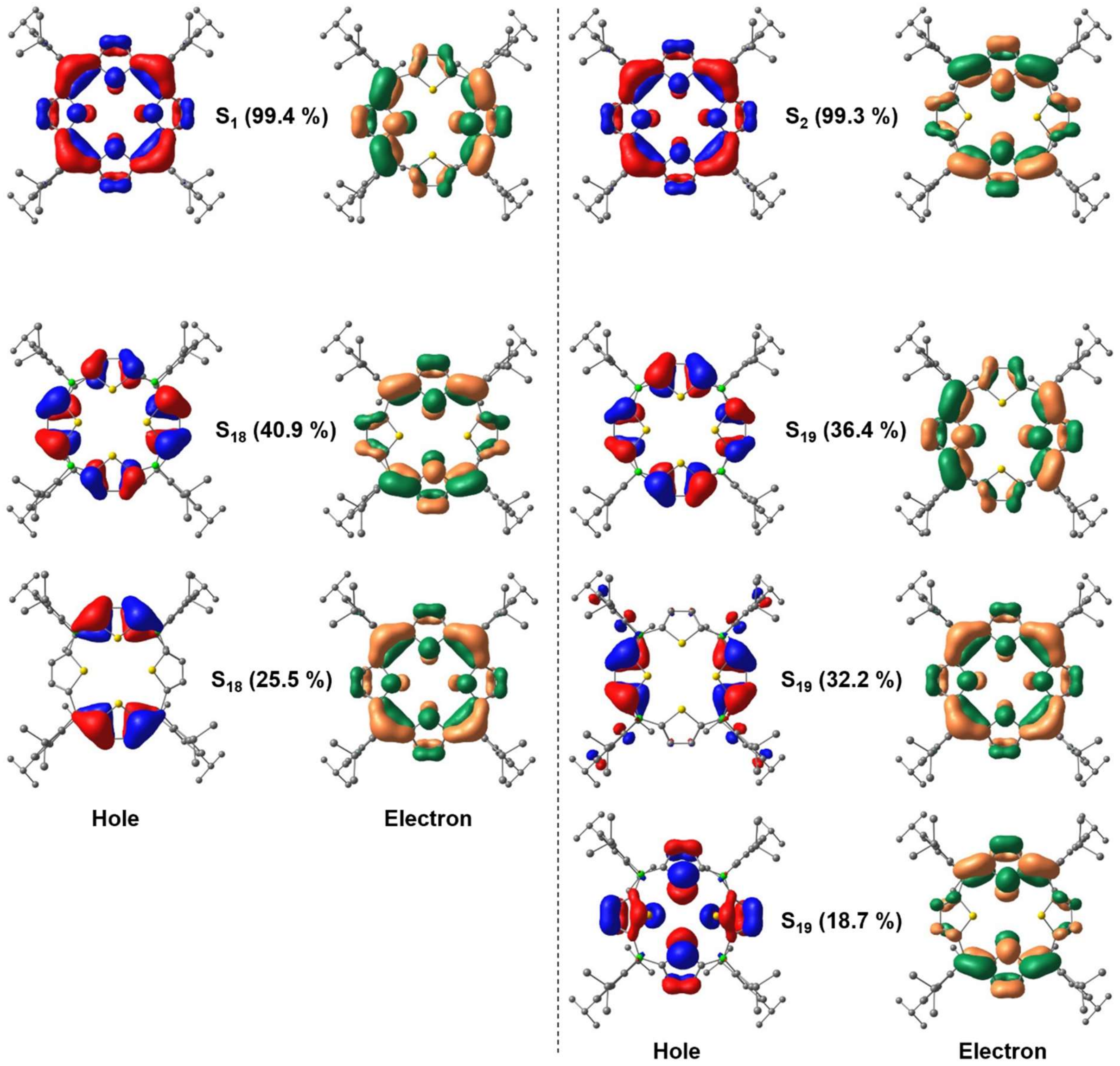

Figure S81: Calculated natural transition orbitals (NTOs) (isovalue 0.03 a.u.) of **4**$^{\bullet-}$ (Uω_TB97X-D3/def2-SVP, CPCM(THF), ω_T = 0.1) (excitation from "Hole" to "Electron").

Table S10: Results from TD-DFT calculations for the compounds for **4**$^{2-}$. Transitions of minor contribution in grey.

State	λ / nm	Oscillator strength *f*	Orbital contributions	$\lvert c\rvert^2$ / %
S_1	833.7	0.6135	HOMO → LUMO	98.0
S_2	827.4	0.6024	HOMO → LUMO+1	97.9
S_3	398.9	0.0000	HOMO → LUMO+9<br>HOMO → LUMO+3<br>HOMO → LUMO+12	58.3<br>24.2<br>16.0
S_4	396.8	0.0000	HOMO → LUMO+2<br>HOMO → LUMO+11	71.7<br>24.2
S_5	379.3	0.0067	HOMO → LUMO+4<br>HOMO → LUMO+8	86.5<br>11.9
S_6	378	0.0000	HOMO → LUMO+3<br>HOMO → LUMO+12<br>HOMO → LUMO+9	72.6<br>14.2<br>12.2
S_7	377.7	0.0002	HOMO → LUMO+5	88.1
S_8	375.3	0.0000	HOMO → LUMO+6	96.5
S_9	375.2	0.0006	HOMO → LUMO+7<br>HOMO → LUMO+5	87.9<br>10.1
S_{10}	374.5	0.0003	HOMO → LUMO+8<br>HOMO → LUMO+4	85.9<br>12.4
S_{11}	369.3	0.0000	HOMO → LUMO+11<br>HOMO → LUMO+2	72.2<br>25.0
S_{12}	367.6	0.0000	HOMO → LUMO+12<br>HOMO → LUMO+9	68.8<br>27.7
S_{13}	360.5	0.0000	HOMO → LUMO+10	93.2
S_{14}	349.1	0.6707	HOMO−1 → LUMO	93.2
S_{15}	348.0	0.6900	HOMO−1 → LUMO+1	92.0

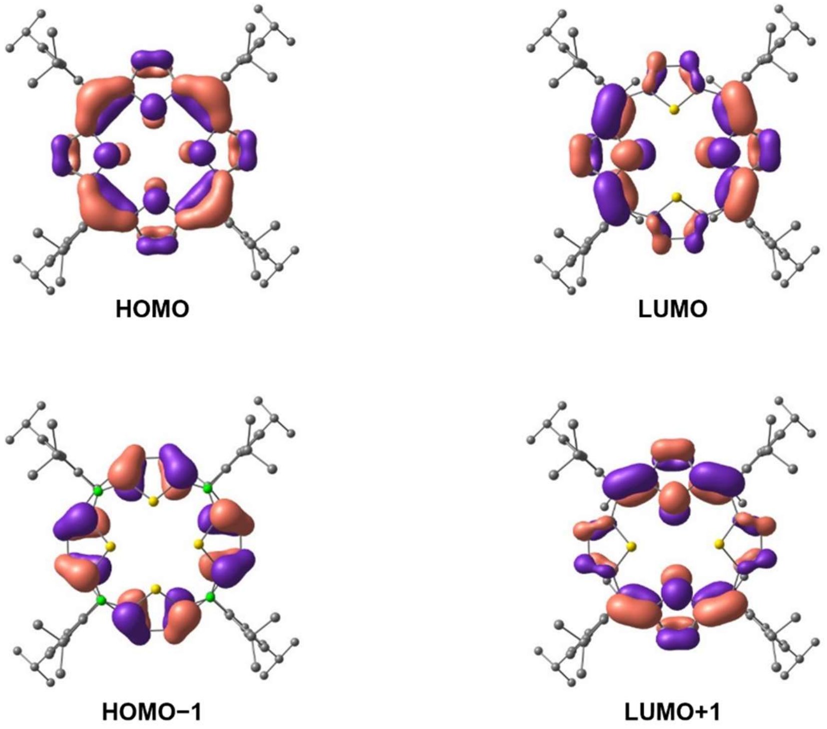

Figure S82: Calculated frontier orbitals (isovalue 0.03 a.u.) of **4**$^{2-}$ (ω_TB97X-D3/def2-SVP, CPCM(THF), ω_T = 0.1).

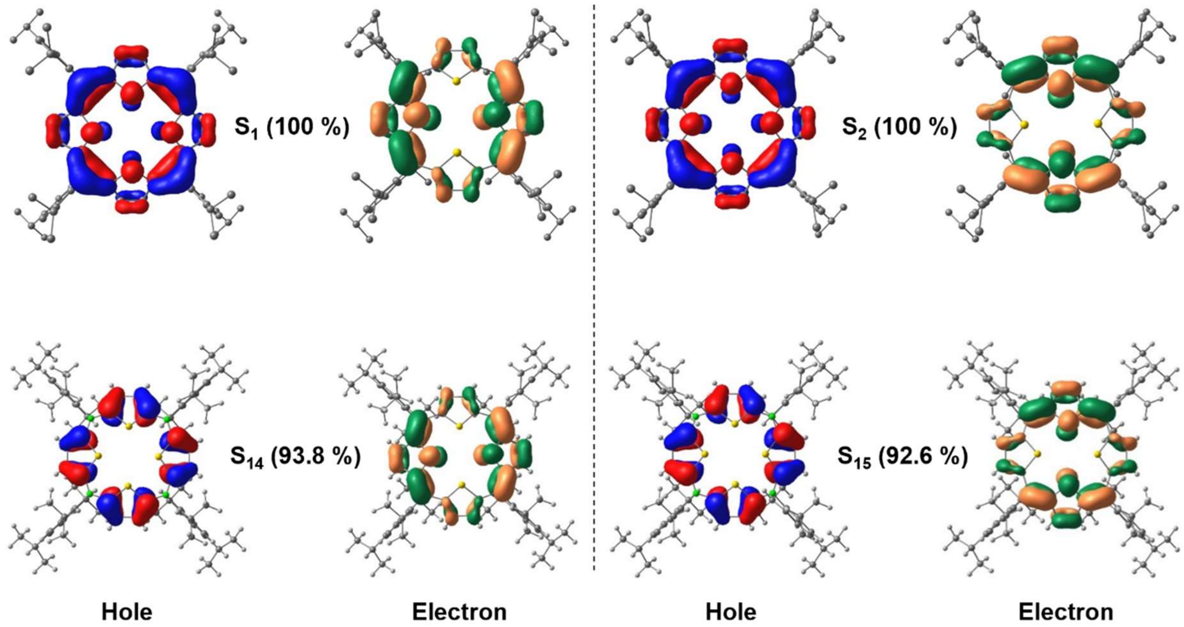

Figure S83: Calculated natural transition orbitals (NTOs) (isovalue 0.03 a.u.) of **4**$^{2-}$ (ω_TB97X-D3/def2-SVP, CPCM(THF), ω_T = 0.1) (excitation from "Hole" to "Electron").

Table S11: Results from TD-DFT calculations for the compounds for **TPP**.

State	λ / nm	Oscillator strength *f*	Orbital contributions	$\|c\|^2$ / %
S_1	575.6	0.0331	HOMO → LUMO<br>HOMO-1 → LUMO+1	65.7<br>32.4
S_2	533.9	0.0411	HOMO → LUMO+1<br>HOMO-1 → LUMO	62.6<br>36.3
S_3	394.3	1.5766	HOMO → LUMO<br>HOMO-1 → LUMO+1	28.7<br>63.5
S_4	388.1	1.8728	HOMO → LUMO+1<br>HOMO-1 → LUMO	35.6<br>61.8

As already described in the literature, we see that the Q and B bands are described by excitations of a mixture of the four orbitals HOMO-1, HOMO, LUMO and LUMO+1. Because of the fact that for example an excitation from HOMO to LUMO or HOMO-1 to LUMO+1 are nearly equal in energy (D_{2h} point group), we can make two degenerate linear combinations to generate the minus (−) and plus (+) states which are of B_{3u} symmetry. The transition dipole moment vectors (y coordinate for D_{2h}) are parallel and have similar dimensions which leads to a pseudoparity forbidden antiparallel B_{3u}^{-} (Q-band) transition and a parallel B_{3u}^{+} (B-band) transition. The same can be applied for the B_{2u} transitions (HOMO → LUMO+1 and HOMO-1 → LUMO). This leads to significantly lower oscillator strengths of the Q-bands.[39] As described, our bowl-shaped dianionic species **4**$^{2-}$ (C_{4v} symmetry) is distorted from the classical porphyrin. This leads to a large energetical splitting of the HOMO and HOMO-1 orbitals (Figure S84). This can be ascribed to the fact that the boron atoms, that have a higher atom energy[40] than carbon atoms in TPP, contribute to the HOMO while the HOMO-1 has a nodal plane at each boron center. This leads to a larger elevation of the HOMO in **4**$^{2-}$ in comparison to TPP. Thus, the before described mixing of these states is less likely.

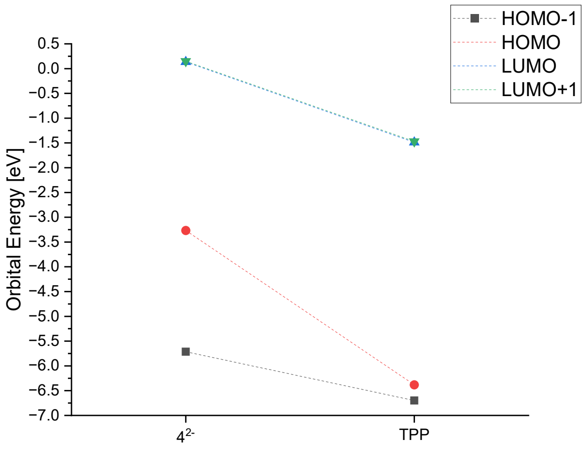

Figure S84: Calculated energies of the HOMO-1 to LUMO+1 orbitals of **4^{2-}** and **TPP** (ω_TB97X-D3/def2-SVP, CPCM(THF), ω_T = 0.1 and 0.12).

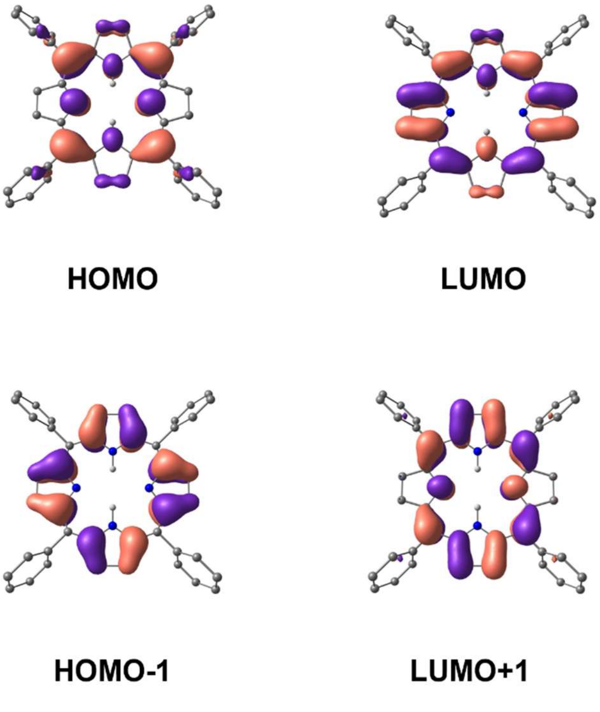

Figure S85: Calculated frontier orbitals (isovalue 0.03 a.u.) of **TPP** (ω_TB97X-D3/def2-SVP, CPCM(THF), ω_T = 0.12).

Table S12: Orbital energies for **4**$^{2-}$ and **TPP** (ω_TB97X-D3/def2-SVP, CPCM(THF)) in eV.

Compound	HOMO-1	HOMO	LUMO	LUMO+1
**4**$^{2-}$	-5.71	-3.27	0.13	0.15
**TPP**	-6.70	-6.38	-1.49	-1.47

Table S13: Gibbs free energies for the fluoride adducts in kcal/mol.

Compound	**4 + *x* F**[a]	**4 + *x* TBAF**[b]
**4-1F**	-19.2	-13.5
**4-2F**	-34.5	-23.2
**4-3F**	-26.2	-9.3
**4-4F**	-17.4	5.2

[a] energies with respect to molecule **4** and “x” naked fluorine anions and TBA (tert-butyl-ammonium) cations.

[b] energies with respect to molecule **4** and “x” naked fluorine anions and “x” TBAF (tert-butyl-ammonium fluoride) molecules.

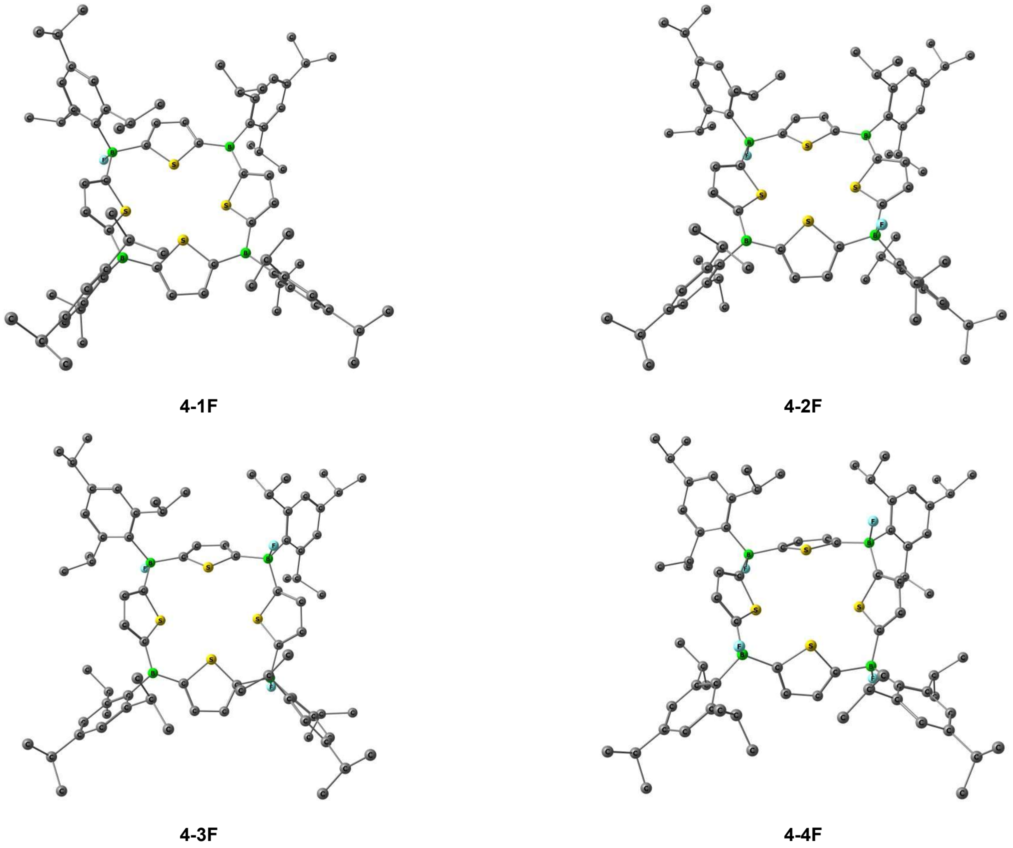

Figure S86: Calculated equilibrium geometries of the used fluoride adduct isomers.

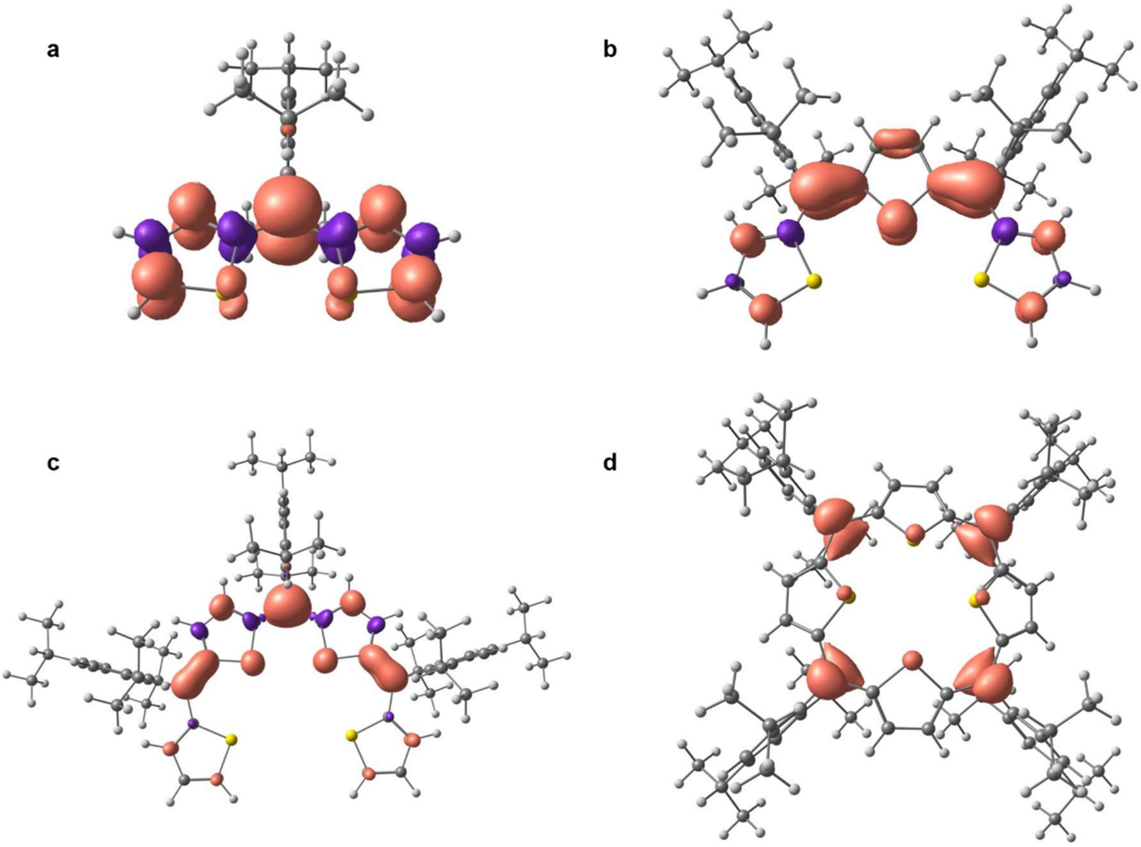

Figure S87: Calculated spin-density-distributions of compound **1**$^{\bullet-}$ (**a**), **2**$^{\bullet-}$ (**b**), **3**$^{\bullet-}$ (**c**) and **4**$^{\bullet-}$ (d).

Supplementary References

1. Ruhlandt-Senge, K., Ellison, J. J., Wehmschulte, R. J., Pauer, F. & Power, P. P. Isolation and structural characterization of unsolvated lithium aryls. *J. Am. Chem. Soc.* **115**, 11353-11357 (1993).
2. Lik, A., Fritze, L., Müller, L. & Helten, H. Catalytic B–C Coupling by Si/B Exchange: A Versatile Route to π-Conjugated Organoborane Molecules, Oligomers, and Polymers. *J. Am. Chem. Soc.* **139**, 5692-5695 (2017).
3. Weber, L., Werner, V., Domke, I., Stammler, H.-G. & Neumann, B. 1,3,2-Diazaborolyl-functionalized thiophenes and dithiophenes: synthesis, structure, electrochemistry and luminescence. *Dalton Trans.*, 3777-3784 (2006).
4. Chorbacher, J., Maier, M., Klopf, J., Fest, M. & Helten, H. Poly(thiophene iminoborane): A Poly(thiophene vinylene) (PTV) Analogue with a Fully B=N-Doped Backbone. *Macromol. Rapid Commun.* **44**, e2300278 (2023).
5. Pelter, A., Smith, K., Buss, D. & Jin, Z. Hindered organoboron groups in organic synthesis. 17 [1]. Synthesis of 2,4,6-triisopropylphenylborane ($TripBH_2$)$_2$, a useful alternative to thexylborane. *Heteroat. Chem.* **3**, 275-277 (2004).
6. Sheldrick, G. M. Crystal structure refinement with SHELXL. *Acta Crystallogr. Sect. C: Cryst. Struct. Commun.* **71**, 3-8 (2015).
7. Sheldrick, G. M. A short history of SHELX. *Acta Crystallogr. Sect. A: Found. Crystallogr.* **64**, 112-122 (2008).
8. Hübschle, C. B., Sheldrick, G. M. & Dittrich, B. ShelXle: a Qt graphical user interface for SHELXL. *J. Appl. Crystallogr.* **44**, 1281-1284 (2011).
9. Stoll, S. & Schweiger, A. EasySpin, a comprehensive software package for spectral simulation and analysis in EPR. *Journal of Magnetic Resonance* **178**, 42-55 (2006).
10. Lik, A. et al. From Monodisperse Thienyl- and Furylborane Oligomers to Polymers: Modulating the Optical Properties through the Hetarene Ratio. *Chem. Eur. J.* **24**, 11961-11972 (2018).
11. M. J. Frisch, G. W. Trucks, H. B. Schlegel, G. E. Scuseria, M. A. Robb, J. R. Cheeseman, G. Scalmani, V. Barone, G. A. Petersson, H. Nakatsuji, M. Caricato X. Li, A. V. Marenich, J. Bloino, B. G. Janesko, R. Gomperts, B. Mennucci, H. P. Hratchian, J. V. Ortiz, A. F. Izmaylov, J. L. Sonnenberg, D. Williams-Young, F. Lipparini F. Ding, J. Goings F. Egidi, B. Peng, A. Petrone, T. Henderson, D. Ranasinghe, V. G. Zakrzewski, J. Gao, N. Rega, G. Zheng, W. Liang, M. Hada, M. Ehara, K. Toyota, R. Fukuda, J. Hasegawa, M. Ishida, T. Nakajima, Y. Honda, O. Kitao, H. Nakai, T. Vreven, K. Throssell, Jr. J. A. Montgomery, J. E. Peralta, F. Ogliaro, M. J. Bearpark, J. J. Heyd, E. N. Brothers, K. N. Kudin, V. N. Staroverov, T. A. Keith, R. Kobayashi, J. Normand, K. Raghavachari, A. P. Rendell, J. C. Burant, S. S. Iyengar, J. Tomasi, M. Cossi, J. M. Millam, M. Klene, C. Adamo, R. Cammi, J. W. Ochterski, R. L. Martin, K. Morokuma, O. Farkas, J. B. Foresman, D. J. Fox, Gaussian, Gaussian 16 Revision C.01, Inc., Wallingford CT (2016).
12. Chai, J.-D. & Head-Gordon, M. Long-range corrected hybrid density functionals with damped atom–atom dispersion corrections. *Phys. Chem. Chem. Phys.* **10**, 6615-6620 (2008).
13. Clark, T., Chandrasekhar, J., Spitznagel, G. W. & Schleyer, P. V. R. Efficient diffuse function-augmented basis sets for anion calculations. III. The 3-21+G basis set for first-row elements, Li–F. *J. Comp. Chem.* **4**, 294-301 (1983).

14. Dill, J. D. & Pople, J. A. Self-consistent molecular orbital methods. XV. Extended Gaussian-type basis sets for lithium, beryllium, and boron. *J. Chem. Phys.* **62**, 2921-2923 (1975).
15. Ditchfield, R., Hehre, W. J. & Pople, J. A. Molecular Orbital Theory of the Electronic Structure of Organic Compounds. VII. A Systematic Study of Energies, Conformations, and Bond Interactions. *J. Chem. Phys.* **54**, 724-728 (1971).
16. Francl, M. M. et al. Self-consistent molecular orbital methods. XXIII. A polarization-type basis set for second-row elements. *J. Chem. Phys.* **77**, 3654-3665 (1982).
17. Gordon, M. S., Binkley, J. S., Pople, J. A., Pietro, W. J. & Hehre, W. J. Self-Consistent Molecular-Orbital Methods. 22. Small Split-Valence Basis Sets for Second-Row Elements. *J. Am. Chem. Soc.* **104**, 2797-2803 (1982).
18. Hariharan, P. C., Pople, J. A. & acta, T. c. The influence of polarization functions on molecular orbital hydrogenation energies. *Theor. Chim. Acta* **28**, 213-222 (1973).
19. Hehre, W. J., Dichtfield, R. & Pople, J. A. Ditchfie. R; Pople, JA, Self-Consistent Molecular-Orbital Methods. 12. Further Extensions of Gaussian-Type Basis Sets for Use in Molecular-Orbital Studies of Organic-Molecules. *J. Chem. Phys.* **56**, 2257-2261 (1972).
20. Spitznagel, G. W., Clark, T., von Ragué Schleyer, P. & Hehre, W. J. An evaluation of the performance of diffuse function-augmented basis sets for second row elements, Na-Cl. *J. Comput. Chem.* **8**, 1109-1116 (1987).
21. Neese, F., Wennmohs, F., Becker, U. & Riplinger, C. The ORCA quantum chemistry program package. *J. Chem. Phys.* **152**, 224108 (2020).
22. Neese, F. Software update: The ORCA program system—Version 5.0. *J. Comput. Chem.* **12**, e1606 (2022).
23. Lin, Y.-S., Li, G.-D., Mao, S.-P. & Chai, J.-D. Long-Range Corrected Hybrid Density Functionals with Improved Dispersion Corrections. *J. Chem. Theory Comput.* **9**, 263-272 (2013).
24. Stein, T., Kronik, L. & Baer, R. Reliable Prediction of Charge Transfer Excitations in Molecular Complexes Using Time-Dependent Density Functional Theory. *J. Am. Chem. Soc.* **131**, 2818-2820 (2009).
25. Stein, T., Eisenberg, H., Kronik, L. & Baer, R. Fundamental Gaps in Finite Systems from Eigenvalues of a Generalized Kohn-Sham Method. *Phys. Rev. Lett.* **105**, 266802 (2010).
26. Karolewski, A., Kronik, L. & Kümmel, S. Using optimally tuned range separated hybrid functionals in ground-state calculations: Consequences and caveats. *J. Chem. Phys.* **138** (2013).
27. Weigend, F. & Ahlrichs, R. Balanced basis sets of split valence, triple zeta valence and quadruple zeta valence quality for H to Rn: Design and assessment of accuracy. *Phys. Chem. Chem. Phys.* **7**, 3297-3305 (2005).
28. Barone, V. & Cossi, M. Quantum Calculation of Molecular Energies and Energy Gradients in Solution by a Conductor Solvent Model. *J. Phys. Chem. A* **102**, 1995-2001 (1998).
29. Janak, J. F. Proof that $\partial E/\partial n_i = \epsilon_i$ in density-functional theory. *Phys. Rev. B: Condens. Matter* **18**, 7165-7168 (1978).
30. Becke, A. D. A new mixing of Hartree–Fock and local density-functional theories. *J. Chem. Phys.* **98**, 1372-1377 (1993).

31. Kodikara, M. S., Stranger, R. & Humphrey, M. G. Long-Range Corrected DFT Calculations of First Hyperpolarizabilities and Excitation Energies of Metal Alkynyl Complexes. *ChemPhysChem* **19**, 1537-1546 (2018).
32. McLean, A. D. & Chandler, G. S. Contracted Gaussian basis sets for molecular calculations. I. Second row atoms, Z=11–18. *The Journal of Chemical Physics* **72**, 5639-5648 (1980).
33. Barone, V. *in Recent Advances in Density Functional Methods, Part I*,°Ed. D. P. Chong (World Scientific Publ. Co., Singapore, 1996).
34. Scalmani, G. & Frisch, M. J. Continuous surface charge polarizable continuum models of solvation. I. General formalism. *J. Chem. Phys.* **132**, 114110 (2010).
35. Sparta, M., Riplinger, C. & Neese, F., Mechanism of Olefin Asymmetric Hydrogenation Catalyzed byIridium Phosphino-Oxazoline: A Pair Natural Orbital Coupled Cluster Study, *J. Chem. Theory Comput.* **10**, 1099-1108 (2014).
36. Stanger, A. Nucleus-independent chemical shifts (NICS): distance dependence and revised criteria for aromaticity and antiaromaticity, *J. Org. Chem.* **71**, 883–893 (2006).
37. Gershoni-Poranne, R., Stanger, A. The NICS-XY-scan: identification of local and global ring currents in multi-ring systems, *Chem. Eur. J.* **20**, 5673–5688 (2014).
38. Geuenich, D., Hess, K., Köhler, F., Herges, R. Anisotropy of the induced current density (ACID), a general method to quantify and visualize electronic delocalization, *Chem. Rev.* **105**, 3758– 3772 (2005).
39. Hashimoto, T., Choe, Y.-K., Nakano, H. & Hirao, K. Theoretical Study of the Q and B Bands of Free-Base, Magnesium, and Zinc Porphyrins,and Their Derivatives, *J. Phys. Chem. A* **103**, 1894-1904 (1999).
40. Jean, Y., Volatron, F., Burdett, J. *An introduction to Molecular Orbitals* page 60 (1993).
